# Beyond RNA modification: a novel role for tRNA modifying enzyme in oxidative stress response and metabolism

**DOI:** 10.1093/nar/gkaf1276

**Published:** 2025-12-12

**Authors:** Louna Fruchard, Claudia Sudol, Caroline Rouard, Aurore Treffkorn-Maurau, Léo Hardy, Julia Bos, Magalie Duchateau, Quentin Giai Gianetto, Mariette Matondo, Frédéric Bonhomme, Quentin Thuillier, Virginie Marchand, Yuri Motorin, Damien Bregeon, Didier Mazel, Djemel Hamdane, Zeynep Baharoglu

**Affiliations:** Epitranscriptomic and translational responses to antibacterial stress Team, Expression Génétique Microbienne, CNRS UMR8261, Institut Pasteur, Université Paris Cité, Institut de Biologie Physico-Chimique, 75005 Paris, France; Institut Pasteur, Université Paris Cité, Unité Plasticité du Génome Bactérien, CNRS UMR3525, 75015 Paris, France; Sorbonne Université, Collège Doctoral, Paris F-75005, France; Sorbonne Université, Collège Doctoral, Paris F-75005, France; Sorbonne Université, CNRS UMR8263, INSERM U1345, Dev2A, IBPS, Paris F-75005, France; Institut Pasteur, Université Paris Cité, Unité des bactéries pathogènes entériques, CNR Vibrions et choléra, Paris 75015, France; Epitranscriptomic and translational responses to antibacterial stress Team, Expression Génétique Microbienne, CNRS UMR8261, Institut Pasteur, Université Paris Cité, Institut de Biologie Physico-Chimique, 75005 Paris, France; Epitranscriptomic and translational responses to antibacterial stress Team, Expression Génétique Microbienne, CNRS UMR8261, Institut Pasteur, Université Paris Cité, Institut de Biologie Physico-Chimique, 75005 Paris, France; Institut Pasteur, Université Paris Cité, Unité Plasticité du Génome Bactérien, CNRS UMR3525, 75015 Paris, France; Institut Pasteur, Université Paris Cité, Proteomics Platform, Mass Spectrometry for Biology Unit, UAR CNRS 2024, Paris 75015, France; Institut Pasteur, Université Paris Cité, Proteomics Platform, Mass Spectrometry for Biology Unit, UAR CNRS 2024, Paris 75015, France; Institut Pasteur, Université Paris Cité, Bioinformatics and Biostatistics Hub, Paris 75015, France; Institut Pasteur, Université Paris Cité, Proteomics Platform, Mass Spectrometry for Biology Unit, UAR CNRS 2024, Paris 75015, France; Institut Pasteur, Université Paris Cité, Epigenetic Chemical Biology Unit, UMR 3523, CNRS, Paris 75015, France; Université de Lorraine, CNRS, INSERM, UMS2008/US40 IBSLor, EpiRNA-Seq Core Facility, Nancy F-54000, France; Université de Lorraine, CNRS, INSERM, UMS2008/US40 IBSLor, EpiRNA-Seq Core Facility, Nancy F-54000, France; Université de Lorraine, CNRS, INSERM, UMS2008/US40 IBSLor, EpiRNA-Seq Core Facility, Nancy F-54000, France; Université de Lorraine, CNRS, UMR7365 IMoPA, Nancy F-54000, France; Sorbonne Université, CNRS UMR8263, INSERM U1345, Dev2A, IBPS, Paris F-75005, France; Institut Pasteur, Université Paris Cité, Unité Plasticité du Génome Bactérien, CNRS UMR3525, 75015 Paris, France; Sorbonne Université, CNRS UMR8263, INSERM U1345, Dev2A, IBPS, Paris F-75005, France; Epitranscriptomic and translational responses to antibacterial stress Team, Expression Génétique Microbienne, CNRS UMR8261, Institut Pasteur, Université Paris Cité, Institut de Biologie Physico-Chimique, 75005 Paris, France

## Abstract

RNA modifications play a fundamental role in regulating essential cellular processes, including translation fidelity and stress adaptation. While these modifications are installed post-transcriptionally by specialized enzymes, their broader functional roles remain largely unexplored. Here, we uncover an unexpected function for the *Vibrio cholerae* tRNA dihydrouridine synthase B (VcDusB) beyond its canonical role in tRNA dihydrouridylation. We show that deletion of *dusB* severely compromises *V. cholerae* resistance to oxidative stress, not through the loss of tRNA modification, but via disruption of an intrinsic NADPH oxidase activity. Mutational analyses reveal that DusB redox function is essential for survival under oxidative stress. Proteomic and transposon insertion sequencing analysis further linked DusB to NADPH homeostasis and metabolic reprogramming during stress adaptation. These findings redefine DusB as a bifunctional enzyme coupling tRNA modification to redox regulation, expanding the functional repertoire of RNA-modifying enzymes in stress adaptation. More broadly, this work paves the way for exploring the evolutionary versatility of tRNA-modifying enzymes, suggesting that their functions extend far beyond RNA metabolism to direct integration of translational control with cellular redox state.

## Graphical Abstract

**Graphical Abstract:**
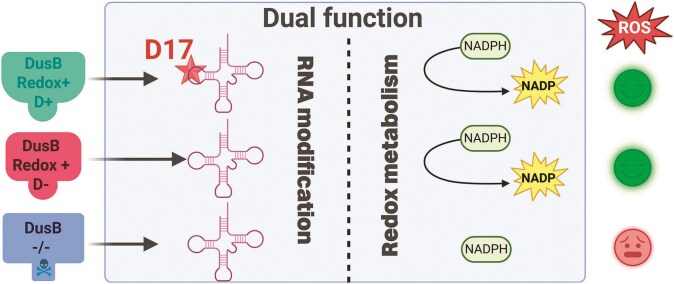

## Introduction

RNAs are the most extensively modified molecules in the cell, with over 170 distinct RNA post-transcriptional modifications identified across various RNA classes, throughout all domains of life, including viruses [1, 2]. Among these, more than 110 occur in tRNAs and are catalyzed by specific tRNA-modifying enzymes [3]. These modifications play crucial roles in influencing various functions [4, 5], including tRNA folding, degradation, and cleavage [6, 7], stability and affinity for other biomolecules [8, 9]. These nucleobase variations ultimately regulate cellular processes such as aminoacylation efficiency and fidelity, and codon-decoding [10–13], reading-frame maintenance, translation speed, efficiency, and accuracy [14]. In addition, tRNA modifications also influence tRNA-derived fragment biogenesis involved in various post-transcriptional processes, notably gene expression regulations.

Studies have emphasized the critical role of tRNA modifications in cellular stress responses [15], particularly in enhancing translation fidelity and modulating stress-induced gene expression [16–21]. We previously demonstrated the importance of tRNA-modifying enzymes in *V. cholerae* adaptation to stress, particularly in response to antibiotics [22, 23].

In response to oxidative stress, such as exposure to hydrogen peroxide, distinct tRNA modifications have been shown to enhance bacterial survival in *Escherichia coli* [24], *Pseudomonas aeruginosa* [20, 25], and *Mycobacterium bovis* bacilli Calmette-Guérin [21], as well as in other systems [26, 27]. Furthermore, RNA modifications exhibit dynamic regulation in response to oxidative stress, underscoring their potential role in bacterial adaptation [26, 28].

While tRNA modifications are crucial for the oxidative stress response, it remains unclear whether the enzymes responsible for these modifications also serve additional functions. Emerging evidence suggests that beyond their established roles in RNA modification [29, 30], some tRNA-modifying enzymes function as tRNA chaperones [31, 32], facilitate aminoacylation [33, 34], regulate virulence [35], metabolism [36], or cell division [37].

Intriguingly, certain tRNA modifications, like dihydrouridine (D), are intrinsically linked to redox metabolism, suggesting a potential vulnerability to oxidative stress that remains largely unexplored [38]. Dihydrouridine synthases (Dus) are FMN-dependent flavoenzymes that reduce uridine to D using NADPH-derived reducing power [39–42].

Proteobacteria encode two or three Dus enzymes; when all three are present, DusA, DusB, and DusC each modify distinct positions within the tRNA D-loop [43]. D is the second most abundant RNA modification in the bacterial and eukaryotic transcriptomes after pseudouridine [1]. The frequency and positioning of D modifications vary depending on tRNA identity, species, and environmental conditions [38, 40]. This modification induces a conformational shift in tRNA structure, influencing base stacking [44, 45] and enhancing the structural flexibility of the tRNA backbone [44, 46], but its role in oxidative stress responses, and the potential moonlighting functions of Dus enzymes remain unexplored.

*Vibrio cholerae*, a pathogen facing oxidative stress during host colonization and in its natural habitats, provides an ideal model to investigate these questions. Here, we report that *V. cholerae* DusB, while responsible D17 biosynthesis in tRNA, plays an unexpected and essential role in bacterial survival under oxidative stress. Strikingly, our findings reveal that *V. cholerae* does not rely on DusB’s canonical tRNA dihydrouridylation activity to withstand oxidative stress. Instead, survival depends on DusB’s alternative redox function, which proves critical under these harsh conditions.

This study provides the first evidence that Dus enzymes serve essential roles beyond tRNA modification, uncovering new layers of their biological significance.

## Materials and methods

### Media and growth conditions

Plating was performed in Mueller-Hinton (MH) agar media at 37°C. Liquid cultures were incubated at 37°C with shaking at 180 rotations per minute, under aerobic conditions in MH.

#### Bacterial strains and complementation/overexpression plasmid constructions

Strains described in this research are derivatives of *Vibrio cholerae* O1 biovar El Tor N16961 *hapR+*, and were generated through allelic exchange. Mutants were generated using *the V. cholerae ∆lacZ* strain (K329) as the background. For plasmid construction, genes along with their native promoters were PCR-amplified from *V. cholerae* genomic DNA and inserted into the pSC101 vector by Sma1 restriction digestion and ligation using T4 DNA ligase. Carbenicillin at 100 μg/ml was used for selection during the cloning process. Supplementary Tables S4–S6 provide further details on the strains, plasmids, and primers. For the *ibpA* reporter strains (W962 and W963), endogenous inclusion body protein A *ibpA* was replaced by natural transformation by a cassette containing a gene fusion of *Vibrio cholerae ibpA* to the yellow fluorescent protein YFP and a kanamycin resistance gene (*yfp* from the fusion strain of AB Lindner [47].

#### Measurement of intracellular H₂O₂ levels using roGFP2 biosensor

pQE-60 roGFP2-His was a gift from Tobias Dick (Addgene plasmid # 65 046; http://n2t.net/addgene:65046;  RRID:Addgene_65 046) [48]. Overnight cultures of wild-type and mutant strains were grown in MH medium supplemented with carbenicillin, and aliquots were either left untreated or treated for 10 min with DTT 10 mM (fully reduced control) or H_2_O_2_ 1 mM (fully oxidized control). For flow cytometry, 200 µL PBS was distributed into wells or tubes, and 5 µL of each overnight culture was added. roGFP2 fluorescence was measured on a flow cytometer using excitation at 405 nm with emission at 510 nm (V2 channel, oxidized signal) and excitation at 488 nm with emission at 510 nm (B1 channel, reduced signal). To correct for spectral overlap, the B1 signal was subtracted from the V2 signal, and the 405/488 fluorescence ratio was calculated. The degree of oxidation (OxD) was determined using the formula: OxD = (R − Rred) / (Rox − Rred), where R is the fluorescence ratio of the sample, and Rred and Rox are ratios obtained under fully reduced and fully oxidized conditions, respectively. Steady-state OxD values were then compared between strains to assess intracellular H₂O₂ levels.

#### Construction and implementation of the NADPH biosensor in *Vibrio cholerae*

We adapted the Rex-based ratiometric NADPH biosensor NAPStar1 [49] for expression in *V. cholerae*. The sensor architecture comprises an *N*-terminal Rex domain, a circularly permuted T-Sapphire (cpT-Sapphire) reporter, a *C*-terminal Rex domain, and an mCherry reporter. The domains were connected using the following linkers: SAAGGHG (between the *N*-terminal Rex and cpT-Sapphire), T (between cpT-Sapphire and the *C*-terminal Rex), and GSGTGGNASDGGGSGG (between the *C*-terminal Rex and mCherry). The open reading frame sequence (NAPStar1) used in this study is provided in Supplementary Table S7.

To optimize expression in *V. cholerae*, we codon-adapted the entire sensor ORF and placed it under the P*trc* promoter, which is constitutive in *V. cholerae*. The complete promoter–sensor cassette was synthesized de novo (Twist Bioscience) and cloned into the *V. cholerae*–compatible pTOPO plasmid (∼5-copy in this species). Plasmids were first propagated in *E. coli*, then introduced into *V. cholerae* by electroporation, and transformants were selected on kanamycin 25 µg/ml. Cultures were grown in MH + kanamycin at 37°C with aeration.

For flow cytometry, cells were analyzed live and gated on mCherry-positive events (plasmid-containing cells). Fluorescence was recorded for cpT-Sapphire using a violet (≈405 nm) excitation and 520 nm emission, and for mCherry using a yellow-green (≈561 nm) excitation with 610–630 nm emission (peak ≈ 619 nm). Sensor output was quantified as the cpT-Sapphire intensity normalized to mCherry (cpT-Sapphire/mCherry) to account for expression variability. All experiments included biological replicates, n > 8.

#### Survival/killing assays

Survival assays were conducted using cultures in the early exponential phase. Overnight stationary phase cultures were diluted 1000-fold and grown in 20 mL of fresh MH medium in an Erlenmeyer flask at 37°C, in aerobic conditions, with 180 rotations per minute, until the optical density at 600 nm (OD_600_) reached 0.2–0.25. At time zero, aliquots of the untreated cultures were plated on MH agar to establish the initial CFU count. For the assays, 5 mL of culture was transferred to 14 mL Falcon tubes and exposed to lethal doses of hydrogen peroxide (1–2 mM) for various durations (10 min, 30 min, 1 h, and 2 h) at 37°C with shaking to ensure adequate oxygenation. After treatment, appropriate dilutions were plated on MH agar to determine bacterial count. The survival rate was then calculated by normalizing the bacterial concentration after treatment to the initial CFU count. Each experiment was replicated between 3 and 15 times.

### tRNA decay curve measurement

*V. cholerae* cultures were diluted 1:1000 into MH medium and grown to OD_600_ ≈ 0.2. Rifampicin (100 µg/ml) was added, and 30 ml samples were collected at defined time points during exponential (0 min, 30 min, 1–4 h) and early stationary phase (0–60 min) growth. Pellets were resuspended in TRIzol, and RNA was extracted using our tRNA-enrichment protocol (see below). Total RNA (1–10 µg) was deacylated in 100 mM Tris-HCl (pH 9, 30 min, 37°C) prior to electrophoresis. RNAs were resolved on denaturing polyacrylamide gels, stained, transferred to nylon membranes, and UV-crosslinked. Northern blotting was performed with 3′-DIG–labeled probes specific for tRNA-Gly-GCC, tRNA-AlaTGC, and the control tRNA-Ser-GCT (also detecting tRNASer-GGA); 5S rRNA served for normalization. Probe sequences are provided in Supplementary Table S7. Detection was achieved by chemiluminescence, and membranes were stripped and re-probed as required.

#### Nanopore tRNA-seq

This was performed on total RNA extracted from ∆*dusB* pDusB+, ∆*dusB* pDusBm6 and ∆*dusB* p0 (emptly plasmid). Nanopore tRNA-seq and bioinformatic analysis were performed by Immagina Biotechnology, as described:

https://github.com/ImmaginaBiotechnology/Documents/blob/main/NanotRNAseq.md. The analysis pipeline comprised four sequential steps. First, basecalling was performed on POD5 files generated by MinKNOW using Dorado (v0.7.2 or newer), producing a single BAM file containing processed reads. Second, the reads were aligned to the reference tRNA database with Minimap2 (v2.24 or newer), yielding a BAM file of aligned reads. Third, demultiplexing was carried out with the DeMuxnanoT app, generating one BAM file per sample (assuming six samples per library). Finally, quantification and error analysis were performed for each sample, including (i) determination of tRNA counts relative to the reference database and (ii) calculation of basecalling error rates (mismatches with the reference sequence), which served as indicators of RNA modifications.

#### tRNA-enriched RNA extraction

Overnight cultures of *V. cholerae* were diluted 1000-fold in MH medium and incubated under aerobic conditions, at 37°C, with shaking at 180 rotations per minute until an OD_600_ of 0.25. tRNA-enriched RNA extracts were prepared using TRIzol reagent at room temperature (RT) as previously described [23]. DNA contamination was removed using the TURBO DNA-free Kit (Ambion) according to the manufacturer’s instructions. RNA concentration was measured using a NanoDrop 2000c (Thermo Fisher Scientific), and the quality of the isolated tRNA fractions was evaluated by capillary electrophoresis with a RNA 6000 Pico chip on Bioanalyzer 2100 (Agilent Technologies).

#### Analysis of RNA modifications by AlkAniline-sequencing

AAS was conducted previously described in [50, 51][52]. Total RNA (∼200 ng) from *V. cholerae* cells was subjected to random fragmentation by alkaline hydrolysis in 50 mM sodium-bicarbonate buffer at pH 9.2 and 96°C for 5 min. The reaction was stopped by ethanol precipitation using 3M Na-OAc, pH 5.2, and glycoblue. After centrifugation, the RNA pellet was washed with 80% ethanol and resuspended in nuclease-free water. RNA fragments were de-phosphorylated by Antarctic phosphatase (NEB ref M0289L, USA) at 37°C for 1 h and precipitated using 3M Na-OAc, pH 5.2, and glycoblue as previously described [53]. After centrifugation, the RNA pellet was washed with 80% ethanol, and the pellet was resuspended in 1M Aniline pH 4.5 and incubated for 15 min at 60°C in dark. The reaction was stopped by ethanol precipitation using 3M Na-OAc, pH 5.2, and glycoblue. The pellet was washed twice with 80% ethanol, dried, and resuspended in 3.5 µL of nuclease-free water. RNA fragments were converted to a library using the NEBNext Small RNA Library Prep Set for Illumina® (NEB ref E7330S, USA) following the manufacturer’s recommendations. The DNA library was quantified using a fluorometer (Qubit 2.0 fluorometer, Invitrogen, USA) and qualified using a High Sensitivity DNA chip on Agilent Bioanalyzer 2100. Libraries were multiplexed and subjected to high-throughput sequencing on an Illumina NextSeq2000 instrument with a 50 bp single-end read mode.

High-quality raw sequencing reads (> Q30) were subjected to trimming using Trimmomatic v0.39 [54][55][56] with the following parameters: MINLEN:08, STRINGENCY:7, AVGQUAL:30, trimmed reads were used for alignment without further processing. Trimmed reads were aligned to the *V. cholerae* rRNA/tRNA reference sequence obtained from gtRNAdb, in end-to-end mode (–no-unal –no-1mm-upfront -D 15 -R 2 -N 0 -L 10 -i S,1,1.15 as other bowtie2 parameters), only uniquely mapped reads in positive orientation were retained for further analysis. 5′-reads’ extremities were counted for each RNA position in the reference; all further steps were performed in R/R-studio environment. After a −1 shift in the sequence position, since the ligated 5′-P extremity is at the N + 1 nucleotide in the RNA sequence, this reads’ count was used as the measure for intensity of cleavage at a given position. Four scores were used for analysis of AlkAnilineSeq raw data [50, 51, 54, 55]: Normalized cleavage (NCleavage), normalized count (NormCount), normalized G count (NormGcount), and stop ratio. Normalized cleavage corresponds to a 1000x proportion of reads starting at a given position to the total number of reads mapped to a given RNA sequence. This score is less noisy but also less sensitive than others and is well suited for the detection of major cleavage events in RNA. In contrast, the stop ratio (closely derived from the ψ-ratio used for analysis of Ψ-seq data [56]) is calculated as a ratio of reads starting at a given position to the total number of reads passing (covering) it. This score is relatively sensitive, but also noisy. The last two scores used, NormCount and NormGcount, use local normalization to the median of cleavage signals in -/+ 5 nt window around of analyzed position. NormCount uses all cleavage signals in the window, while signals corresponding to G residues are excluded from the calculation of the normalization median for NormGcount. These scores represent the intensity of the cleavage at a given nucleotide compared to the local cleavage background in the adjacent RNA region. None of the AlkAnilineSeq scores show linear dependence between the score’s value and stoichiometry of RNA modification, stop ratio has a linear segment at very low modification levels (<5%), while the three other scores better represent the modification stoichiometry at higher modification levels in RNA (5–50%).

### Availability of datasets

Raw AlkAnilineSeq data are available at the European Nucleotide Archive (https://www.ebi.ac.uk/ena/browser/home) under the accession number PRJEB88079.

#### tRNA-enriched samples digestion for quantitative analysis of dihydrouridine by mass spectrometry

Purified tRNA-enriched RNA fractions were digested into single nucleosides using the Nucleoside digestion mix from New England BioLabs (Cat No. M0649S). Each 10 µL aliquot of RNA, diluted to 100 ng/µL in ultrapure water, was combined with 1 µL of enzyme and 2 µL of 10X Nucleoside Digestion Mix Reaction Buffer, making up a final volume of 20 µL in nuclease-free 1.5 mL tubes. The tubes were sealed with parafilm to prevent evaporation and incubated at 37°C overnight.

#### D quantification by LC-MS/MS

Analysis of global levels of dihydrouridine (D) was performed on a Q exactive mass spectrometer (Thermo Fisher Scientific). It was equipped with an electrospray ionization source (H-ESI II Probe) coupled with an Ultimate 3000 RS HPLC (Thermo Fisher Scientific).

Digested RNA was injected onto a ThermoFisher Hypersil Gold aQ chromatography column (100 mm * 2.1 mm, 1.9 μm particle size) heated at 30°C. The flow rate was set at 0.3 mL/min and run with an isocratic eluent of 1% acetonitrile in water with 0.1% formic acid for 10 min. Parent ions were fragmented in positive ion mode with 10% normalized collision energy in parallel-reaction monitoring (PRM) mode. MS2 resolution was 17 500 with an AGC target of 2e5, a maximum injection time of 50 ms, and an isolation window of 1.0 m/z.

The inclusion list contained the following masses: U (245.1) and D (247.1). Extracted ion chromatograms of base fragments (±5 ppm) were used for detection and quantification (113.0349 Da for U; 115.0505 Da for D). Calibration curves were previously generated using synthetic standards (CliniSciences, France) in the ranges of 0.2–40 pmol injected for U and 0.01–1 pmol for D. Results are expressed as a percentage of total U.

#### Construction of *gfp* reporters with codon stretches

The positive control was *gfp*mut3 (a stable *gfp* variant) [57] under the control of *Vc* PgyrA promoter. Synthetic fragments carrying the promoter, the desired tRNA sequence, and the *gfp*mut3 STOP codon were ordered from IDT as double-stranded DNA gBlocks and cloned into pTOPO blunt plasmid. The reporter sequence is detailed below, with the −35, −10 boxes and the ATG start and TAA stop codons indicated in bold and underlined, and the stretch insertion site is shown in italics.

TGACTTGGCGCTCAATCTTGTAGTGAGCTTCGTTT CAGTAAGAATTTGGGTATACCGATCAAACTATAGA GGGATAATGGCTCTATG  *(±stretches)*CGTAAAGGAGAA GAACTTTTCACTGGAGTTGTCCCAATTCTTGTTGAATTAGATGGTGATGTTAATGGGCACAAATTTTCTGTCAGTGGAGAGGGTGAAGG TGATGCAACATACGGAAAACTTACCCTTAAATTTATTT GCACTACTGGAAAACTACCTGTTCCATGGCCAACACT TGTCACTACTTTCGGTTATGGTGTTCAATGCTTTG CGAGATACCCAGATCATATGAAACAGCATGACTTT TTCAAGAGTGCCATGCCCGAAGGTTATGTACAGGAA AGAACTATATTTTTCAAAGATGACGGGAACTACAA GACACGTGCTGAAGTCAAGTTTGAAGGTGATACCC TTGTTAATAGAATCGAGTTAAAAGGTATTGATTTT AAAGAAGATGGAAACATTCTTGGACACAAATTGGA ATACAACTATAACTCACACAATGTATACATCATGG CAGACAAACAAAAGAATGGAATCAAAGTTAACTTC AAAATTAGACACAACATTGAAGATGGAAGCGTTCA ACTAGCAGACCATTATCAACAAAATACTCCAATTG GCGATGGCCCTGTCCTTTTACCAGACAACCATTAC CTGTCCACACAATCTGCCCTTTCGAAAGATCCCA ACGAAAAGAGAGACCACATGGTCCTTCTTGAGTTT GTAACAGCTGCTGGGATTACACATGGCATGGATGA ACTATACAAATAA

To test amino-acid incorporation efficiency at a specific codon, three repetitions of the target codon sequence were inserted immediately after the ATG start codon of the *gfp* gene. Double-stranded DNA fragments were synthesized as *eblocks* by Integrated DNA Technologies (IDT), and subsequently cloned into the pTOPO-Blunt vector, including kanamycin resistance, following the manufacturer’s guidelines.

#### Quantification of *gfp* fusion expression by fluorescent flow cytometry

Following plasmid purification using Miniprep, the plasmids were transformed into the target strains. Flow cytometry experiments were performed on overnight cultures, as previously described [58], and repeated a minimum of six times.

For each experiment, 50 000–100 000 events were recorded using the Miltenyi MACSquant flow cytometer. Mean fluorescence per cell was measured in the FITC channel for each reporter in both wt and *ΔdusB* strains. The ∆∆fluorescence ratio, enabling robust comparison of codon decoding efficiency between wt and knockout strains, is obtained as follows: the fluorescence measured in ∆*dusB* for a reporter containing codon stretches is initially divided by the average fluorescence obtained for the same reporter in the wt strain; this ratio (referred to as ∆fluorescence_codon X_) is then normalized against the ∆fluorescence control ratio obtained from the wt *gfp* reporter, without codon stretches (∆fluorescence_wt gfp_). Normalization eliminates biases due to differential Gfp translation between wt and ∆*dusB* strains, ensuring an accurate measure of fluorescence changes.

#### Expression and purification of dus proteins

Chemically competent *E. coli* BL21DE3 star cells transformed with the pET15b-*dus* plasmid were grown in LB (Lysogenic Broth) medium supplemented with ampicillin (100 µg/mL) at 37°C, until the optical density at 600 nm reached 0.6. Protein synthesis was induced by the addition of isopropyl-β-D-thiogalactoside (IPTG) to a final concentration of 1 mM. Cells were grown overnight at 16°C, collected by centrifugation (3500 *x g* at 4°C for 25 min), and stored at −80°C until use. Cells were re-suspended in 50 mM sodium phosphate buffer, pH 8, containing 300 mM NaCl, 5 mM dithiothreitol (DTT), 25 mM imidazole, 10% glycerol (v/v), 1X EDTA-free protease inhibitor cocktail tablet (Roche), and discontinuously sonicated for 15 min in a water ice batch. Cellular extracts were centrifuged for 1 h at 15,000xg, which yielded a soluble fraction of DusB proteins. The soluble fraction was loaded on a Ni-NTA column (Qiagen) previously equilibrated with 50 mM sodium phosphate, pH 8, containing 300 mM NaCl, 25 mM imidazole, 10% glycerol (v/v) (buffer A). After extensive washing with buffer A, the protein was eluted with buffer A supplemented with 250 mM imidazole. Fractions containing DusB proteins were pooled and concentrated by ultrafiltration. Protein was loaded onto a HiLoad 16/600 Superdex 75 equilibrated with 50 mM Tris-HCl, pH 8, 250 mM NaCl, 1 mM DTT. Exchange buffer was conducted on PD-10 Desalting Columns containing Sephadex G-25 resin equilibrated in 50 mM HEPES, pH 7.5, 150 mM NaCl, and 15% glycerol (v/v). Purity of the proteins was assessed by sodium dodecyl sulphate-polyacrylamide gel electrophoresis (SDS-PAGE). Finally, proteins were flash frozen in liquid nitrogen and stored at −80°C until use. Protein concentrations were determined by Bradford assay (Biorad) with BSA used as a standard.

#### NADPH oxidase activity

The capacity of VcDusB and its mutants to oxidize NADPH under steady-state conditions was assessed in the presence of air, which served as the final electron acceptor. The experiments were conducted in a buffer containing 50 mM HEPES at pH 7.5, 150 mM NaCl, and 15% (v/v) glycerol. Reactions were carried out using 1.5 µM of VcDusB protein in the presence of 160 µM NADPH. The extent of NADPH oxidation was monitored by measuring the decrease in absorbance at 343 nm (ε343 = 6.21 mM-1.cm-1). The initial reaction rate as a function of NADPH concentration was analyzed using Michaelis-Menten kinetics.

#### Electrophoretic mobility shift assays for tRNA binding

Electrophoretic mobility shift assays were carried for VcDusB and its different mutants using a 6% native (19:1) PAGE at 4°C with 100 V. Increased concentrations of proteins were added to a fix concentration of RNA bulk extracted from the strain of *V. cholerae* deleted for VcDusB (2 μM) and incubated at room temperature for 20 min in 50 mM Tris–HCl pH 8, 10% glycerol, 5 mM DTT, and 150 mM ammonium acetate prior to migration. RNA was visualized by toluidine coloration.

#### Total RNA purification

For total RNA extraction, overnight cultures were diluted 1000-fold in MH medium and incubated in triplicate with rotation at 37°C until reaching an OD_600_ of 0.2–0.25, indicating early exponential growth. A 2 mL aliquot of each culture was centrifuged in an RNase-free Eppendorf tube, and the supernatant was discarded. The resulting pellet was resuspended in 1.5 mL of TRIzol Reagent at RT, and 300 μL of chloroform was added to the samples, which were then vortexed to ensure thorough mixing. The samples were then centrifuged at 4°C for 10 min. The aqueous (upper) phase was carefully transferred to a clean 2 mL RNase-free tube and mixed with an equal volume of 70% ethanol. The resulting solution was loaded onto a RNeasy Mini Kit (Qiagen) column for RNA purification, following the manufacturer’s protocol. Contaminating DNA was eliminated using the TURBO DNA-free Kit (Ambion), according to the manufacturer’s instructions. Quality of total RNA samples was controlled using the Agilent RNA 6000 nano kit, following the manufacturer’s protocol.

#### RNA-seq

Sample collection and total RNA extraction were performed as previously described [23, 28]. For library preparation, Illumina® Stranded Total RNA Prep, Ligation with Ribo-Zero Plus, Microbiome kit was used with the addition of custom probes targeting *Vibrio cholerae*. For sequencing, quality control was performed on iSeq100 and production run on NextSeq2000, with 5 million reads per sample. The RNA-seq analysis was performed with Sequana 0.16.11 [59]. We used the RNA-seq pipeline 0.19.2 (https://github.com/sequana/sequana_rnaseq) built on top of Snakemake 7.32.4. Briefly, reads were trimmed from adapters using Fastp 0.22.0 [60] then mapped to the *Vibrio_cholerae*_N16961 using bowtie2 2.4.5. FeatureCounts 2.0.1 was used to produce the count matrix, assigning reads to features using the corresponding annotation from MaGe with strand-specificity information. Quality control statistics were summarized using MultiQC 1.17 [61]. Statistical analysis on the count matrix was performed to identify differentially regulated genes. Clustering of transcriptomic profiles was assessed using a Principal Component Analysis (PCA). Differential expression testing was conducted using DESeq2 library 1.34.0 scripts, indicating the significance (Benjamini-Hochberg adjusted *P*-values, false discovery rate FDR < 0.05) and the effect size (fold-change) for each comparison. The data for this RNA-seq study have been submitted to the GenBank repository under the project number GSE288937.

#### Protein extraction

Overnight cultures were diluted 100-fold in MH medium and grown in triplicate or five replicates with rotation at 37°C until reaching an OD_600_ of 0.25 (exponential phase). Cultures were then treated or not with 1 mM H_2_O_2_ for 10 min. 200 mL of these cultures were centrifuged for 10 min at 4°C, and the supernatant was discarded. Cells were lysed by incubation in lysis buffer (10 mM Tris-HCl, pH 8, 150 mM NaCl, 1% Triton 100X) supplemented with 0.1 mg/mL lysozyme and complete EDTA-free Protease Inhibitor Cocktail (Roche) for 1 h on ice. Lysates were then sonicated 3 × 50 s (power: 6, pulser: 90%), centrifuged at 5000 rpm for 2 h at 4°C, and supernatants were quantified using Pierce™ BCA Protein Assay Kit (Cat. No 23 225) following the manufacturer’s instructions. Proteins were then stored at −80°C.

### Proteomics MS and analysis

*Sample preparation for MS*. Samples were processed using the S-Trap^TM^ micro column (Protifi, Huntington, USA) according to the manufacturer’s instructions with minor modifications. Briefly, 50 µg of sample was resuspended in 5 mM TCEP, 20 mM chloroacetamide, 5% SDS, followed by incubation at room temperature for 30 min. Phosphoric acid was then added to a final concentration of 2.5%, followed by the addition of binding/washing buffer (100 mM TEAB in 90% methanol). The mixtures were then applied to S-Trap columns and washed four times for thorough SDS removal. Finally, proteins were digested with 5 μg sequencing-grade modified trypsin (Promega) overnight at 37°C. After elution, peptides were concentrated to dryness and resuspended in 2% acetonitrile (ACN)/0.1% FA just prior to LC-MS injection.

*LC-MS/MS analysis*. Samples were analyzed on a high-resolution Q Exactive™ Plus Mass Spectrometer (Thermo Scientific), coupled to a Proxeon EASY 1200 nLC system (Thermo Fisher Scientific, Bremen). One microgram (µg) of peptides was injected onto a home-made 50 cm C18 column (1.9 μm particles, 100 Å pore size, ReproSil-Pur Basic C18, Dr. Maisch GmbH, Ammerbuch-Entringen, Germany). A 66 cm column was used for the non-treated samples, and a 43 cm column for the H_2_O_2_-treated samples. Column equilibration and peptide loading were performed at 900 bars in buffer A (0.1% FA). Peptides were separated using a multi-step gradient from 3% to 22% buffer B (80% acetonitrile, 0.1% formic acid) over 160 min, then from 22% to 50% over 70 min, and finally from 50% to 90% over 5 min, at a flow rate of 250 nL/min. The column temperature was maintained at 60°C. The Q Exactive™ Plus Mass Spectrometer (Thermo Scientific) was operated in data-dependent mode using a Full MS/ddMS2 Top 10 experiment (35 s dynamic exclusion). MS scans were acquired at a resolution of 70 000 and MS/MS scans (fixed first mass 100 m/z) at a resolution of 17 500. The AGC target and maximum injection time for the survey scans and the MS/MS scans were set to 3E6, 20ms and 1E6, 60ms respectively. The isolation window was set to 1.6 m/z, and the normalized collision energy was fixed to 27 for HCD fragmentation. We used an underfill ratio of 1.0% corresponding to an intensity threshold of 1.7E5. Unassigned precursor ion charge states as well as 1, 7, 8, and > 8 charged states were rejected, and peptide match was disabled.

*Data analysis*. Acquired Raw data were analyzed using MaxQuant 2.1.1.0 version [62] using the Andromeda search engine [63] against*Vibrio cholerae* Uniprot reference proteome database (3782 entries, download date 2020–02-21) concatenated with usual known mass spectrometry contaminants and reversed sequences of all entries. All searches were performed with oxidation of methionine and protein *N*-terminal acetylation as variable modifications and cysteine carbamidomethylation as a fixed modification. Trypsin was selected as a protease allowing for up to two missed cleavages. The minimum peptide length was set to 5 amino acids, and the peptide mass was limited to a maximum of 8000 Da. The false discovery rate (FDR) for peptide and protein identification was set to 0.01. The main search peptide tolerance was set to 4.5 and 20 ppm for the MS/MS match tolerance. One unique peptide to the protein group was required for the protein identification. A false discovery rate cut-off of 1% was applied at the peptide and protein levels. All mass spectrometry proteomics data have been deposited at ProteomeXchange Consortium via the PRIDE partner repository with the dataset identifiers PXD057105, PXD057145, and PXD057061.

The statistical analysis of the proteomics data was performed as follows: three replicates were acquired for the H_2_O_2_-treated samples, and five biological replicates were acquired for the non-treated samples. To highlight significantly differentially abundant proteins between two conditions, differential analyses were conducted through the following data analysis pipeline: (i) deleting the reverse and potential contaminant proteins; (ii) keeping only proteins with at least two or three quantified values in one of the three or five compared conditions to limit misidentifications and ensure a minimum of replicability; (iii) log2-transformation of the remaining intensities of proteins; (iv) normalizing the intensities by median centering within conditions thanks to the normalizeD function of the R package DAPAR [64], (v) putting aside proteins without any value in one of both compared conditions: as they are quantitatively present in a condition and absent in another, they are considered as differentially abundant proteins and (vi) performing statistical differential analysis on them by requiring a minimum fold-change of 2.5 between conditions and by using a LIMMA t test combined [65] with an adaptive Benjamini–Hochberg correction of the *P*-values thanks to the adjust.p function of the R package cp4p [66]. The robust method of Pounds and Cheng was used to estimate the proportion of true null hypotheses among the set of statistical tests [67]. The proteins associated with an adjusted *P*-value inferior to an FDR level of 1% have been considered as significantly differentially abundant proteins. Finally, the proteins of interest are therefore the proteins that emerge from this statistical analysis, supplemented by those being quantitatively absent from one condition and present in another.

### Frameshift and readthrough quantification assay

*Dual reporter system*. To evaluate readthrough or frameshifting levels, we utilized the bacterial pCL99 *lacZ-luc* dual reporter system, as outlined by Namy *et al.* [68]. This system involves the fusion of the *luc* gene, which encodes the luciferase, to the *lacZ* gene, encoding the β-galactosidase. For readthrough frequency quantification, the *luc* gene can be positioned either in the same open reading frame as *lacZ*, with a Lys-AAG codon between the two genes (in-frame), or separated from *lacZ* by a stop codon (TGA, TAA, or TAG) (sequences detailed in Supplementary Fig. S4). For frameshifting evaluation, the *luc* gene is placed either in the same reading frame (in-frame), or shifted by −1 or + 1 frames relative to *lacZ* (-1 or + 1-frameshift) (sequences detailed in Supplementary Fig. S3A). Readthrough and frameshifting frequencies are determined by measuring the luciferase activity and normalizing it to the β-galactosidase activity, which serves as an internal control for translation efficiency. The readthrough ratio is multiplied by 1000, while the frameshifting ratio is multiplied by 100 to express the results as percentages.

*Samples preparation and quantification. V cholerae* electrocompetent cells were transformed with the plasmids described above. Overnight cultures of the transformants were diluted 1000-fold in MH medium supplemented with 100 μg/ml carbenicillin for pSC101 maintenance, 5 μg/ml chloramphenicol for pCL maintenance, 200 μg/ml IPTG (Isopropyl β-D-1-thiogalactopyranoside) for reporter induction. Cultures were then incubated with agitation at 37°C until an OD_600_ of 0.25 (exponential phase). Luciferase luminescence was quantified using the luciferase assay system (Promega, WI, USA). Briefly, 90 μl of each culture was aliquoted into 1.5 ml tubes, treated with 10 μl K2HPO4 (1M) and EDTA (20 mM), and quick-frozen in dry ice for 1 min. The tubes were then thawed by placing them in room-temperature water for 5 min. Around 300μl of lysis buffer (CCLR 1X; lysozyme 1.25 mg/ml; BSA 2.5 mg/ml) was added to the tubes that were placed back in water for 10 min. Subsequently, 100 μL of the lysate was mixed with 100 μL of Luciferase Assay Reagent in 5 mL tubes, and luminescence was measured for 10 s using a Lumat LB 9507 (EG&G Berthold).

For β-galactosidase activity quantification, 2 ml of the cultures were aliquoted and mixed with 50 μl chloroform and 50μl SDS 0.1%. After vortexing for 45 s, samples were placed 5 min at RT for cell lysis. Next, 500 μl of the lysates were collected into 5 ml tubes and treated with 1.5 ml Z-Buffer (8.5 mg/ml Na2HPO4; 5.5 mg/ml NaH2PO4H2O; 0.75 mg/ml KCl; 0.25 mg/ml MgSO4,7H2O) supplemented with 7μl/ml 2-Mercaptoethanol. After 5-min incubation at 37°C, 500 μl ONPG (4 mg/mL) was added to the samples, and the reaction was allowed to proceed at 37°C for 1 h. Reaction was finally stopped by adding 1ml Na2CO3 (1M) to the tubes. The resulting 2 mL suspension was transferred into Eppendorf tubes, centrifuged, and the absorbance of the supernatant was measured at 420 nm.

#### Transposon insertion sequencing

Libraries were prepared as previously described [22]. The mutant library was generated by conjugating the donor *E. coli* strain F656 (B2163, DAP-auxotroph), carrying the pSC189 plasmid encoding the mariner transposon, with the K329 WT or *ΔdusB* mutant strain. Fifty milliliters of each culture (donor and recipient) were mixed, centrifuged, and the resulting pellet was resuspended in 1 mL of LB. Then, 200 µL of the suspension was spotted directly onto five conjugation filters placed on fresh LB + DAP plates. After 3 h of incubation at 37°C, the filters were pooled, resuspended in 8 mL of LB, and plated onto eight LB + Spec200 plates to select for *Vibrio* transconjugants carrying the transposon. Plates were incubated overnight at 37°C. To estimate the number of mutants in the final library and assess conjugation efficiency, 10-fold serial dilutions of the suspension were plated on both selective and non-selective media. This procedure yielded approximately 1 × 10^6^ transconjugants. Colonies from the eight selection plates were scraped, pooled, and resuspended in 2.5 mL of LB. The library was then cryopreserved in 10% DMSO for further use. For Tn-Seq screening, an aliquot of the frozen library was thawed and diluted to reach an OD_600_ of 2, corresponding to an overnight culture. Three 1-mL aliquots were harvested by centrifugation to constitute the T0 triplicates. In parallel, the library was diluted 1:1000 into Mueller-Hinton medium and cultured in triplicate for 16 generations. At the end of the growth, 1 mL was collected from each replicate and pellet to obtain the T16 triplicates.

DNA libraries for sequencing were prepared as previously described [69]. Genomic DNA (gDNA) was extracted directly from cell pellets using the Wizard Genomic DNA Purification Kit (Promega), following the manufacturer’s protocol. The extracted DNA was then fragmented via sonication using a Covaris E220 instrument (parameters: duty cycle 5, burst cycle 200, peak power 105, duration 40 s), in 130 µL AFA Fiber Screw-Cap microtubes (Covaris). To eliminate short DNA fragments, the sheared gDNA was purified by adding 0.6 × volume of Agencourt Ampure XL magnetic beads (Beckman Coulter), according to the supplier’s instructions. This process yielded DNA fragments ranging from 300 to 1000 base pairs. Cytosine homopolymer tails (C-tails) were appended to the 3′ ends of the DNA using recombinant terminal deoxynucleotidyl transferase (rTdT; 30 U/μl, Promega). Size-selected DNA was incubated with the enzyme at 37°C for 1 h, and the reaction was inactivated at 75°C for 20 min. The TdT reaction products were cleaned using a 1 × volume of Ampure XL beads.

To amplify transposon–genome junctions, an initial PCR (PCR1) was carried out in a 50 μL reaction containing the C-tailed DNA, 1 μL of biotin-labeled pSC189-PCR1 primer (30 μM), 3 μL of olj376 primer (30 μM), 2.5 μL of dNTP mix (10 mM), 10 μL of Q5 reaction buffer, and 0.75 μL of Q5 high-fidelity polymerase (New England Biolabs). PCR1 products were purified using 1 × Ampure beads. Biotinylated amplicons were then captured using MyOne Streptavidin T1 Dynabeads (Invitrogen), following the manufacturer’s instructions.

A second PCR (PCR2) was performed directly on the bead-bound templates using a mix composed of 1 μL pSC189-PCR2 primer (30 μM), 1 μL BCXXXX primer (30 μM), 2.5 μL dNTPs (10 mM), 10 μL Q5 buffer, and 0.75 μL Q5 polymerase, in a final volume of 50 μL. PCR2 products were purified with 1 × Ampure XL beads. Final libraries were sequenced at the Biomics Platform (Institut Pasteur) on an Illumina NextSeq2000 system using the custom primer ReadTnLpSC189.

Between 5 000 000 and 1 000 000 reads were obtained per sample. The transposon sequence at the beginning of each read, and the poly-C tail at the end, were trimmed using tools available on the Galaxy Project’s public server. Trimmed reads longer than 16 bases were mapped to the reference genome using the Tn-seq PreProcessor (TPP) software. The resulting .wig files were used to perform essentiality analysis with TRANSIT [70]. Conditional essentiality analysis was performed using the “resampling” method according to the TRANSIT documentation. Accession numbers for TN-seq: BioProject: PRJNA1331814

(https://www.ncbi.nlm.nih.gov/bioproject/PRJNA1331814)

#### Cytoplasmic pH measurement

The fluorescent pH-sensitive pHrodo dye (560/590; Ex/Em) (Thermofisher Scientific #P35372) was used according to the manufacturer’s protocol to monitor intracellular pH change (https://www.thermofisher.com/fr/fr/home/references/newsletters-and-journals/bioprobes-journal-of-cell-biology-applications/bioprobes-70/phrodo-ph-sensors.html?utm_source=chatgpt.com). Optical density (OD_600_) and red fluorescence were measured over time using a TECAN plate reader. For each sample, fluorescence intensity was normalized to OD_600_.

#### Microscopy imaging

Bacterial cells with *ibpA*-*YPF* chromosomal gene fusion replacing endogenous *ibpA* were grown to early exponential phase in liquid MH and were treated or not with 500 µM H_2_O_2_ for 10 min. After treatment, cells were centrifuged (10 000 rpm, 3 min) to remove excess pHrodo dye, and pellets were resuspended in DPBS. A 0.8 µL volume of cells was transferred onto 1.3% agarose-padded slides containing MH medium. A coverslip was placed on top of the agarose pad and sealed with a vaseline:lanolin:paraffin mix (ratio 1:1:1) to prevent pad evaporation. Microscopy slides were placed under a Zeiss ApoTome inverted wide-field microscope. Images were captured in the phase contrast channel and TRITC channel (150 ms exposure time), using a Plan Apo 63 × objective (numerical aperture = 1.4, +optovar 1.6×) using a Hamamatsu sCMOS ORCA-Flash 4.0 v3 (Pasteur Institute Imaging Facility Imagopole). Image analysis was performed using FIJI software with Microbe J plugin [71][72]. The foci rate per cell (%) was calculated as the number of IbpA-YFP foci normalized to the total cells analyzed.

#### Gene Ontology (GO) enrichment analysis

GO enrichment analyses were conducted using http://geneontology.org/ as described previously [22]. In short, this analysis employed a binomial test to assess whether specific gene groups in the experimental list were more or less enriched than expected in a reference group. The annotation dataset used for the analysis was the GO biological process complete. Gene lists analyzed for each condition (wt strain or ∆*dusB* complemented with various DusB variants) included genes with at least two-fold change in proteomics data for ∆*dusB* complemented strains versus wt, and an adjusted *P*-value < 0.05. Exhaustive lists of uploaded genes are indicated in Supplementary Table S3. The reference gene s*et al*l 3782 *V. cholerae* genes in the database. Annotation Version: PANTHER Overrepresentation Test (Released 20 220 712). GO Ontology database DOI: 10.5281/zenodo.6399963 Released 2022–03-22

#### Quantification and statistical analysis

For comparisons between 2 groups, first an F-test was performed in order to determine whether the variances are equal or different between comparisons. For comparisons with equal variance, Student’s t-test was used. For multiple comparisons, we used ANOVA. to determine the statistical differences (*P*-value) between groups. **** means *P *< 0.0001, *** means *P *< 0.001, ** means *P *< 0.01, * means *P *< 0.05. For survival tests, data were first log-transformed in order to achieve normal distribution, and statistical tests were performed on these log-transformed data. Means and geometric means for logarithmic values and standard errors are represented.

## Results

### Identification of VcDusB’s substrates and targeted dihydrouridylation site

**Figure 1.**
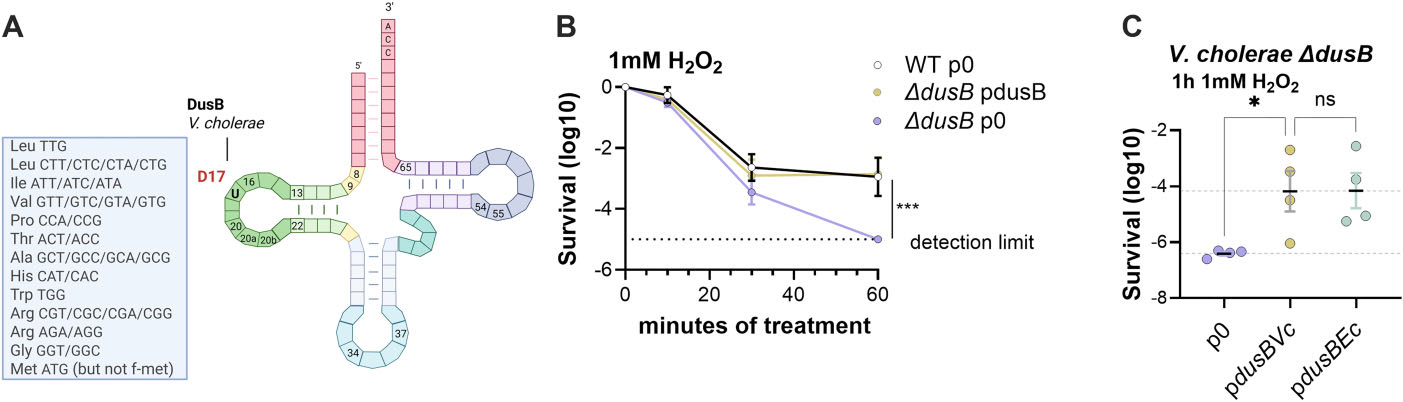
The *ΔdusB* genetic background is associated with a decreased survival rate to a lethal H_2_O_2_ treatment. (**A**). Summary of AlkAniline-sequencing analysis (detailed in Supplementary Fig. S2): *V. cholerae* tRNAs exhibiting D17 catalyzed by DusB. Created in BioRender. Baharoglu, Z. (2025) https://BioRender.com/154tnt5 (**B**). Log10 survival (*y*-axis) of exponential phase cultures after various incubation times (in minutes on the *x*-axis) with 1 mM H_2_O_2_. Strains were either complemented with a low copy pSC101 plasmid carrying *dusB* under the control of its native promoter (pdusB) or with the empty plasmid (p0). (**C**). Log10 survival to 1 h treatment with 1 mM H_2_O_2_ of *V. cholerae* Δ*dusB c*omplemented with various *dusB* variants (*V. cholerae* or *E. coli dusB*) or not (p0). The *y*-axis represents the log10 survival ratio. Means and standard errors are shown. For multiple comparisons, we used one-way ANOVA. ∗ means *P* < 0.05; ns means not significant. Number of biological replicates for each experiment: 3 ≤ *n* ≤ 4.

DusB is a redox enzyme whose physiological function remains unclear. In optimal growth conditions, its deletion causes no detectable fitness defect in *V. cholerae* [22]. To identify DusB-modified tRNAs in *Vibrio cholerae*, we employed AlkAnilineSeq [50], establishing a comprehensive list of tRNAs modified by DusB (Fig. 1A and Supplementary Fig. S1). The *V. cholerae* genome encodes 98 tRNAs, classified into 55 isodecoders that collectively decode 61 codons. Among these 55 isodecoders, 31 contain uridine at position 17 (U17). We found that, like *E. coli* DusB [43], VcDusB specifically modifies U17 in 26 tRNA isodecoders, which together decode 32 codons (Supplementary Table S1).

Using single mutants of the three *dus* genes, we demonstrated that VcDusA generates D20/D20a modifications, while VcDusC catalyzes D16 formation. These enzymes exhibit no dihydrouridylation redundancy, consistent with observations in *E. coli* (Supplementary Fig. S1).

### Deletion of *dusB* leads to H_2_O_2_ sensitivity in *V. cholerae*

Given that VcDusB modifies a large number of tRNAs without conferring any growth or fitness defect in rich media [22], we hypothesized that its role may be important under stress conditions. Because DusB is a flavoprotein involved in redox reactions, and *Vibrio cholerae* encounters oxidative stress during its life cycle in aquatic environments and upon infection, we investigated the contribution of *dusB* (VC_0291) to the bacterial antioxidant response. Deletion of *dusB* significantly impaired survival in the presence of 1 mM H_2_O_2_, and the native phenotype was restored by expressing *dusB* in trans (Fig. 1B). Complementation of *V. cholerae ∆dusB* with either *V. cholerae dusB* (VcDusB) or *E. coli dusB* (EcDusB) restored wild-type survival (Fig. 1C), showing that the two orthologs, which share 70% protein identity, are functionally interchangeable. Additionally, *V. cholerae* ∆*dusA* and ∆*dusC* single-knockout mutants also exhibited decreased survival in 1 mM H₂O₂, though to a lesser extent than the ∆*dusB* strain. Notably, the triple mutant showed a sensitivity comparable to that of the ∆*dusB* strain (Supplementary Fig. S2A), underscoring the critical role of dihydrouridine synthases, particularly DusB, in *V. cholerae*’s oxidative stress response. Digital RT-PCR revealed that *dusB* is expressed at substantially higher levels than *dusA* and *dusC* across growth phases in *V. cholerae* (Supplementary Fig. S2B), consistent with its predominant role in survival under oxidative stress. The ∆*dusB* strain also displayed increased sensitivity to other oxidative stressors, including menadione and diamide (Supplementary Fig. S2C), and the H₂O₂-sensitive phenotype was also conserved in *E. coli* K-12 strains MG1655 and BW25113, further highlighting its importance in bacterial oxidative stress defense (Supplementary Fig. S2D).

### Deletion of *dusB* impairs translation in a non-codon-specific manner

We next investigated whether *dusB* deletion affects translation and leads to codon-specific decoding defects in *V. cholerae*.

**Figure 2.**
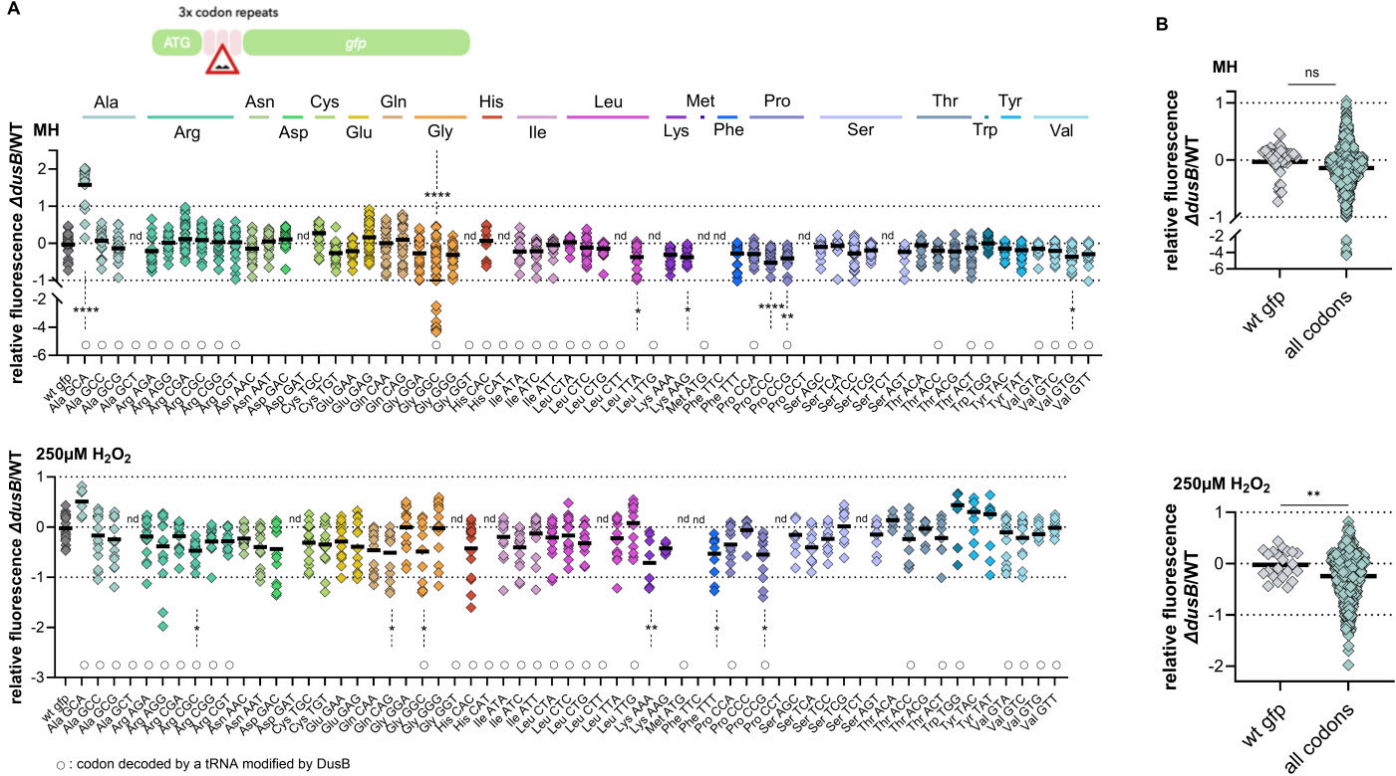
VcDusB influences codon decoding. (**A**). Codon-specific translation efficiency in ∆*dusB* relative to wild-type, in MH (non-treated, top panel) or upon exposure to low doses of H_2_O_2_ (250 µM H_2_O_2_, bottom panel). In this construct, three repeated identical codons (3x-codon stretches), functioning as a ribosome speedbump, were inserted within the *gfp* coding sequence immediately after the initiator codon. These stretches thereby amplify fluorescence difference arising from variations in codon decoding efficiency, providing a sensitive tool for translational dynamics assessment. The tested codons are listed on the *x*-axis, and the *y*-axis represents the mean values of log2-transformed fluorescence in *∆dusB* relative to WT, normalized to native *gfp* without codon repeats. Codons for which translation efficiency could not be assessed, because the inserted stretches abolished GFP fluorescence, are labeled as “nd” (not determined). (**B**). The fluorescence ratios obtained when all constructs with codon repeats are pooled, plotted on the graph, and compared with those from the native *gfp* reporter without repeats, to comprehensively assess the impact of *dusB* deletion on translation patterns, across all codons. For multiple comparisons, we used one-way ANOVA. For comparisons between two groups, an unpaired, one-tailed Student’s *t*-test was used. **** means *P *< 0.0001, *** means *P *< 0.001, ** means *P *< 0.01, * means *P *< 0.05. ns means not significant. Number of biological replicates for each experiment: 6 ≤ *n*, each dot represents one replicate.

Dual-reporter assays of stop codon readthrough and +1 ribosomal frameshifting (described in Supplementary Fig. S3AB) showed no impact of *dusB* deletion (Supplementary Fig. S3CD). However, ribosomal −1 frameshifting was notably reduced in the ∆*dusB* strain (Supplementary Fig. S3E). To assess codon-specific decoding efficiency, we used a set of 62 GFP reporters constructed as previously described [23]. Each GFP reporter contained a triplet of a specific codon immediately following the ATG start codon, directly challenging the translation machinery at that position (Fig. 2).

Our analysis revealed a widespread reduction in decoding efficiency across multiple codons in the absence of DusB, with one notable exception, the Ala-GCA codon. Under non-oxidative conditions (Fig. 2A, top panel), amino acid incorporation was significantly diminished for several codons decoded by DusB-modified tRNAs, including Gly-GGC, Pro-CCG, and Val-GTG. Unexpectedly, codons not associated with DusB-modified tRNAs, such as Leu-TTA, Lys-AAG, and Pro-CCC, also showed substantial reductions in decoding efficiency.

This trend was further exacerbated under oxidative stress (250 µM H₂O₂) (Fig. 2A, bottom panel), where both DusB-dependent codons (as with Arg-CGC, Gly-GGC, Pro-CCG) and non-substrates (e.g. Gln-CAG, Lys-AAA, Phe-TTT) suffered pronounced losses in decoding efficiency. Notably, Gly-GGC and Pro-CCG codons, decoded by DusB-modified tRNAs, were consistently compromised under both standard and oxidative conditions (Fig. 2B). These findings suggest that the impact of *dusB* deletion on codon decoding efficiency extends beyond its direct tRNA substrates, indicating a broader role for DusB in translation regulation.

While certain codon repeats exhibited minimal or no change in the absence of DusB, when examining the combined relative fluorescence ratios for all *gfp* reporters with codon repeat challenge (Fig. 2B: “all codons”), a notable overall reduction in translation efficiency was observed in ∆*dusB* relative to WT, particularly under oxidative pressure.

These results indicate that *dusB* deletion exerts a global effect on translation, impairing codon decoding across both DusB-modified and non-modified tRNAs, with especially pronounced effects under oxidative conditions.

### Rational design of DusB point mutants with targeted functional disruptions

**Figure 3.**
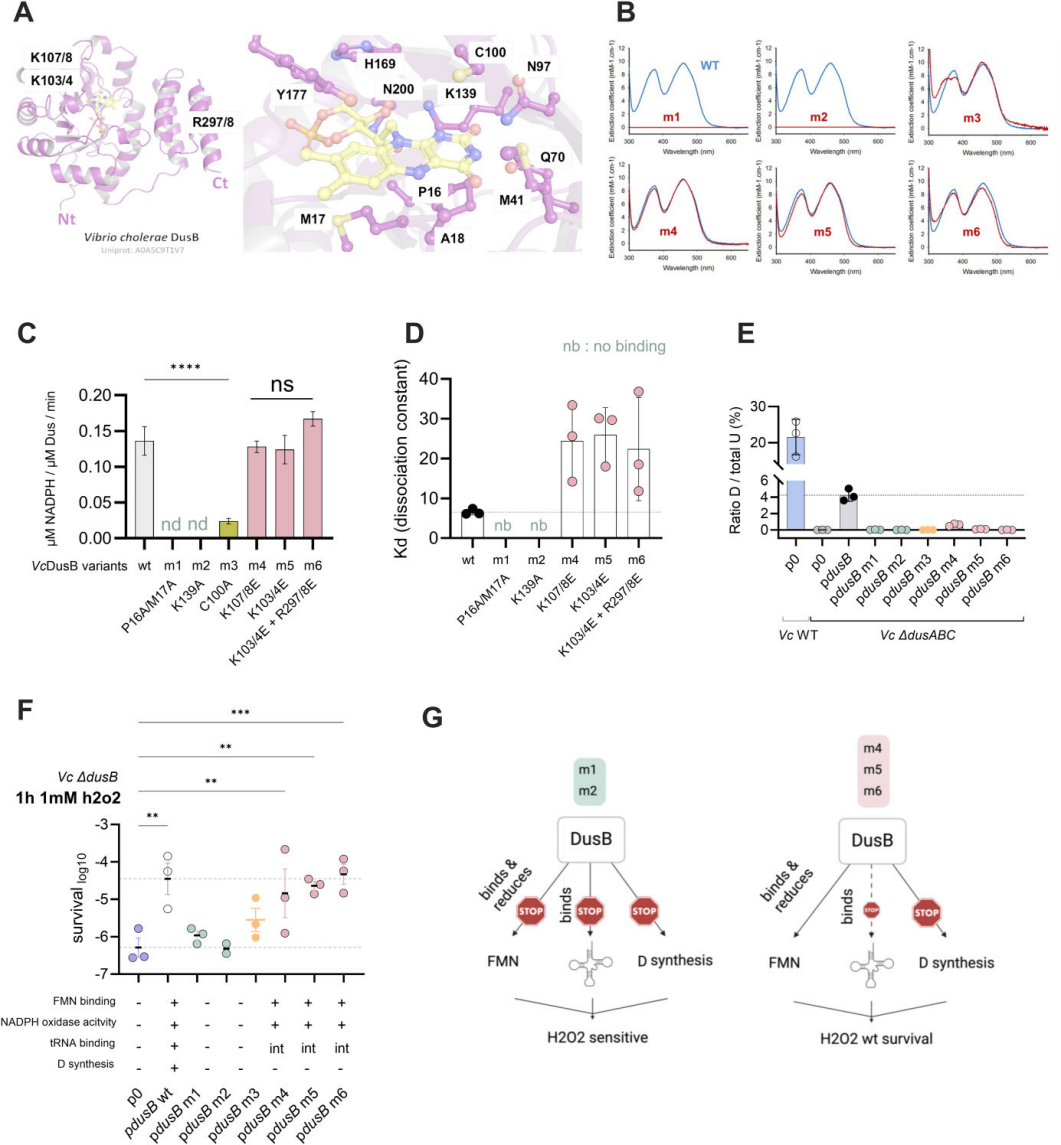
Functional characterization of DusB point mutants. (**A**) Alphafold model of VcDusB (left panel) with a detailed view of the FMN-binding pocket (right panel). Key amino acids are indicated. (**B**) *In vitro* assessment of DusB-FMN-binding by UV-visible absorption spectroscopy. The *y*-axis represents the extinction coefficient (in mM^−1^ x cm^−1^) while the *x*-axis shows the wavelength (in nm). The absorption profile is shown for each DusB variant and compared to wt. (**C**) *In vitro* measurement of NADPH oxidase activity (expressed as µM of NADPH consumed per µM of Dus per minute, shown on the *y*-axis). NADPH oxidase activity was not assessed for VcDusB m1 and m2 variants, as these do not bind FMN. (**D**) Characterization of interactions between tRNAs and VcDusB variants by electrophoretic mobility shift assay (EMSA). The EMSAs were performed three times, and Kd were calculated. (**E**) Quantification of D levels by mass spectrometry in tRNA-enriched RNA extracts from *Vc* ∆*dusABC* strains complemented with the DusB point mutants expressed from pSC101 low-copy plasmid. The *y*-axis represents the percentage of D relative to total U. (**F**) Survival to 1 h treatment with 1 mM H_2_O_2_ of *V. cholerae* ∆*dusB* complemented with pSC101 expressing either wild-type DusB or point mutants (or empty plasmid, p0). The *y*-axis displays the log10-transformed survival ratio. Assessment of protein properties is indicated beneath for each variant (int. for intermediate compared to wt). (**G**) Recapitulative scheme of DusB, indicating each mutant’s functional properties regarding FMN binding and reduction through NADPH oxidase activity, tRNA binding, and dihydrouridine (D) incorporation into tRNAs. STOP indicates a complete loss of function, dashed lines represent intermediate activity relative to the wt. Below each diagram, the capacity of the variant to restore survival of *∆dusB* under 1 h 1 mM H_2_O_2_ treatment is noted. Mean values are represented. For multiple comparisons, we used one-way ANOVA. **** means *P *< 0.0001, *** means *P *< 0.001, ** means *P *< 0.01, * means *P *< 0.05. ns means not significant. Number of replicates for each experiment: 3 ≤ *n*, each dot represents one replicate.

Dus proteins exhibit two distinct functional properties: (i) they bind their FMN cofactor, which is required for NADPH oxidation and U17 reduction in substrate tRNAs; (ii) they possess a positively charged electrostatic surface, formed by basic amino acid patches, that mediate tRNA binding. Both properties are strictly required for tRNA dihydrouridylation activity [40, 41]. To selectively disrupt these two distinct functional features of DusB, we employed a structure-guided approach. Using a structural model of the VcDusB holoenzyme (Fig. 3A), we identified key residues involved in each function. FMN was docked into the apoprotein model generated by AlphaFold, which was aligned with the crystallographic structure of EcDusB. This model is highly reliable due to the strong sequence similarity between the DusB proteins of the two species.

We designed six mutants: (i) m1 (P16A/M17A) and m2 (K139A), which aim to destabilize FMN binding; (ii) m3 (C100A) catalytic site mutant; and (iii) m4 (K107E/K108E), m5 (K103E/K104E), and m6 (K103E/K104E/R297E/R298E), which destabilize the tRNA binding site. This strategy offers a precise means to dissect the impact of each individual function on DusB’s overall activity, providing insights into its roles in *V. cholerae* biology.

### Biochemical characterizations of VcDusB inactive mutants

Our biochemical analyses revealed distinct functional impairments among the DusB mutants. Wild-type VcDusB and mutants m3, m4, m5, and m6 copurified with their FMN coenzyme, as indicated by their characteristic absorbance spectra (Fig. 3B), featuring the S₀–S₂ band at 372 nm and the S₀–S₁ band around 450 nm. Upon the addition of sodium dodecyl sulfate (SDS), the proteins were denatured, releasing flavin into the solution. The resulting flavin displayed an absorption spectrum similar to that of free FMN, confirming that both the wild-type VcDusB and mutants (m4, m5, m6) are flavoproteins with FMN non-covalently bound to the apoprotein. As expected, mutants m1 and m2, which were designed to disrupt FMN binding, did not copurify with flavin, confirming the loss of FMN binding in these mutants (Fig. 3B). Native DusB exhibited NADPH oxidase activity of 0.136 ± 0.02 µM of NADPH/µM Dus/min (Fig. 3C). The NADPH oxidase activity of the FMN-binding proficient mutants (m4, m5, and m6) was comparable, with activities of 0.128 ± 0.008, 0.124 ± 0.02, and 0.167 ± 0.01 µM of NADPH/µM Dus/min, respectively. For m3, the NADPH oxidase activity was drastically reduced (0.024 ± 0.04 μM NADPH/μM Dus/min for wild-type, suggesting a defect in the redox function associated with this mutant. Mutants m1 and m2 were excluded from this analysis due to their inability to bind FMN, as they could therefore not oxidize NADPH.

An electrophoretic mobility shift assay (EMSA) was conducted to assess the tRNA-binding efficiency of DusB variants. Surprisingly, both mutants m1 and m2 showed no detectable DusB-tRNA complexes, even after incubation with high protein concentrations (50 µM), indicating that tRNA binding was completely abolished in these mutants (Fig. 3D and Supplementary Fig. S4). Notably, the mutated residues in these variants were not expected to directly impact tRNA binding but were thought to affect FMN recognition. These results suggest that FMN binding may be critical for tRNA binding. In contrast, mutants m4, m5, and m6, which were designed to target residues likely involved in tRNA binding, exhibited a four-fold decrease in the formation of DusB-tRNA complexes compared to the wild-type protein (Fig. 3D). m3 consistently copurified with bound tRNA, suggesting a defect in tRNA dissociation. EMSA (Supplementary Fig. S4) further showed that m3 retains partial tRNA-binding capacity, consistent with impaired dissociation dynamics compared to wild-type DusB.

Finally, mass spectrometry analysis of tRNA-enriched RNA extracts from *ΔdusABC* strains complemented with DusB variants confirmed that all mutants are deficient in D-modification, with D levels being nearly undetectable (Fig. 3E). Only the m4 variant showed a minor level of D modification (0.78% D compared to 4.22% with the wild-type DusB). The ectopic expression levels of each variant were verified and found to be comparable to those of wild-type DusB, whether expressed ectopically or chromosomally (Supplementary Fig. S5). Note that the catalytic mutant (m3), which inactivates cysteine at position 100, proved toxic and accumulated mutations inactivating the promoter region during growth (Supplementary Fig. S2E).

### Oxidative stress survival requires DusB but not tRNA dihydrouridylation

We next assessed the ability of DusB variants to restore survival in *ΔdusB* strains exposed to lethal concentrations of H₂O₂ (Fig. 3F). Despite partial NADPH oxidase activity and residual tRNA binding, the Cys100Ala variant lacking D-synthesis capacity only partially restored H₂O₂ survival. Surprisingly, mutants m4, m5, and m6, which retained functional FMN binding and NADPH oxidase activity but exhibited decreased tRNA binding capabilities and lacked D-synthesis capacities, were able to fully restore H₂O₂ survival. Notably, the D17-deficient mutants were unable to reverse the impact of the *dusB* deletion on ribosomal frameshifting (Supplementary Fig. S3E), highlighting the essential role of D17 modification in maintaining ribosomal frame accuracy.

In summary, none of the mutants were capable of catalyzing D17 modification. Mutants m1 and m2 neither bind tRNA nor FMN and cannot restore H₂O₂ survival. In contrast, mutants m4, m5, and m6, despite being impaired in tRNA binding, retained NADPH oxidase activity and were able to restore H₂O₂ survival.

These results collectively highlight that DusB’s involvement in H_2_O_2_ response is independent of its ability to catalyze U to D reduction at position 17 on tRNAs. Instead, it relies on other function (s), particularly its NADPH oxidase activity and possibly its tRNA binding activity. A summary of the properties and phenotypes of the DusB variants is illustrated in Fig. 3G.

### The absence of DusB negatively impacts the proteome but not the transcriptome, in the presence of H_2_O_2_

**Figure 4.**
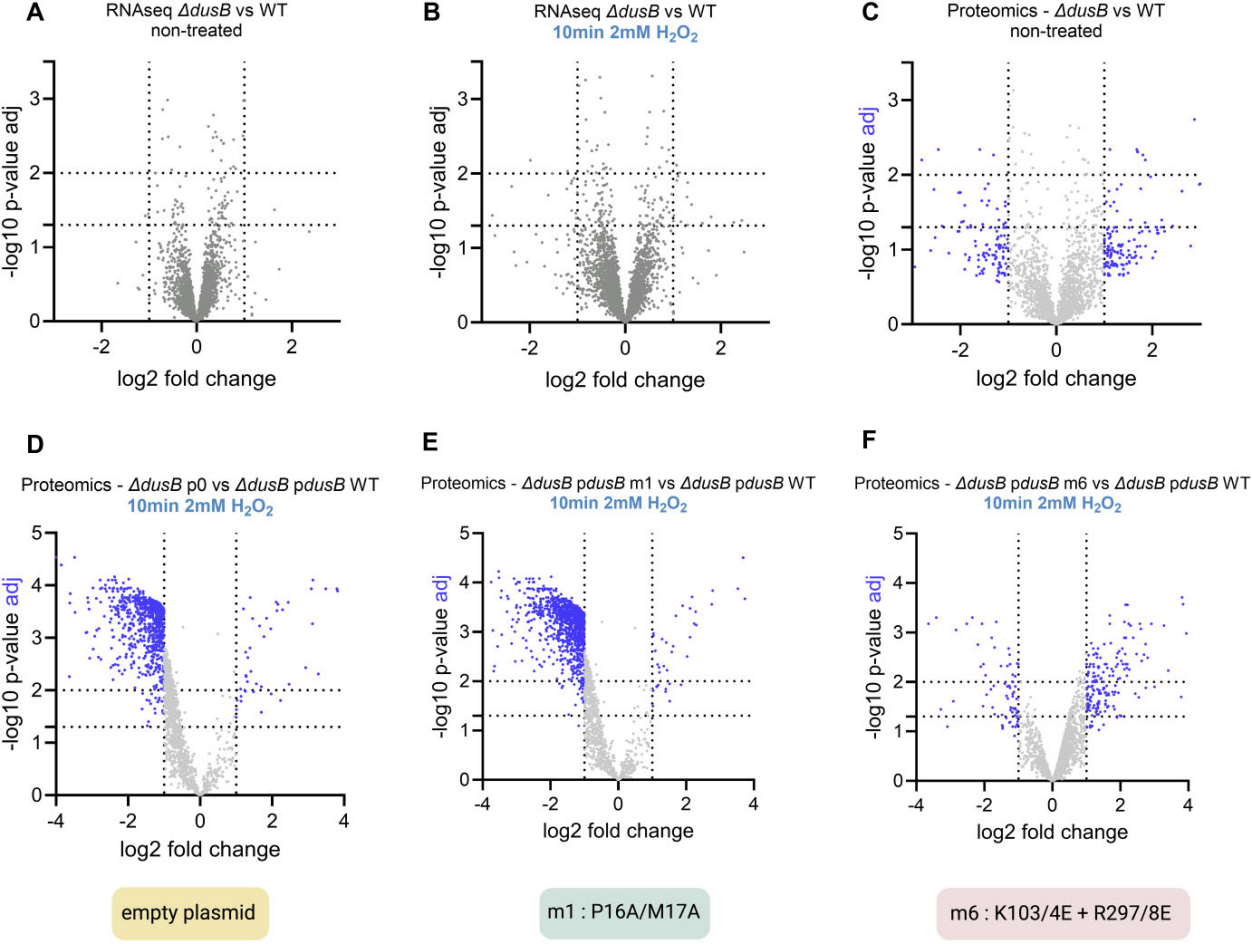
Comparative transcriptome and proteome analysis of ∆*dusB* and wt. (AB). Volcano plots showing differentially abundant transcripts from RNA-seq analysis in ∆*dusB* compared to WT (**A**) in the absence of stress or (**B**) after 10 min exposure to 1 mM H_2_O_2_. CDEF. Volcano plots displaying differentially expressed proteins between ∆*dusB* and WT (**C**) under optimal growth conditions and various complemented ∆*dusB* strains, following 10 min treatment with 1 mM H_2_O_2_ (**D**) ∆*dusB* with an empty plasmid versus complemented with native *dusB*, (**E**): ∆*dusB* complemented with the *dusB* m1 variant versus native *dusB*, and (**F**) ∆*dusB* complemented with the *dusB* m6 variant versus native *dusB*. For proteomics (CDEF), the *x*-axis represents the log₂ fold change, and the *y*-axis shows the –log₁₀ *P*-value (in grey) and the –log₁₀ adjusted *P*-value (in blue). Adjusted *P*-values were calculated only for proteins with statistically significant differences (adjusted *P*-value < threshold). All experiments were conducted at least in biological triplicates.

To investigate the impact of *dusB* deletion on transcriptome and proteome profiles under both non-stress and oxidative conditions, we performed RNA-seq and proteomics analyses. RNA-seq results revealed that the *∆dusB* transcriptome closely resembles the wild-type under both conditions (Fig. 4A and B). Only 10 protein-coding transcripts were differentially regulated significantly under non-stressed conditions, and 20 showed changes by more than two-fold under H₂O₂ stress (Supplementary Table S2). Among these, some were related to infection and phage defense (in bold in Supplementary Table S2). Proteomics analysis also revealed similar proteome profiles between the two strains under non-stress conditions, with 93 proteins exhibiting differential abundance. Nevertheless, there was no significant enrichment of specific gene categories or pathways (Fig. 4C, Supplementary Table S2).

However, after exposure to high levels of H₂O₂, we observed a significant leftward shift in the proteomic data, with 980 proteins showing at least a two-fold decrease in abundance and 84 proteins upregulated in *∆dusB* compared to the wild-type (Fig. 4D, Supplementary Table S3). This indicates a substantial reduction in protein levels in *∆dusB* under oxidative stress, which aligns with the GFP-reporter assay results described earlier. This trend was consistent when comparing *∆dusB* complemented with the m1 variant (which lacks FMN binding, NADPH oxidase activity, tRNA binding, and D synthase functions) to the strain complemented with native DusB (1239 proteins downregulated, 62 upregulated) (Fig. 4E, Supplementary Table S3).

In contrast, the proteome of *∆dusB* complemented with the m6 variant, which lacks only the dihydrouridine synthase activity but retains other DusB functions and effectively rescues the *∆dusB* survival defect under H₂O₂ stress, closely resembles that of the strain complemented with native DusB (Fig. 4F). Here, only 319 proteins were differentially regulated, with 124 showing downregulation (Supplementary Table S3). These findings suggest that DusB’s NADPH oxidase activities and partial tRNA binding properties are essential for maintaining proper protein levels during oxidative stress, which is crucial for survival under these conditions.

We asked whether altered tRNA stability or degradation could explain the reduced protein abundance in the *∆dusB* strain. Northern blot analyses of three representative tRNA species (both DusB-modified and unmodified, Supplementary Fig. S5A) revealed no significant differences in degradation rates between *∆dusB* and wild type (Supplementary Fig. S5B). Direct RNA tRNA-seq likewise showed only minimal changes in abundance, with slight increases in tRNA-Ala (GCA, log2FC = +0.26) and tRNA-Ile (ATC, log2FC = +0.5), and no differences between the *∆dusB* and *dusBm6* strains (Supplementary Fig. S5C). Thus, the global proteomic effects of DusB deletion cannot be explained by altered tRNA stability or abundance.

Proteomics analysis comparing the DusB m6 mutant with wild-type DusB and *∆dusB* allowed us to identify proteins that are restored upon expression of a DusB variant deficient in D17 modification but retaining the NADPH oxidase activities and 10% tRNA binding of DusB. We performed gene ontology (GO) enrichment analysis on the differentially abundant proteins (with an adjusted *P*-value < 0.05) (Supplementary Fig. S6).

The abundance of proteins involved in NADPH metabolism, particularly those involved in NADPH regeneration (Pgl, RpiA, Tal, Tkt1, Tkt2), is disrupted in Δ*dusB* (Supplementary Fig. S6, Supplementary Table S3). However, this disruption is not observed in the DusB m6 variant, suggesting that this regulation of NADPH metabolism is independent of D17 modification. pHrodo, a pH-sensitive fluorescent dye whose intensity increases with acidification, was employed to monitor changes in the cytoplasmic pH of bacterial cells. Acidification of the cytoplasm in *∆dusB* is also consistent with dysregulation of NADPH metabolism (Supplementary Fig. S7A).

Additionally, under oxidative stress, gene products associated with protein folding, including various chaperones and proteins with chaperone-related functions (ClpB, DnaJ, DnaK, GrpE, HscA, HslO, HtpG), as well as enzymes involved in the quality control of misfolded proteins (for instance Lon, Protease DO encoded by VC_0566), were found to be deregulated in the *∆dusB* strain compared to wild-type, but not in the *∆dusB pdusBm6* strain (Supplementary Fig. S5, Supplementary Table S3). This deregulation is consistent with the severe translational defect observed in *∆dusB* following H₂O₂ treatment. Moreover, deletion of *dusB* led to increased accumulation of IbpA-YFP foci, indicative of protein aggregation [73] (Supplementary Fig. S7B). Strikingly, aggregation in *∆dusB* in the absence of stress reached levels comparable to those observed in cells exposed to lethal H₂O₂ concentrations, suggesting a profound disruption in protein homeostasis.

Similarly, RNA processing proteins are deregulated in Δ*dusB*, independent of the lack of D17 modification. Notably, this includes the deregulation of several tRNA-modifying enzymes (like MiaA, MiaB, MnmA, TrmB, TruB, TrmA) and various enzymes involved in tRNA-aminoacylation (e.g. AlaS, AsnS, EpmA, GlnS, GltX, GluQ, GlyQ, GlyS, HisS, LeuS, LysS, MetG, ValS).

On the other hand, comparative proteomics analysis of the m6 variant versus wild-type also allowed for the identification of proteins whose regulation is impacted by the absence of D17 modification (Supplementary Fig. S8, Supplementary Table S3). The absence of D17 results in the dysregulation of proteins involved in nitrogen metabolism, motility, signaling, as well as carbohydrate, vitamin (CobQ), and iron metabolism (the iron-regulated protein A encoded by VC_1266, HmuV). Unlike wild-type DusB, neither DusB m1 nor DusB m6 can restore the native abundance levels of these proteins. Additionally, we observed alterations in the abundances of proteins related to sugar utilization (e.g. MalG) and proteins involved in host colonization, biofilm formation, and virulence (TcpA, VspR).

The pronounced proteomic defects in *ΔdusB* likely arise from cumulative reductions in codon decoding efficiency under oxidative stress, which disrupt global translation and promote misfolding, together with secondary post-translational effects driven by redox imbalance and altered proteostasis.

### TN-seq reveals interplay between DusB, oxidative stress pathways, and cell metabolism

**Figure 5.**
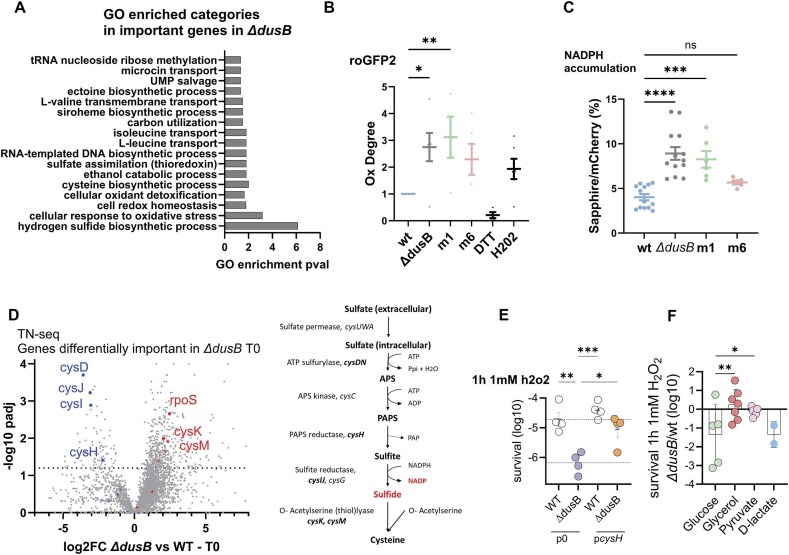
TN-seq highlights the role of DusB in oxidative stress pathways and cell metabolism. (**A**). GO analysis for TN-seq data. The reference gene list was *V. cholerae* (all genes in the database), 3782 genes. (**B**). roGFP2-based measurement of oxidation degree (OxD) in wild-type, *ΔdusB*, and mutant strains. Around 10 mM DTT and 1 mM H202 were used as reduced and oxidized controls, respectively (10 min treatment). (**C**). NADPH biosensor analysis revealed accumulation in the *dusB* deletion and redox-deficient *dusBm1* strains. (**D**). Volcano plot shows genes for which the number of transposons inactivation is increased (beneficial) or decreased (detrimental) at T0 in *∆dusB* compared to wt, and representation of the cysteine biosynthesis pathway. Genes whose inactivation by transposon insertion has a significantly different impact in *∆dusB* compared to WT are indicated in bold. (**E**). Survival to 1 h treatment with 1 mM H_2_O_2_ of *V. cholerae* wt and ∆*dusB* complemented with pSC101 expressing *cysH* gene under the *gyrA* promoter (or empty plasmid, p0). The *y*-axis displays the log10-transformed survival rate. (**F**). Log₁₀-transformed survival ratio of the *∆dusB* mutant relative to the wild-type after 1 h of exposure to 1 mM H₂O₂ in the presence of the indicated sugars.

To gain insight into how the redox properties of DusB contribute to the response to oxidative stress, we performed transposon insertion sequencing (Tn-seq) on the *∆dusB* strain. We analyzed the initial library (T0) and after 16 generations of growth (T16), as previously described [22] (Supplementary Table S4). At T0, gene ontology enrichment analysis revealed that inactivation of genes in the *∆dusB* strain, compared to the wild-type, was most significantly associated with categories related to “hydrogen sulfide biosynthetic process,” oxidative stress, and redox reactions (Fig. 5A). The results at T16 further highlighted the importance of oxidoreductase activity, especially enzymes acting on NAD(P)H, quinone, or similar compounds as acceptors (*nqrABEFD*), as well as thiamine and riboflavin biosynthesis (*ribABCD*). These findings underscore DusB’s major role in the oxidative stress response and suggest its involvement in regulating the NADP/NADPH balance. Using roGFP2, a genetically encoded redox-sensitive fluorescent protein that reports intracellular H₂O₂ levels via ratiometric fluorescence changes [48], we observed that *ΔdusB* and *dusBm1* mutant cells show increased steady-state H₂O₂ concentrations relative to wild-type (Fig. 5B). To monitor intracellular NADPH levels, we adapted a recently developed fluorescent biosensor [49] for expression in *V. cholerae* by codon optimization, and using this tool, we detected significant NADPH accumulation in the *dusB* deletion mutant as well as in the redox-deficient *dusBm1* strain (Fig. 5C).

Strikingly, the Tn-seq results revealed differential inactivation patterns for genes in the cysteine biosynthesis pathway (Fig. 5D). Specifically, inactivation of the *cysD, cysI, cysJ*, and *cysH* genes became detrimental to the *∆dusB* strain, while inactivation of *cysK* and *cysM* proved beneficial. These genes are involved in different steps of cysteine biosynthesis. CysDIJH catalyzes reactions from sulfate to sulfide production (Fig. 5D), while CysKM is responsible for the final step, converting sulfide into cysteine. Inactivation of CysKM would lead to the accumulation of sulfide and NADP+, whereas inactivation of CysDIJH would prevent sulfide and NADP+ accumulation.

Additionally, proteome data showed a significant decrease in CysH levels in the DusBm1 mutant, but not in DusBm6, compared to the wild-type DusB under H₂O₂ stress (Supplementary Table S3). Based on this observation, we hypothesized that NADP production via the cysteine (*cys*) biosynthesis pathway might be important for the *∆dusB* strain’s response to oxidative stress. To test this, we overexpressed CysH in the *∆dusB* strain and observed restoration of H₂O₂ survival (Fig. 5E). This result supports the idea that NADP production through the *cys* pathway plays a key role in the mutant’s ability to withstand oxidative stress.

On the other hand, the pentose phosphate pathway (PPP), a major source of NADPH, was implicated in this process. Sugar substrate choice strongly affected PPP flux. Pyruvate enters the TCA cycle downstream of the PPP, reducing NADPH production; glycerol likewise feeds directly into glycolysis, bypassing the PPP. In contrast, glucose is converted to glucose-6-phosphate, driving the oxidative PPP and generating NADPH. Lactate differs: it is converted to pyruvate by lactate dehydrogenase in a reaction that increases NADH/NAD⁺ but does not lower PPP-derived NADPH. We observed that *∆dusB* was resistant when grown on glycerol or pyruvate, but remained sensitive with glucose or lactate (Fig. 5F). This suggests that excess NADPH is harmful in the *∆dusB* background, and that shifting the balance toward NADP⁺, rather than NADPH, may provide protection against oxidative stress.

Finally, we tested whether heterologous expression of several NADPH-dependent flavoenzymes could rescue the H₂O₂ survival defect of the *∆dusB* strain, but none were effective (Supplementary Fig. S7C). This indicates that the effect of DusB on NADPH pools is specific and linked to the cysteine biosynthesis pathway, rather than a general property of flavoproteins.

In conclusion, these results indicate a metabolic shift in the *∆dusB* strain, where the NADP/NADPH equilibrium is altered towards NADPH. This shift is also consistent with the observed acidification of the *∆dusB* strain’s cytoplasm (Supplementary Fig. S7A), further supporting the involvement of metabolic changes in the oxidative stress response.

### Conservation across VcNN strains of catalytically inactive DusB variants underscores the evolutionary significance of these additional functions

**Figure 6.**
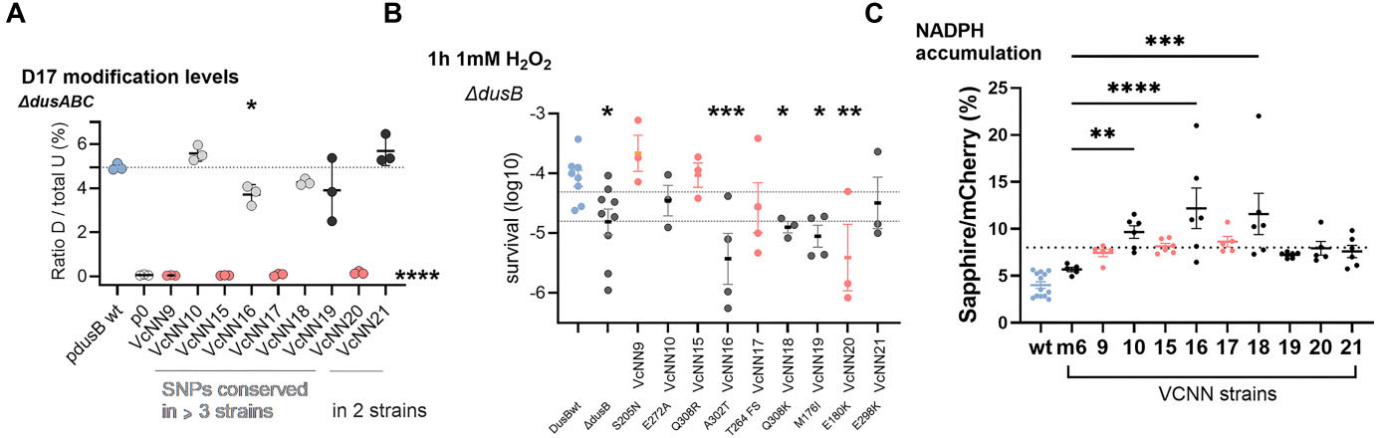
Phenotypic characterization of VCNN DusB variants. (**A**) Quantification of D levels by mass spectrometry in tRNA-enriched RNA extracts from *Vc*Δ*dusABC* strains complemented with the VcNN’s dusB variants expressed from pSC101 low-copy plasmid. The *y*-axis represents the percentage of D relative to total U. Statistical significance tests were performed comparing the indicated strain to pDusBwt. (**B**) H₂O₂ survival assays of the indicated strains. *∆dusB* with plasmid expression DusBwt or empty plasmid is shown as a control. Statistical significance tests were performed comparing the indicated strain to pDusBwt. (**C**). NADPH accumulation was measured using the fluorescent biosensor. Statistical significance tests were performed comparing the indicated strain to pDusBm6. (ABC). For multiple comparisons, we used one-way ANOVA. **** means *P *< 0.0001, *** means *P *< 0.001, ** means *P *< 0.01, * means *P *< 0.05. Only significant differences are shown. Number of biological replicates for each experiment: 3 ≤ *n*, each dot represents one replicate.

We performed a bioinformatics analysis of the *dusB* gene sequences across various strains from the National Reference Center for Vibrio and cholera collections [74, 75], using the *V. cholerae* O1 El Tor strain N16961 as the reference sequence. Among 1200 pandemic *V. cholerae* O1 El Tor strains (7PET lineage) from different sublineages, all *dusB* sequences were found to be 100% identical to the reference, including strains isolated more than 50 years ago. In contrast, within 171 *V. cholerae* O1 classical strains isolated between 1888 and 2000, we identified 7 single-nucleotide polymorphisms (SNPs), which were consistent across all strains. Meanwhile, among 313 *V. cholerae* non-O1/non-O139 (VCNN) non-pandemic strains from environmental reservoirs, up to 51 SNPs were observed in the *dusB* gene. These results highlight the remarkable sequence conservation of *dusB* in strains isolated from infected patients, while showing increased sequence diversity in non-infectious/environmental strains. Among the identified nonsynonymous SNPs in *VcNN* strains, which were selected for their occurrence in at least two distinct strains and integrated into *VcDusB*, four (representing almost half of the selected variants) were associated with the loss of dihydrouridylation activity (Fig. 6A).

To characterize the phenotypes associated with the VCNN DusB variants, we examined (1) H₂O₂ sensitivity (Fig. 6B) and (2) NADPH accumulation in these strains (Fig. 6C). Three variants restored H₂O₂ survival of the *ΔdusB* strain (VCNN9, VCNN10, and VCNN15), four remained sensitive (VCNN16, VCNN18, VCNN19, and VCNN20), and two showed variable responses (VCNN17 and VCNN21). Among the “no-D” variants, none of which accumulate NADPH, VCNN9 and VCNN15 resembled DusBm6 in restoring H₂O₂ survival. In contrast, VCNN17 and VCNN20 displayed H₂O₂ sensitivity. Notably, the VcNN9 variant, which lacks catalytic activity, fully rescued H₂O₂ sensitivity, confirming that DusB’s role in oxidative stress resistance is independent of D17 modification. In contrast, the VcNN10 variant, which retains native enzymatic activity, only partially rescued survival, suggesting a possible alteration in its redox-related function, potentially through changes in molecular interactions unrelated to tRNA modification. Thus, H₂O₂ sensitivity and NADPH accumulation phenotypes did not necessarily correlate, but the results confirm that D17 modification by DusB is not required for H₂O₂ survival. Overall, these findings suggest that environmental variants can give rise to diverse tRNA modification and redox phenotypes, and underscore the evolutionary significance of the newly characterized functions of DusB.

## Discussion

Our study fundamentally redefines the functional scope of tRNA-modifying enzymes by demonstrating that VcDusB plays an essential, non-canonical role in oxidative stress defense through its NADPH oxidase activity independent of its tRNA modification function. This discovery challenges the traditional paradigm of Dus enzymes being solely dedicated to dihydrouridine biosynthesis and reveals an unexpected integration of RNA metabolism with cellular redox homeostasis.

The genetic and biochemical dissection of DusB’s activities yielded several key insights. First, while DusB’s tRNA dihydrouridylation influences translation efficiency and fidelity (frameshift), these activities are dispensable for oxidative stress resistance. Instead, our complementation studies with mutants affecting different functionalities of the enzyme clearly demonstrate that NADPH oxidation mediated by the FMN coenzyme is the critical determinant of H_2_O_2_ tolerance. The inability to completely uncouple tRNA binding from NADPH oxidase activity suggests these functions may be structurally or mechanistically intertwined, possibly through conformational coupling between the FMN-binding and tRNA-interaction domains.

Although NADPH is generally protective, its overaccumulation can paradoxically trigger oxidative stress, a condition known as reductive stress. When the NADPH/NADP⁺ ratio is highly elevated, reduced flavoproteins can leak electrons to oxygen, producing ROS [76, 77]. Such overflow has been reported in both bacteria and mammalian cells. We propose that in the *∆dusB* and *dusBm1/m2* mutants, loss of DusB-dependent NADPH oxidase activity causes NADPH buildup, which can fuel ROS formation and underlie their hypersensitivity to oxidative stress.

Proteomics results, using the DusB variant which retains NADPH oxidase activity and with reduced tRNA binding but completely lacking D17 modification, uncover DusB’s influence on the regulation of proteins involved in NADPH metabolism, RNA processing, and protein folding, critical processes for maintaining cellular redox balance (Supplementary Fig. S6 in red). Importantly, these effects occur in the absence of substantial transcriptomic changes, indicating post-transcriptional control likely mediated by redox-sensitive processes. The proteomic changes observed upon *dusB* deletion are likely multifactorial. Codon decoding defects under oxidative stress may contribute to ribosome elongation problems such as stalling and collisions, while also impacting protein folding, stability, degradation, and oxidation. Furthermore, the imbalanced redox state of the *∆dusB* strain likely exacerbates these effects.

Tn-seq data further suggest that DusB is integrated into metabolic networks controlling sulfur assimilation and potentially NADP+/NADPH balance. The genetic interactions with cysteine biosynthesis genes and the rescue of stress sensitivity by CysH overexpression point to a compensatory relationship between DusB-mediated redox activity and central redox metabolism. This connection is further supported by the ability of specific carbon sources to modulate DusB-dependent phenotypes, reinforcing the idea that DusB helps fine-tune cellular redox potential in response to environmental cues. However, heterologous expression of several other flavoproteins in the *∆dusB* strain did not rescue H₂O₂ survival, suggesting that DusB’s effect on NADPH pools is specific rather than general and is linked to the cysteine biosynthesis pathway. Further work will be required to disentangle these mechanisms.

Bioinformatic analysis of over 1600 *Vibrio cholerae* strains revealed that the dusB sequence is strictly conserved across all cholera-causing clinical strains, underscoring the evolutionary pressure to preserve its full functionality in pathogenic strains. This conservation is consistent with the observation that processes seemingly dependent on D17 modification, as indicated in our proteomics data, include motility, biofilm formation, and virulence-related factors. These findings suggest that tRNA D17 modification is crucial for the *V. cholerae* infectious cycle and host colonization.

In contrast, *V. cholerae* non-O1/non-O139 (VCNN) strains, which are primarily environmental isolates not linked to pandemics, display multiple SNPs in the *dusB* gene. This prompts further exploration into how these variants may alter DusB’s functionality, particularly in terms of tRNA binding, NADPH oxidase activity, and D17 modification. An intriguing hypothesis is that loss of D17 may confer an adaptive advantage, reflecting a trade-off between D-proficient and no-D DusB variants. Future studies will be needed to test this possibility.

Modification enzymes acting on the T-arm have been shown to moonlight as chaperones that facilitate aminoacylation, and Dus enzymes targeting the D-arm could potentially act in a similar manner. Although no such data are available for DusB, a recent preprint [78] on DusA, which reduces U to D at position 20, reports that DusA does not exert a general effect on tRNA abundance or aminoacylation but instead modulates the charging of specific tRNAs and improves decoding of specific codons. Whether DusB functions as a chaperone, influences aminoacylation, or alters tRNA structure remains an open question.

Interestingly, similar dual activities have been recently described, for example, the protective role of NADPH-dependent mechanisms against oxidative stress in the human tRNA ligase complex, namely PYROXD1-RTCB complex [79] has recently been demonstrated, underscoring its importance in maintaining tRNA ligase activity under aerobic conditions. In addition, redox metabolic regulation may involve other RNA modifications like m^6^A, as NADP directly activates FTO to enhance m^6^A demethylation, revealing a metabolic-RNA feedback loop, linking NADP to FTO-mediated gene regulation [80]. These insights align with the broader theme of cofactor-driven redox control in bacterial and eukaryotic systems, emphasizing the evolutionary conservation of NADPH-mediated protection.

The broader implications of our findings are threefold. First, they establish that tRNA-modifying enzymes can function as metabolic sensors, directly linking RNA biology to cellular physiology. Second, they suggest that the redox activity of flavin-dependent RNA modification enzymes may represent an underappreciated layer of stress regulation across biology. Finally, the conservation of DusB in bacteria implies that this mechanism could extend beyond Vibrio species. The discovery of DusB’s dual functionality- both as a tRNA-modifying enzyme and a redox regulator- paves the way for future research aimed at uncovering the precise molecular mechanisms behind these activities. By doing so, we open new avenues for understanding how tRNA-modifying enzymes integrate into cellular defense strategies, particularly in response to environmental challenges like oxidative stress.

## Supplementary Material

gkaf1276_Supplemental_File

## Data Availability

Raw AlkAnilineSeq data are available at the European Nucleotide Archive (https://www.ebi.ac.uk/ena/browser/home) under the accession number PRJEB88079. Data for RNA-seq are available at the GenBank repository under the project number GSE288937. All mass spectrometry proteomics data have been deposited at ProteomeXchange Consortium via the PRIDE partner repository with the dataset identifiers PXD057105, PXD057145, and PXD057061.
