# Supplementary material for: Beyond RNA modification: a novel role for tRNA modifying enzyme in oxidative stress response and metabolism
Source: Nucleic Acids Res. 2025 Dec 12;53(22):gkaf1276. doi: 10.1093/nar/gkaf1276 (PMC12700106; doi:10.1093/nar/gkaf1276)
Supplement: gkaf1276_Supplemental_File [file gkaf1276_supplemental_file.pdf]

Supplementary figures

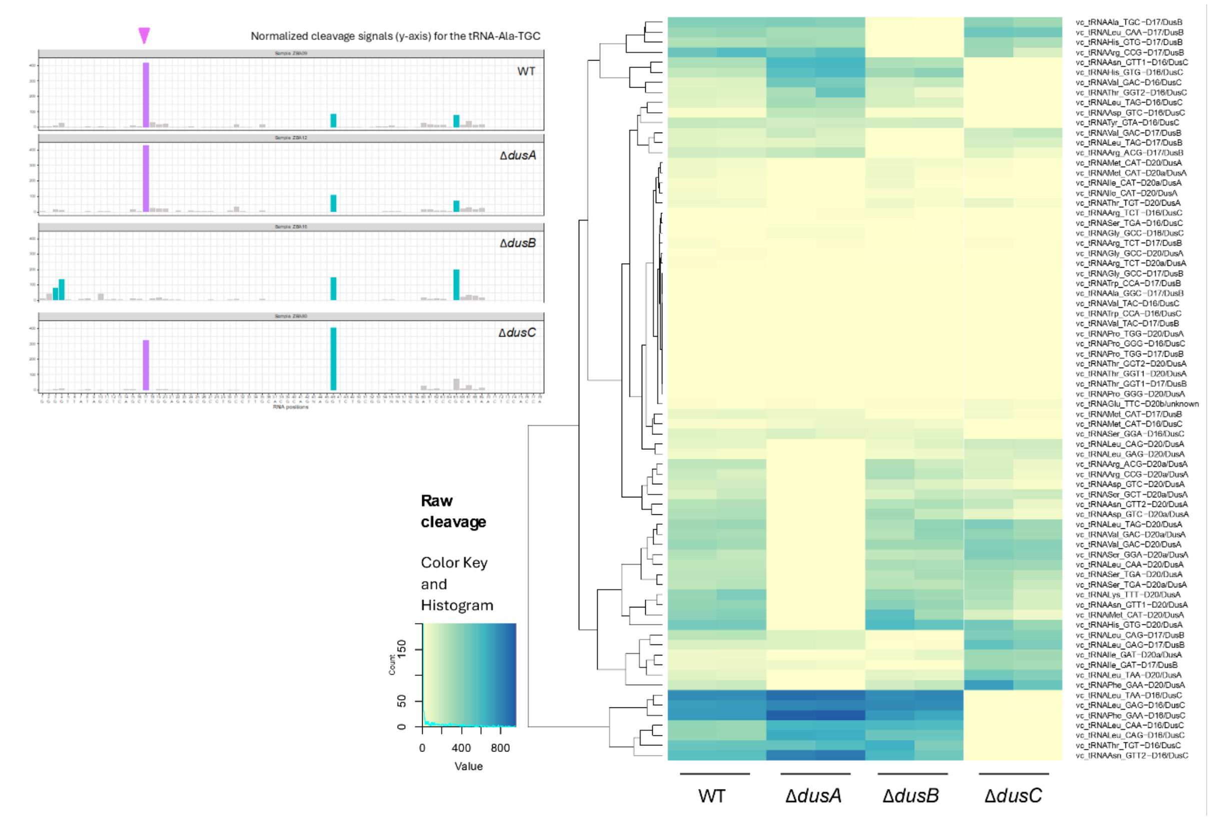

Figure S1: AlkAniline-Sequencing analysis in *dus* knock-out mutants. Left panel. Normalized Ncleavage signals (y-axis) for the tRNA-Ala-TGC in tRNAs-enriched RNA fractions extracted from *V. cholerae* wt, Δ*dusA,* Δ*dusB and* Δ*dusC* strains. Nucleotide identities and positions are indicated on the x-axis. Signal corresponding to D17 is highlighted by a pink arrow (top panel). AlkAniline-seq was conducted on 3 independent replicates, all of which gave consistent results. Only one replicate is presented. Similar profiles were observed for tRNAs: Ala-GGC-1, Ala-TGC-1, Ala-TGC-2, Arg-ACG-1, Arg-ACG-2, Arg-ACG-3, Arg-CCG-1, Arg-TCT-1, Gly-GCC-1, His-GTG-1, Ile-GAT-1, Leu-CAA-1, Leu-CAG-1, Leu-CAG-2, Leu-GAG-1, Leu-TAG-1, Leu-TAG-2, Met-CAT-1, Met-CAT-2, Pro-TGG-1, Pro-TGG-2, Thr-GGT-1, Trp-CCA-1, Val-GAC-1, Val-GAC-2, Val-TAC-1. **Right panel.** Heatmap displaying raw Ncleavage values (scale on the color key) from AAS sequencing for D detected in tRNAs across wt, Δ*dusA*, Δ*dusB* and Δ*dusC.*

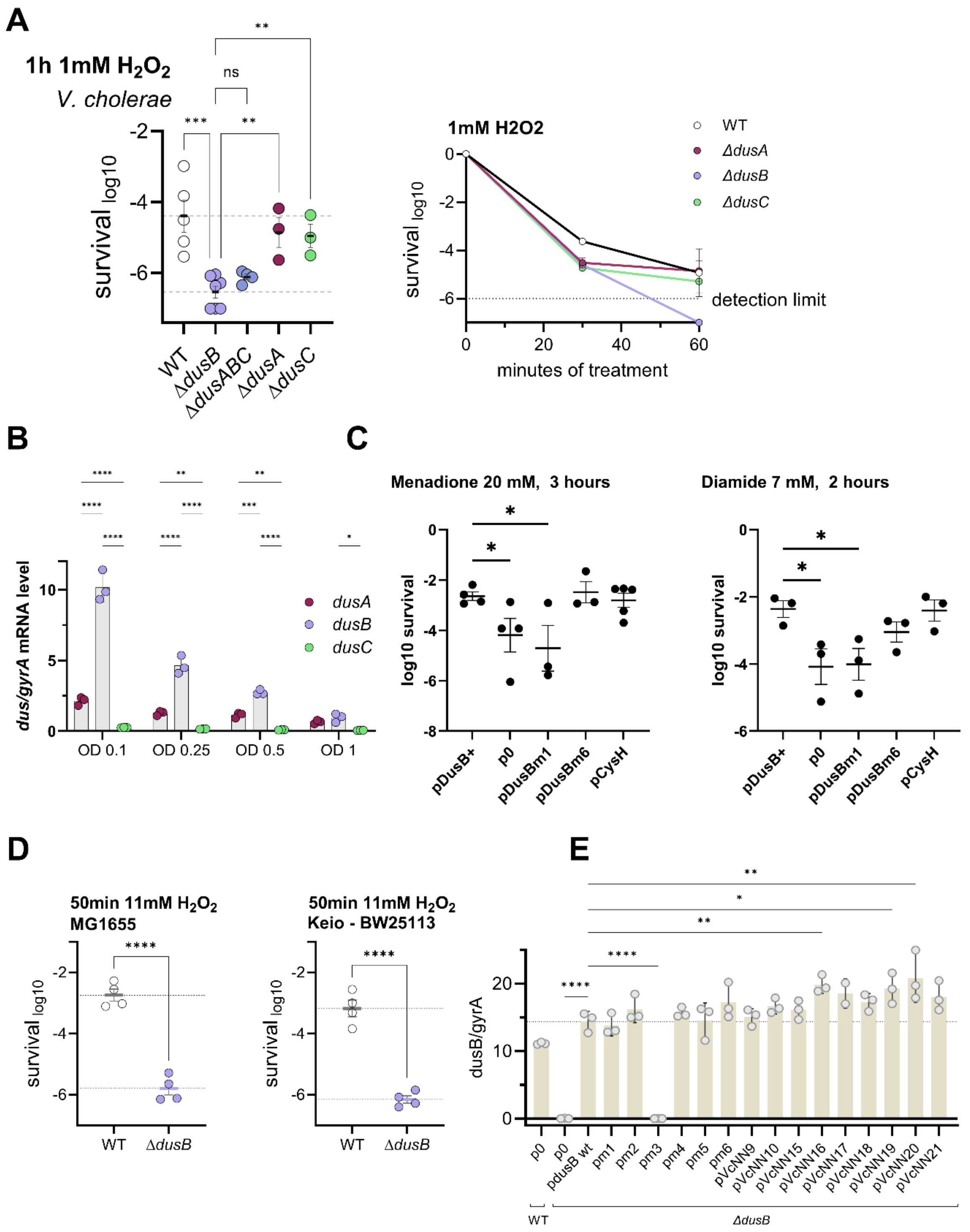

Figure S2: **Dihydrouridine synthase deletions sensitize *V. cholerae* to oxidative stress.** **A.** Log10 survival (y-axis) of exponential phase cultures after 1h (left pannel) or various incubation times (in minutes on the x-axis, right pannel) with 1 mM H_2O_2. n=3-7 **B.** Expression profiles of *dus* genes during growth. mRNA levels of *dusA*, *dusB*, and *dusC* were quantified by digital RT-PCR at different optical densities (ODs). Expression of all three genes decreased as cultures progressed, but *dusB* remained the most highly expressed at all tested ODs. Expression data were normalized to *gyrA*, which is classically used as a housekeeping gene in *V. cholerae*. **C.** Log10 survival (y-axis) of exponential phase cultures after treatment with menadione (left pannel) or diamide (right panel). n=3-4. **D.** Log10 survival to 1h

treatment with 1 mM H_2O_2 of *E. coli dusB* deletion mutants (MG1655 strain on the left pannel, Keio BW25113 strain on the right pannel). The y-axis represents the log10 survival ratio. n=4. **E.** *dusB* mRNA levels measured by digital RT-PCR. n = 3 biological replicates. Strains are indicated in italic below, plasmids start with "p". p0 : empty pSC101. **ABCDE**. Means and standard errors are shown. For multiple comparisons, we used one-way ANOVA. ∗ means $P < 0.05$; ns means not significant.

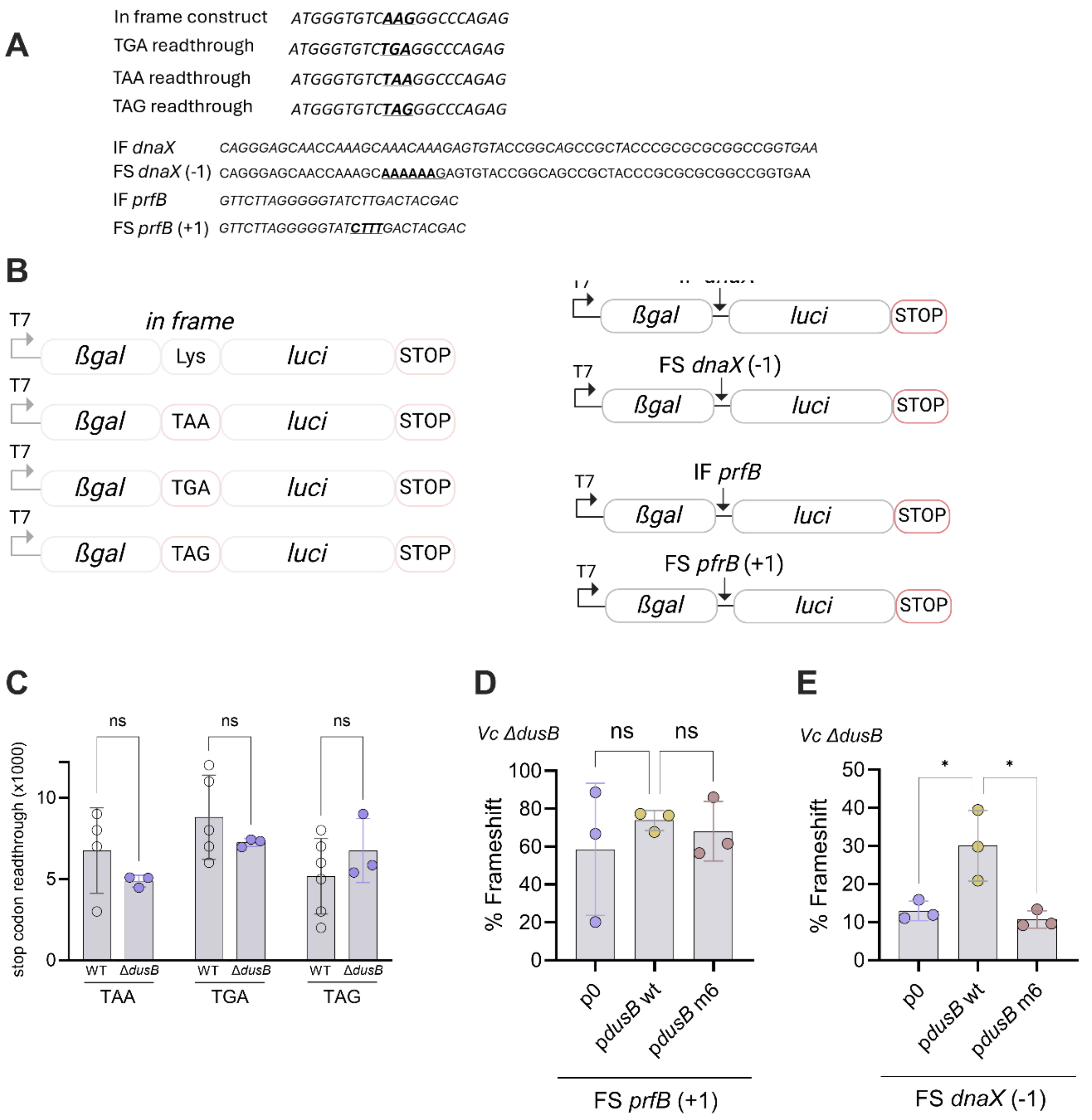

Figure S3 : Quantification of readthrough and frameshift events using a dual-reporter assay. We investigated whether DusB and D17 modifications influence translation processivity, particularly the occurrence of readthrough and frameshift events. To measure these, we used the bacterial pCL99 lacZ-*luc* dual reporter system (Fabret and Namy, 2021). In this system, the *luc* gene, encoding the luciferase, is fused either in frame, in the -1 or +1 frame relative to the lacZ gene or separated from *lacZ* by a stop codon (TGA, TAA, or TAG), which encodes the ß- galactosidase and serves as an internal control for translation efficiency. Luciferase activity in this context provides a direct measure of readthrough or frameshifting efficiency. **A. Top pannel :** Sequences inserted between the *lacZ* and *luc* genes to introduce stop codons into the reporters. In the *in frame* control construct, a Lysine codon (AAG) was inserted instead. **Bottom pannel :** Sequences inserted between the *lacZ* and *luc* genes to introduce frameshifts in the reporters. “IF” stands for *in frame*, indicating that the sequence aligns *luc* with *lacZ,* maintaining the correct reading frame while “FS” indicates a frameshift. The -1 frameshift is introduced using the *dnaX* gene sequence, and the +1 frameshift is generated using the sequence from the *prbB* gene. **B.** Scheme illustrating the 4 dual reporters used for measuring stop codon readthrough frequency and the 4 used for measuring -1 and +1 frameshifting events. **C.** Readthrough quantification for each stop codon. The luciferase/ß-galactosidase ratio obtained for each readthrough reporter has

been normalized to the in-frame control ratio and multiplied by 1000. For multiple comparisons, we used two-way ANOVA. ns indicates not significant. **D.** Quantification of +1 frameshifting. The luciferase/ß-galactosidase ratio obtained for the "*prfB* FS-1" reporter has been normalized to the in-frame control "*prfB* IF" ratio and multiplied by 100 to represent the percentage of frameshift events. **E.** Quantification of -1 frameshift, using the same method but the *dnaX*-reporters. Contrary to H_2O_2 survival phenotypes, the impact of DusB on ribosomal frameshifting depends on its capacity to catalyze the D17 modification. Means and geometric means for logarithmic values were calculated. For multiple comparisons, we used one-way ANOVA. * indicates $p<0.05$. ns indicates not significant. Each experiment was conducted in triplicate, with each dot representing an individual replicate.

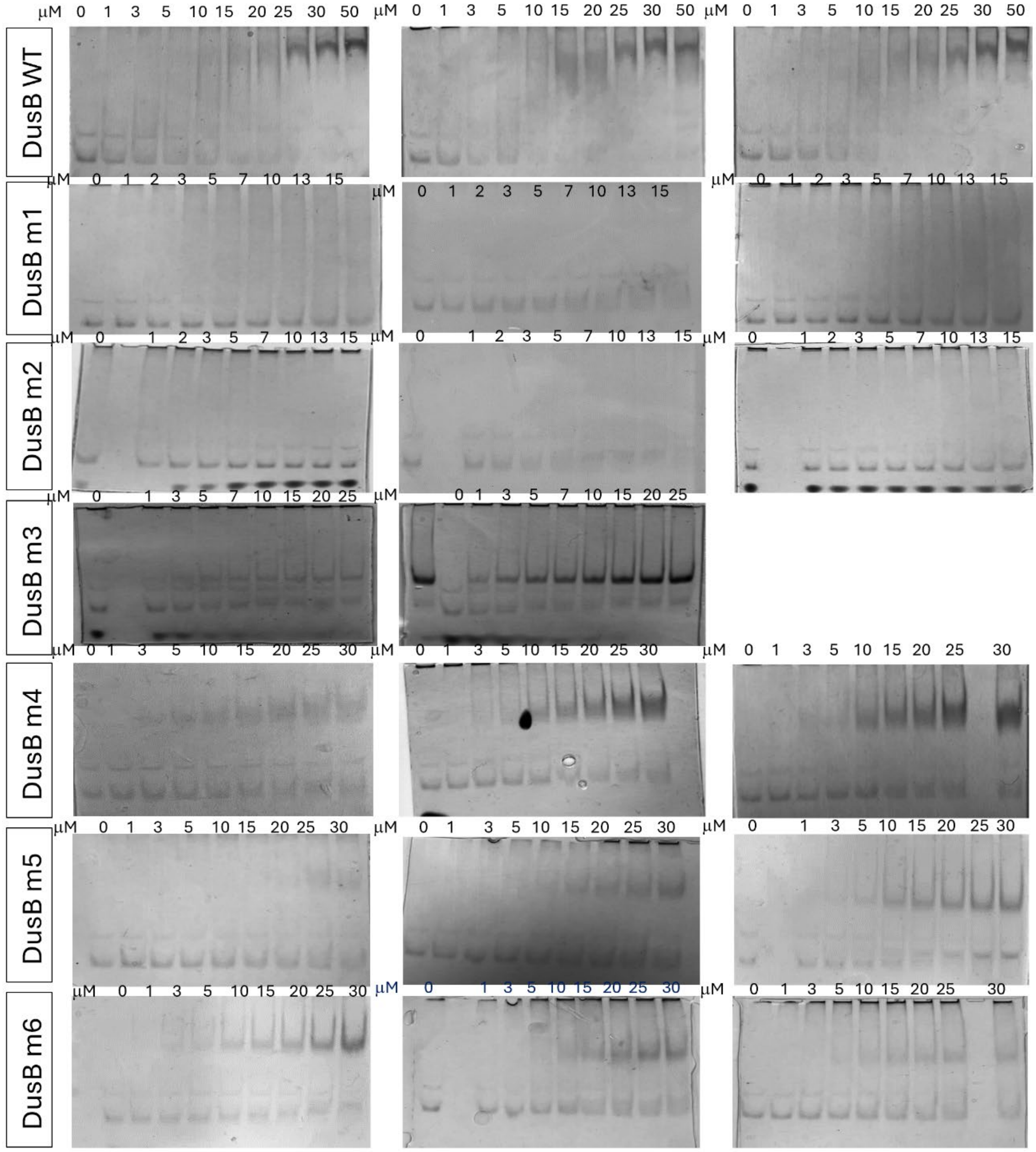

Figure S4: Characterization of interactions between tRNAs and VcDusB variants by electrophoretic mobility shift assay (EMSA). A constant concentration of *Vc* Δ*dusB* tRNA-enriched RNA extracts was incubated with increasing concentrations of each DusB point mutants (from left to right). The EMSA was performed in triplicates.

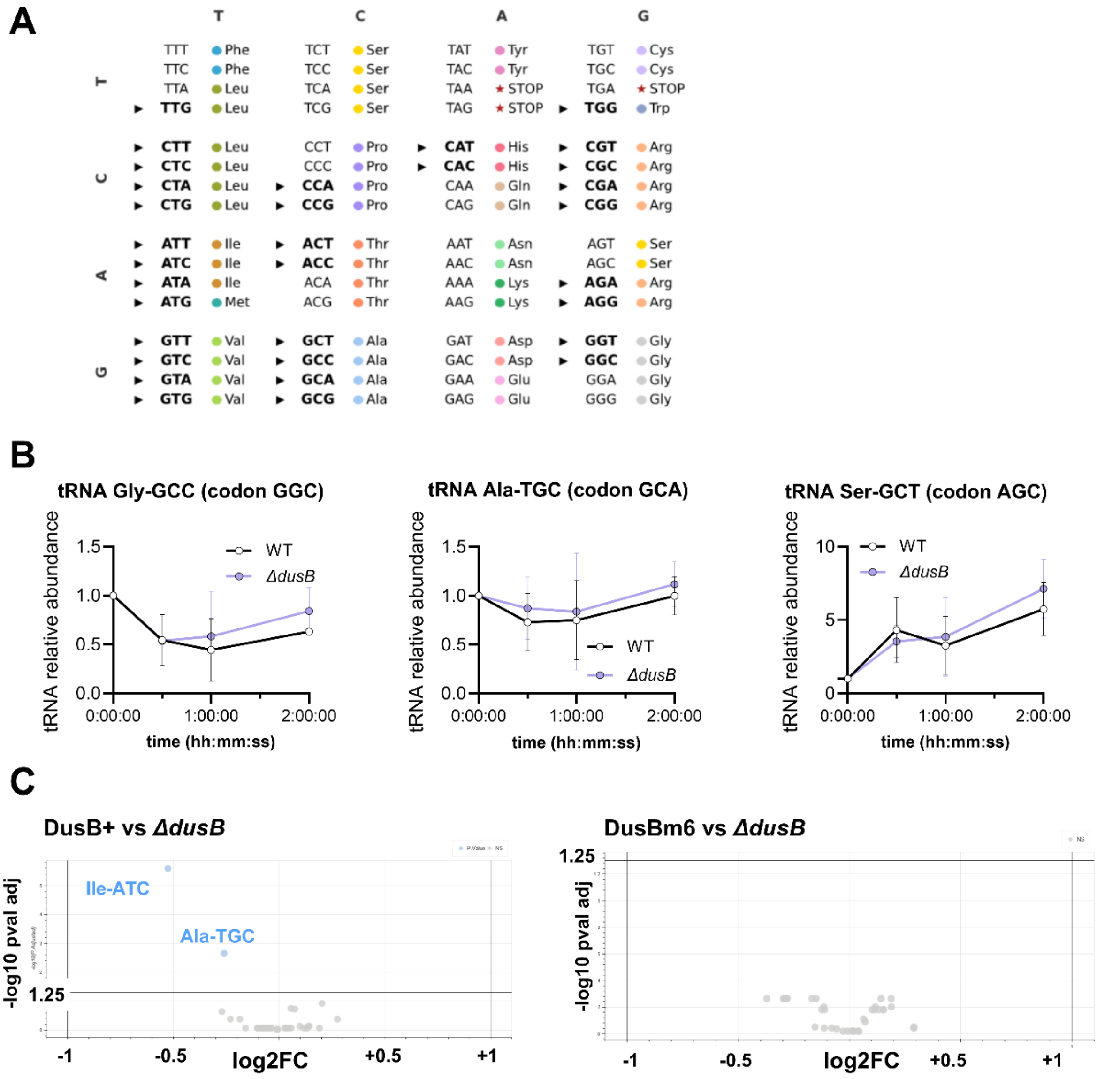

Figure S5: DusB does not generally impact tRNA abundance or stability. A. Codons decoded by DusB-modified tRNAs in *Vibrio cholerae*. Codons shown in bold with a black chevron are decoded by at least one tRNA that can be modified by DusB (exhibiting D17). Assignments are based on AlkAniline-seq data. **B.** Northern blot decay curves of tRNAs in stationary-phase cultures exposed to sublethal H_2O_2. The y-axis shows tRNA abundance relative to 5S rRNA, measured at various time points (x-axis) after transcription was stopped (t0). tRNA levels are normalized against 5S rRNA, with abundance at each time point expressed as the ratio to initial normalized abundance (t0). Each data point reflects the average value from 3 independent replicate. **C.** tRNA direct sequencing using nanopore on tRNA extracts. Comparisons for indicated strains.

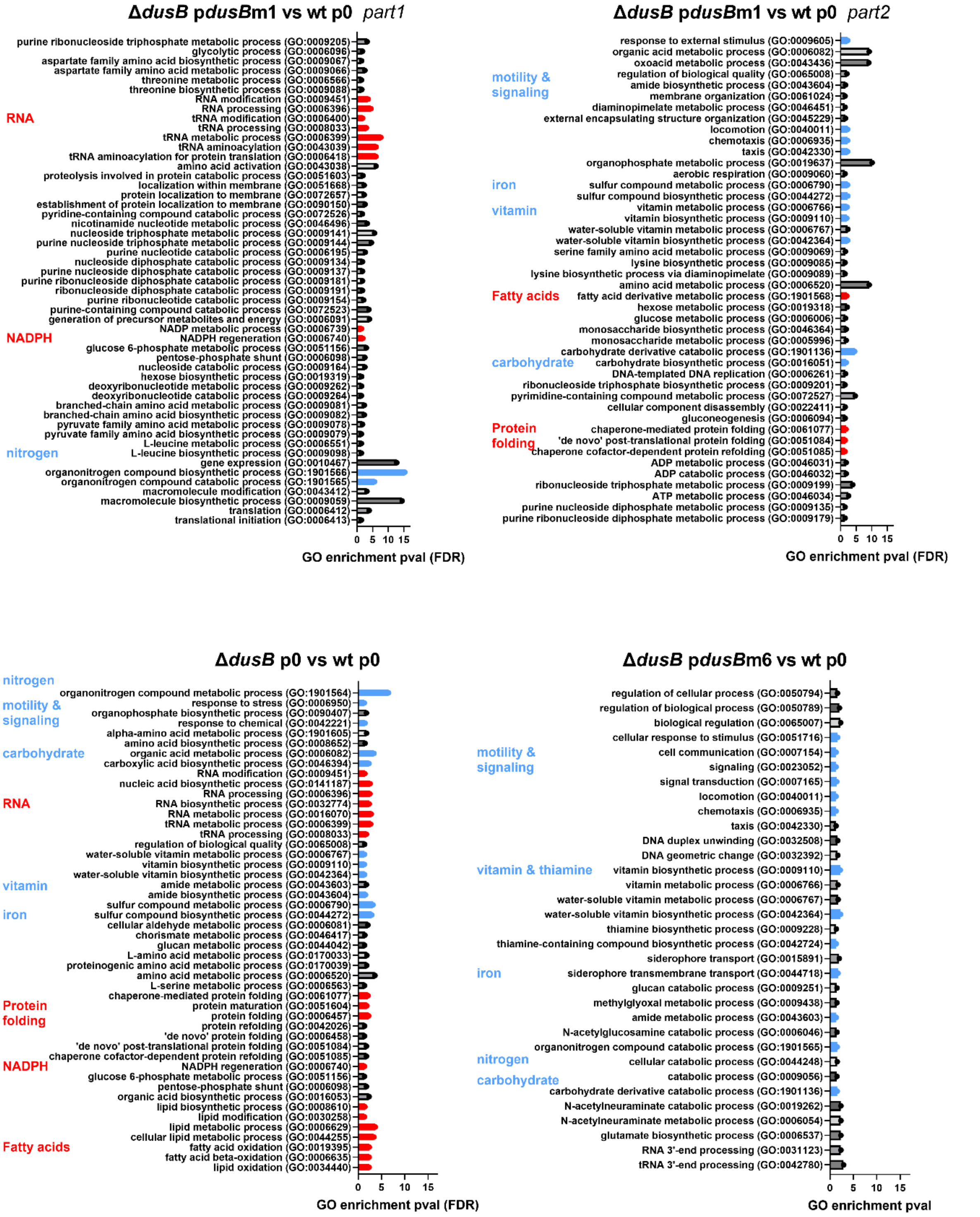

Figure S6: DusB regulates the abundance of proteins involved in RNA, protein folding, NADPH, and fatty acid metabolism, independently of D17. Gene ontology (GO) enrichment analysis in *V. cholerae* of overrepresented gene categories among at least 2-fold differentially abundant proteins in **(A)** Δ*dusB* complemented with p*dusB* m1 variant compared to wt p0, **(B)** ΔdusB p0 compared to wt p0 and **(C)** Δ*dusB* complemented with p*dusB* m6 variant compared to wt p0. GO categories enriched in proteins that are at least 2-fold differentially abundant in strains lacking D17 (compared to wt) and not restored to native level when complemented with a DusB protein retaining tRNA binding and NADPH oxidative functions (*i.e.* enriched in Δ*dusB* p0, Δ*dusB* p*dusB*m1 and Δ*dusB* p*dusB*m6, compared to wt p0), include

those related to nitrogen metabolism, motility and signaling, carbohydrate, vitamin and iron (indicated in blue). Conversely, GO categories whose enrichment is associated to the lack of tRNA binding and/or NADPH oxidative properties (*i.e.* enriched in Δ*dusB* p0, Δ*dusB* p*dusB*m1 but not in Δ*dusB* p*dusB*m6, compared to wt p0) are associated with RNA processing, protein folding, NADPH metabolism and fatty acids metabolism (indicated in red).

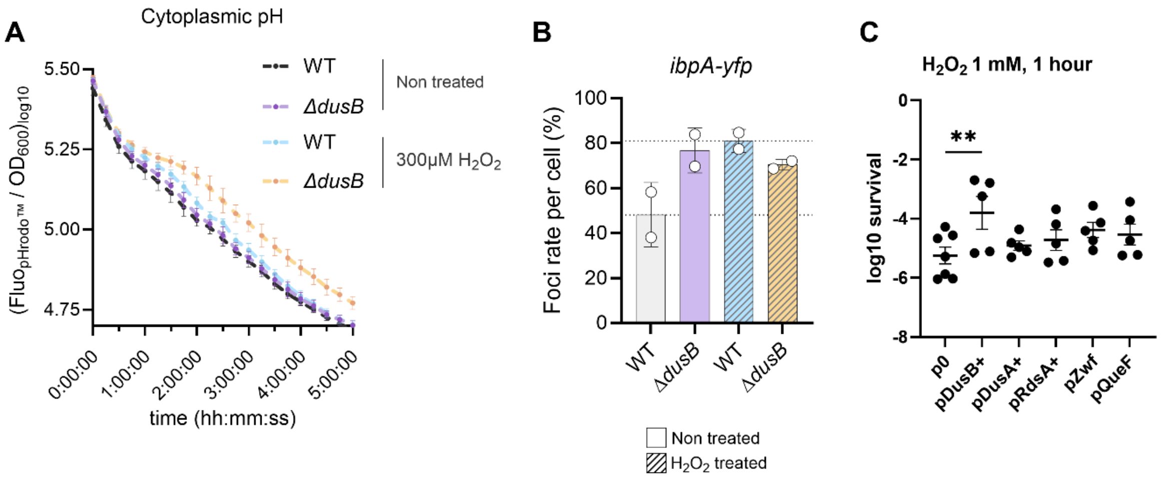

Figure S7: A. IbpA-YFP foci formation in *V. cholerae* wt and *ΔdusB* strains. IbpA-YFP marks protein aggregates. The foci rate per cell (%) was calculated as the number of IbpA-YFP foci normalized to the total cells analyzed. As detailed in the Material and methods section, Fiji (MicrobeJ plugin) was used for image analysis (total cells count in the analysis : 65 < n < 290). The histogram represents WT and ΔdusB cells, with solid bars for non-treated and stripped bars for treated samples. **B**. Cytoplasmic pH dynamics in *V. cholerae* wt and *ΔdusB* strains over time. pHrodo fluorescence (560/590nm, Ex/Em), which increases with acidity, was used as a proxy for intracellular pH changes. The log_{10}-transformed $Fluo_{pHrodo}/OD_{600}$ ratio was plotted on the y-axis. **C**. Low copy plasmids expressing the indicated gene was introduced in ΔdusB and Log10 survival (y-axis) of exponential phase cultures after 1h (left pannel) or various incubation times (in minutes on the x-axis, right pannel) with 1 mM H_2O_2 was measured. n=5-7.

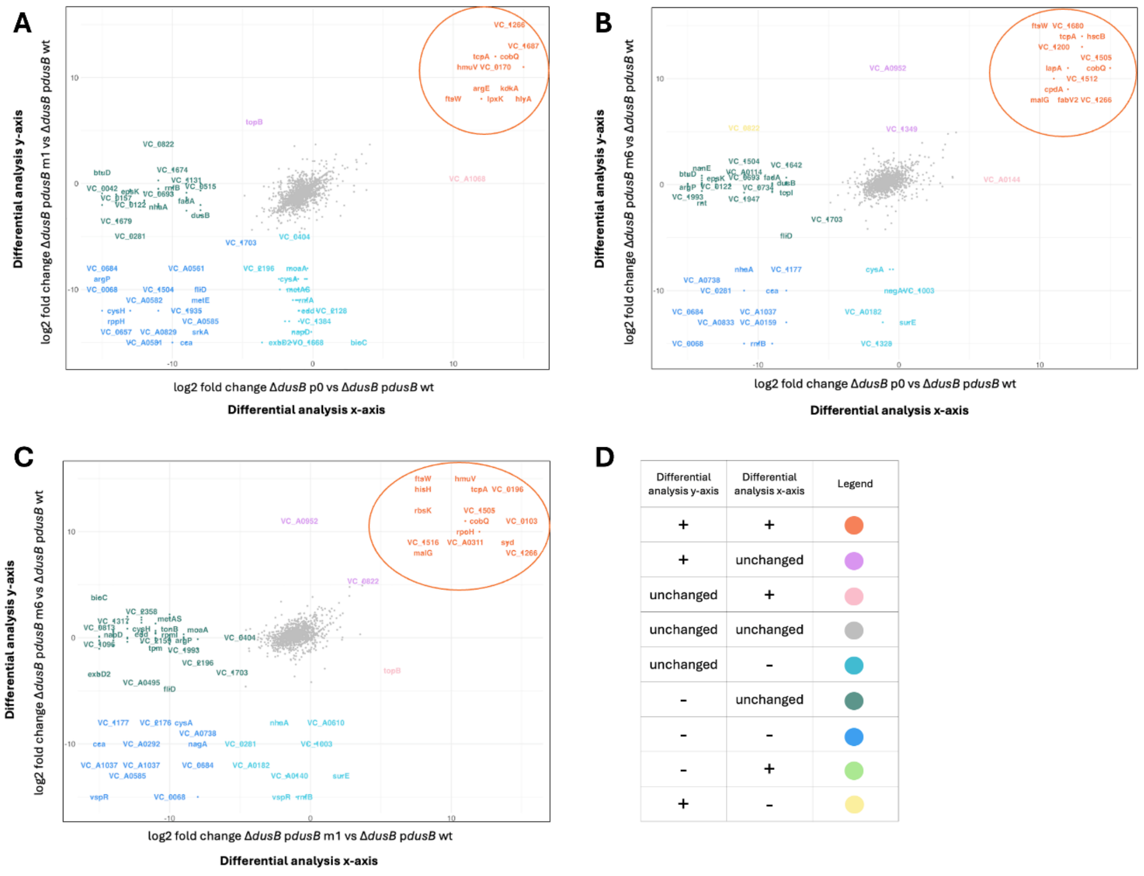

Figure S8: Comparative fold change analysis of protein expression. Plots visualizing differential proteomic analysis across our different conditions. In panel **A**, the y-axis represents the log2 fold change of the protein abundance between the *ΔdusB* strain complemented with the *dusB* m1 variant and the *ΔdusB* strain complemented with the native *dusB*. The x-axis represents the log2 fold change between the *ΔdusB* strain without complementation (empty plasmid, p0) and the same strain complemented with the *dusB* wt. In **B**, the y-axis shows the log2 fold change for the *ΔdusB* strain complemented with the *dusB* m6 variant versus Δ*dusB* complemented with wt *dusB*, while the x-axis shows the same comparison as in Panel A. In **C**, the y-axis displays the comparison between the *ΔdusB* strain complemented with the *dusB* m6 variant versus the Δ*dusB* complemented with wt *dusB*, and the x-axis compares the *ΔdusB* strain complemented with the *dusB* m1 against Δ*dusB* complemented with wt *dusB.* Proteins identified exclusively in one condition are assigned random log2 fold change values between ±8 and ±15, labeled as "more abundant" or "less abundant" compared to the other condition. Color coding in the plots is as follows : proteins more abundant in both differential analyses are highlighted in **orange** and encircled for emphasis ; proteins more abundant in the y-axis comparison but with similar abundance in the x-axis comparison are in **purple** ; proteins more abundant in the x-axis comparison but similarly according to the y-axis comparison are in **pink** ; proteins whose abundance is unchanged as reported by both comparisons are in **grey** ; proteins less abundant in the x-axis comparison but similarly abundant in the y-axis comparison are in **light blue** ; proteins less abundant in the y-axis comparison but similarly abundant in the x-axis comparison are in **dark green** ; proteins less abundant according to both differential analyses are in **dark blue** ; proteins less abundant in the y-axis comparison and more abundant in the x-axis comparison are in **light green** ; proteins less abundant in the x-axis comparison and more abundant in the y-axis comparison are in **yellow**. Legend is displayed in **D** : '*+*' indicates that the protein is more abundant in the test condition compared to the control, '*unchanged*' signifies similar protein abundance between the two strains, and '*-*' denotes that the protein is less abundant in the test condition relative to the control.

Table S1: Summary of tRNAs features.

tRNA anticodon (isoacceptors)	Decoded codons	tRNA anticodon (isodecoders)	copy number	U17	D17
Ala-GGC	GCT, GCC	**Ala-GGC-1**	1	**U17**	**D17**
Ala-TGC	GCA, GCG	**Ala-TGC-1**	3	**U17**	**D17**
		**Ala-TGC-2**	1	**U17**	**D17**
Arg-ACG	CGT, CGC, CGA	**Arg-ACG-1**	2	**U17**	**D17**
		**Arg-ACG-2**	3	**U17**	**D17**
		**Arg-ACG-3**	1	**U17**	**D17**
Arg-CCG	CGG	**Arg-CCG-1**	1	**U17**	**D17**
Arg-TCT	AGA, AGG	**Arg-TCT-1**	1	**U17**	**D17**
Asn-GTT	AAT, AAC	Asn-GTT-1	2	no	no
		Asn-GTT-2	1	no	no
		Asn-GTT-3	1	no	no
Asp-GTC	GAT, GAC	Asp-GTC-1	5	no	no
Cys-GCA	TGT, TGC	Cys-GCA-1	1	no	no
		Cys-GCA-2	2	no	no
fMET-CAT	ATG	fMET-CAT-1	2	U17	no
		fMET-CAT-2	1	U17	no
		fMET-CAT-3	1	U17	no
		fMET-CAT-4	1	U17	no
Gln-TTG	CAA, CAG	Gln-TTG-1	1	no	no
		Gln-TTG-2	3	no	no
		Gln-TTG-3	1	no	no
Glu-TTC	GAA, GAG	Glu-TTC-1	4	no	no
Gly-GCC	GGT, GGC	**Gly-GCC-1**	6	**U17**	**D17**
Gly-TCC	GGA, GGG	Gly-TCC-1	2	no	no
His-GTG	CAT, CAC	**His-GTG-1**	2	**U17**	**D17**
Ile-GAT	ATT, ATC, ATA	**Ile-GAT-1**	3	**U17**	**D17**
Ile2-CAT	ATA	Ile2-CAT-1	1	no	no
Leu-CAA	TTG	**Leu-CAA-1**	1	**U17**	**D17**
Leu-CAG	CTG	**Leu-CAG-1**	2	**U17**	**D17**
		**Leu-CAG-2**	1	**U17**	**D17**
Leu-GAG	CTT, CTC	**Leu-GAG-1**	1	**U17**	**D17**
Leu-TAA	TTA, TTG	Leu-TAA-1	2	no	no
Leu-TAG	CTA, CTG	**Leu-TAG-1**	1	**U17**	**D17**
		**Leu-TAG-2**	4	**U17**	**D17**
Lys-TTT	AAA, AAG	Lys-TTT-1	2	no	no
Met-CAT	ATG	**Met-CAT-1**	2	**U17**	**D17**
		**Met-CAT-2**	1	**U17**	**D17**
Phe-GAA	TTT, TTC	Phe-GAA-1	2	no	no
		Phe-GAA-2	1	no	no
Pro-GGG	CCT, CCC	Pro-GGG-1	1	U17	no

Pro-TGG	CCA, CCG	**Pro-TGG-1**	2	**U17**	**D17**
		**Pro-TGG-2**	1	**U17**	**D17**
Ser-GCT	AGT, AGC	Ser-GCT-1	1	no	no
		Ser-GCT-2	1	no	no
Ser-GGA	TCT, TCC	Ser-GGA-1	1	no	no
Ser-TGA	TCA, TCG	Ser-TGA-1	2	no	no
Thr-GGT	ACT, ACC	**Thr-GGT-1**	1	**U17**	**D17**
		Thr-GGT-2	1	no	no
Thr-TGT	ACA, ACG	Thr-TGT-1	1	no	no
		Thr-TGT-2	3	no	no
Trp-CCA	TGG	**Trp-CCA-1**	1	**U17**	**D17**
Tyr-GTA	TAT, TAC	Tyr-GTA-1	5	no	no
Val-GAC	GTT, GTC	**Val-GAC-1**	1	**U17**	**D17**
		**Val-GAC-2**	1	**U17**	**D17**
Val-TAC	GTA, GTG	**Val-TAC-1**	2	**U17**	**D17**

List of isoacceptor and isodecoder tRNAs, along with the codons decoded by each group. The occurrence of genes corresponding to each isoacceptor group is also indicated (copy number). tRNAs exhibiting a uridine at position 17 are marked with a green "U17" ; tRNAs identified as DusB-modified based on AlkAniline-seq data are associated to a purple “D17” indicator and displayed in blue. Codon recognized by these DusB-substrate tRNAs are indicated in orange. Interestingly, five tRNAs with U17 – Pro-GGG-1 and initiator methionine fMET-CAT-1/2/3/4 – were not modified by VcDusB.

Table S2: RNAseq comparing *ΔdusB* strain to WT

**non treated**			
**locus_tag**	**gene**	**log2FC *ΔdusB*/WT**	**p value (-log10)**
VC_0649		-1,0	1,4
VC_0616		-0,8	1,8
VC_0614		-0,8	1,5
VC_2316	argA	-0,7	1,5
**VC_0179**	**dncV**	0,6	1,6
**VC_0180**		0,6	1,7
**VC_0181**		0,6	1,4
**VC_0178**	**capV**	0,6	1,6
VC_A0732		0,8	2,1
VC_A0648		1,6	1,5
**H2O2**			
**locus_tag**	**gene**	**log2FC *ΔdusB* /WT**	**p value (-log10)**
VC_1176	VC_1176	-2,4	1,8
VC_1861	VC_1861	-1,5	2,0
VC_0649	VC_0649	-1,2	1,8
VC_0075	VC_0075	-1,2	2,0
VC_A0304	VC_A0304	-1,1	1,9
VC_1080	VC_1080	-1,1	1,5
VC_1862	VC_1862	-1,1	1,9
VC_0509	VC_0509	-1,1	1,9
VC_A0691	VC_A0691	-1,0	1,4
VC_0550	oadA	-1,0	1,7
**VC_0179**	**dncV**	1,0	4,2
VC_1801	VC_1801	1,0	1,4
VC_A0569	VC_A0569	1,1	1,9
**VC_0178**	**capV**	1,1	4,0
VC_1495	VC_1495	1,2	1,4
VC_A0237	VC_A0237	1,3	1,7
VC_0804	VC_0804	1,3	1,9
VC_0780	VC_0780	1,4	1,6
**VC_0176**	**VC_0176**	1,5	7,1
VC_0290	fis	2,1	12,8

Table S3. Proteomics

ΔdusB vs WT (25 up, 26 down)			
Uniprot accession	Gene name	log2 FC	logp
Q9KP33	VC_2546	22,3	1,5
Q9KV57	VC_0300	3,9	2,3
P0C6D2	cueR	3,0	1,9
Q9KLY3	VC_A0608	3,0	1,9
Q9KPE9	xerD	2,9	2,7
Q9KQH3	rpmF	2,6	1,8
Q9KVC7	rpmG	2,4	1,3
P57066	lolD	2,2	1,4
Q9KL36	VC_A0913	2,0	2,0
Q9KNT7	argB	1,9	2,2
Q9KQC5	VC_2075	1,9	1,4
O68845	rpmI	1,8	2,3
Q9KSJ1	VC_1265	1,8	1,8
Q9KMT9	VC_A0230 fhuC	1,7	2,3
Q9KVE6	VC_0200 fhuA	1,7	2,3
Q9KMW8	VC_A0198 vchM	1,7	2,3
Q9KQK1	VC_1997	1,5	1,4
Q9KPZ5	VC_2217	1,4	1,9
Q9KPY9	VC_2223 rluF	1,3	1,8
Q9KRL0	VC_1630	1,3	1,5
Q9KR01	ruvA	1,2	1,6
P64127	fis	1,1	1,6
Q9KP15	VC_2564 dbpA	1,1	2,3
Q9KTJ2	VC_0910	1,1	1,8
Q9KNF4	VC_A0010	1,0	1,6
Q9KU72	VC_0651	-1,1	1,4
Q9KLL9	modC	-1,1	1,6
Q9KUU5	VC_0420	-1,1	1,6
Q9KL16	cspV	-1,1	1,3
Q9KL48	VC_A0900	-1,2	1,4
Q9KSG9	opgH	-1,2	1,5
Q9KKT9	VC_A1011	-1,3	2,3
Q9KU40	VC_0689	-1,4	1,7
Q9KKS5	nagB	-1,4	1,9
Q9KQB9	VC_2081	-1,4	1,6
Q9KSY4	VC_1122	-1,5	1,8
Q9KKQ9	VC_A1043	-1,5	1,3

Q9KLC6	groL2	-1,6	1,7
Q9KST8	VC_1168	-1,6	2,3
Q9KKV2	VC_A0998	-1,8	1,3
Q9K2M2	VC_A0347	-1,9	1,6
Q9KRL7	VC_1619	-1,9	1,4
Q9KVL2	cdpA	-2,0	1,4
Q9KQ24	hemA	-2,0	1,8
Q9KTU7	VC_0790	-2,0	1,8
Q9KRG9	VC_1673	-2,4	1,3
Q9KR83	VC_1760	-2,5	2,3
Q9KN44	VC_A0121	-2,5	1,8
Q9KNX5	VC_2605	-2,8	2,2
Q9KPT4	VC_2278	-3,1	2,3
Q9KL09	VC_A0941	-21,5	2,1
ΔdusB p0 vs pDusB+ H2O2 (84 up, 980 down)			
Uniprot accession	Gene name	log2 FC	logp
P09545	hlyA	only in dusB	
P0C6F0	acfD	only in dusB	
Q60153	tcpA	only in dusB	
Q9KKN4	VC_A1068	only in dusB	
Q9KL07	malG	only in dusB	
Q9KL34	hmuV	only in dusB	
Q9KLG0	fabV2	only in dusB	
Q9KLG2	VC_A0782	only in dusB	
Q9KLL6	cobQ	only in dusB	
Q9KLP6	VC_A0697	only in dusB	
Q9KLS5	VC_A0666	only in dusB	
Q9KM49	VC_A0540	only in dusB	
Q9KM77	VC_A0511	only in dusB	
Q9KM93	VC_A0488	only in dusB	
Q9KMA3	VC_A0474	only in dusB	
Q9KMB4	VC_A0459	only in dusB	
Q9KMP6	VC_A0274	only in dusB	
Q9KN30	VC_A0136	only in dusB	
Q9KNC7	VC_A0038	only in dusB	
Q9KNT5	argE	only in dusB	
Q9KP10	VC_2569	only in dusB	
Q9KPD7	cpdA	only in dusB	
Q9KPG6	ftsW	only in dusB	

Q9KQT5	lapA	only in dusB	
Q9KQX0	lpxK	only in dusB	
Q9KRD2	VC_1710	only in dusB	
Q9KRF5	VC_1687	only in dusB	
Q9KRG2	VC_1680	only in dusB	
Q9KRW8	VC_1516	only in dusB	
Q9KRX1	VC_1512	only in dusB	
Q9KRX8	VC_1505	only in dusB	
Q9KRZ9	rmf	only in dusB	
Q9KS92	VC_1367	only in dusB	
Q9KSB2	maiA	only in dusB	
Q9KSJ0	VC_1266	only in dusB	
Q9KSQ6	VC_1200	only in dusB	
Q9KT16	VC_1089	only in dusB	
Q9KTM6	VC_0876	only in dusB	
Q9KTX9	hscB	only in dusB	
Q9KU94	VC_0629	only in dusB	
Q9KVB9	kdkA	only in dusB	
Q9KVH6	VC_0170	only in dusB	
Q9KN22	VC_A0144	7,3	6,1
Q9KVV8	VC_0030	4,1	4,5
H9L4T5	VC_0847	3,8	3,9
Q9KST8	VC_1168	3,8	3,9
Q9KUM9	VC_0486	3,5	3,9
Q9KSZ2	bioC	3,3	2,3
Q9KP97	VC_2476	3,1	4,1
Q9KQY2	VC_1865	3,1	3,3
Q9KV41	VC_0317	3,1	3,9
Q9KKX6	VC_A0974	2,9	2,4
Q9KLY3	VC_A0608	2,5	2,1
Q9KUR0	VC_0455	2,4	3,7
P52695	hlyU	2,3	3,5
Q9KNW4	astD	2,2	1,8
Q9KMV8	VC_A0210	2,2	3,6
Q9KQK6	purU	2,1	3,5
Q9KST9	tdk	2,1	3,7
Q9KLE6	VC_A0800	2,0	1,9
Q9KNX0	VC_2610	2,0	3,2
Q9KNA0	VC_A0065	1,8	3,4
Q9KM82	VC_A0501	1,8	3,0
Q9KUX4	cysJ	1,7	1,6

Q9KRF7	VC_1685	1,7	2,1
Q9KQV6	VC_1892	1,7	3,2
Q9KNS0	VC_2661	1,6	2,1
Q9KSK9	VC_1247	1,5	2,0
Q9KQL5	VC_1983	1,5	3,4
Q9KTA0	VC_1003	1,4	2,4
Q9KN33	VC_A0132	1,4	3,8
Q9KSE9	VC_1308	1,4	2,8
Q9KQ73	VC_2128	1,3	2,6
Q9KL86	VC_A0860	1,3	2,2
Q9KMP1	VC_A0283	1,3	2,3
Q9KRK7	VC_1633	1,3	2,3
Q9KLW4	VC_A0627	1,3	2,3
Q9KP95	VC_2478	1,3	2,7
Q9KQE8	VC_2052	1,3	2,0
Q9KVE7	VC_0199	1,2	2,0
Q9KSK6	VC_1250	1,2	3,6
Q9KM71	VC_A0517	1,1	1,8
Q9KUB7	VC_0605	1,1	2,0
Q9KN58	VC_A0107	1,0	1,6
Q9KTB1	VC_0992	1,0	1,5
Q9KUE8	VC_0573	-1,0	2,4
Q9KS93	queC	-1,0	1,4
Q9KVV0	VC_0039	-1,0	1,4
Q9KLK6	luxP	-1,0	2,8
Q9KSK8	VC_1248	-1,0	1,9
Q9KPW2	lpxD	-1,0	2,6
Q9KTA8	VC_0995	-1,0	2,8
Q9KVY2	rnpA	-1,0	1,8
P15795	toxR	-1,0	2,5
Q9KRZ2	pyrD	-1,0	1,8
Q9KTY2	iscS	-1,0	2,5
Q9KNY6	rplW	-1,0	2,1
Q9KPW0	bamA	-1,0	2,4
Q9KQV8	VC_1890	-1,0	2,1
Q9KL75	VC_A0872	-1,0	1,3
Q9KUI3	VC_0538	-1,0	2,4
Q9KNK0	pckA	-1,0	2,4
Q9KM87	VC_A0495	-1,0	1,8
Q9KUC8	pcnB	-1,0	2,1
Q9KP58	VC_2518	-1,0	1,7

Q9KU97	hemL	-1,0	2,5
Q9KVI2	VC_0164	-1,0	1,8
Q9KNX8	purA	-1,0	2,4
Q9KRH8	VC_1664	-1,0	1,8
Q9KM37	VC_A0552	-1,0	2,0
Q9KKM0	VC_A1083	-1,0	2,1
Q9KS02	matP	-1,0	1,7
Q9KL52	zwf	-1,0	2,2
Q9KUR3	VC_0452	-1,0	2,0
Q9KLJ2	VC_A0752	-1,0	1,5
Q9KR12	pal	-1,0	1,9
Q9KNQ6	hslV	-1,0	2,2
Q9KUE3	VC_0578	-1,0	1,8
Q9KLF1	VC_A0795	-1,0	1,6
Q9KL28	VC_A0921	-1,0	2,1
Q9KM60	VC_A0528	-1,0	2,0
Q9KMW3	VC_A0205	-1,0	1,7
Q9KUG1	ffh	-1,0	2,4
Q9KPH4	secA	-1,0	2,5
Q9KSP7	VC_1209	-1,0	2,2
Q9KU75	VC_0648	-1,0	2,0
Q9KR03	VC_1844	-1,0	2,6
Q9KVR1	VC_0078	-1,0	2,8
Q9KSR1	VC_1195	-1,0	2,4
Q9KTD7	VC_0965	-1,0	2,7
Q9KV99	VC_0251	-1,0	2,3
Q9KQJ2	VC_2006	-1,0	1,3
Q9KSX9	hflD	-1,0	2,5
Q9KUT3	mdh	-1,0	2,5
Q9KPH9	carB	-1,0	2,6
Q9KKZ7	VC_A0953	-1,0	1,8
Q9KQS7	clpX	-1,0	2,6
Q9KLW0	VC_A0631	-1,0	2,0
O68844	infC	-1,0	2,7
Q9KTZ1	queA	-1,0	2,6
Q9KRY7	VC_1496	-1,0	2,5
Q9KM23	VC_A0566	-1,0	2,6
Q9KU91	VC_0632	-1,0	2,5
Q9KNZ9	rplF	-1,0	2,7
Q9KP50	lptA	-1,0	2,7
Q9KTZ3	VC_0737	-1,0	1,6

Q9KQC6	argS	-1,0	2,8
Q9KRT1	VC_1555	-1,0	2,8
Q9KR78	VC_1765	-1,0	2,7
Q9KTX7	VC_0753	-1,0	2,3
Q9KPV5	frr	-1,0	2,2
Q9KKT0	VC_A1020	-1,0	2,1
Q9KVX0	VC_0018	-1,0	3,0
Q9KQ06	cheR1	-1,0	3,5
Q9KS00	fabA	-1,0	3,5
Q9KP40	VC_2539	-1,0	3,3
Q9KNQ2	rpmE	-1,0	3,5
Q9KLD2	VC_A0814	-1,0	3,5
Q9KM75	VC_A0513	-1,0	3,3
Q9KPB2	rnc	-1,0	3,3
Q9KPI2	VC_2386	-1,0	2,1
Q9KV48	ubiD	-1,0	3,5
Q9KNN0	VC_2702	-1,0	2,4
Q9KU21	bamD	-1,0	3,5
Q9KQZ3	VC_1854	-1,0	3,4
P45780	epsF	-1,0	3,5
Q9KUY0	fur	-1,0	2,8
Q9KUP9	VC_0466	-1,0	3,0
Q9KSX6	VC_1130	-1,0	3,4
Q9KVF9	rlmJ	-1,0	2,6
Q9KRU4	norM	-1,0	3,1
Q9KM78	VC_A0510	-1,0	2,4
H9L4S9	VC_0415	-1,0	3,4
Q9KS73	VC_1386	-1,0	2,0
Q9KKX5	VC_A0975	-1,0	3,2
Q9KPD8	VC_2432	-1,0	2,2
Q9KTD2	zipA	-1,0	3,4
Q9KT51	dnaX	-1,0	2,7
Q9KVM7	yihI	-1,0	3,5
Q9KRA3	fabV	-1,0	3,2
Q9KUE5	VC_0576	-1,0	3,6
Q9KVX3	gyrB	-1,0	3,5
Q9KPM4	radA	-1,0	3,5
Q9KKY0	ppnP	-1,0	2,9
Q9KNX2	VC_2608	-1,0	3,5
Q9KRL0	VC_1630	-1,0	2,5
Q9KMZ0	VC_A0176	-1,0	3,6

Q9KPW1	skp	-1,0	3,2
Q9KPM2	VC_2345	-1,0	2,0
Q9KLA1	VC_A0845	-1,0	2,8
Q9KPD6	VC_2434	-1,0	2,8
Q9KKJ3	VC_A1114	-1,0	3,3
Q56648	alaS	-1,0	3,5
Q9KRQ1	VC_1585	-1,0	3,1
Q9KRU6	VC_1538	-1,0	3,5
Q9KRM5	metAS	-1,0	2,6
Q9KT26	VC_1079	-1,0	3,4
Q9KSG1	VC_1295	-1,0	2,1
Q9KKV3	VC_A0997	-1,0	3,5
Q9KPF5	VC_2413	-1,0	3,4
Q9KNV2	aroB	-1,0	3,1
Q9KTB7	adk	-1,0	3,4
Q9KQD3	VC_2067	-1,0	3,0
Q9KNR7	groL1	-1,0	2,4
Q9KRH9	VC_1663	-1,0	3,6
Q9KLJ3	fabH2	-1,0	2,8
Q9KNZ2	rpmC	-1,0	3,0
Q9KVJ6	ftsY	-1,0	3,0
O34241	dnaK	-1,0	3,1
Q9KL50	VC_A0898	-1,0	3,5
Q9KTD8	VC_0964	-1,0	3,1
Q9KPS3	VC_2289	-1,0	3,2
Q9KQB9	VC_2081	-1,0	1,6
Q9KLJ9	glpK	-1,0	3,0
Q9KT69	metG	-1,0	3,6
Q9KS36	VC_1425	-1,0	3,5
Q9KUP8	VC_0467	-1,0	1,6
Q9KPC4	pyrG	-1,0	3,6
P45383	recA	-1,0	3,5
Q9KVF8	VC_0188	-1,0	3,0
Q9KUS3	apaG	-1,0	3,5
Q9KUG9	VC_0552	-1,0	3,0
O34419	rstR1	-1,0	3,3
P23247	asd2	-1,0	3,5
Q9KUQ7	VC_0458	-1,0	2,8
Q9KSI8	VC_1268	-1,0	2,9
Q9KUQ5	proC	-1,0	2,8
Q9KP63	VC_2513	-1,0	2,6

Q9KUG3	VC_0558	-1,0	2,9
Q9KSF8	VC_1298	-1,0	3,4
Q9KS24	VC_1437	-1,0	3,5
Q9KTA5	VC_0998	-1,0	2,9
Q9KN98	VC_A0067	-1,0	2,3
Q9KPT3	VC_2279	-1,1	3,4
Q9KVL7	lysA	-1,1	3,4
Q9KT67	udk	-1,1	3,6
Q9KN62	VC_A0103	-1,1	3,5
Q9KQI1	mltG	-1,1	3,1
Q9KQT9	VC_1909	-1,1	3,3
Q9KL51	pgl	-1,1	3,4
Q9KVP1	glpE	-1,1	2,1
Q9KUP3	metK	-1,1	3,5
Q9KRQ7	VC_1579	-1,1	3,5
Q9KVB2	VC_0234	-1,1	3,1
Q9KM92	VC_A0489	-1,1	1,8
Q9KKS1	VC_A1029	-1,1	3,4
Q9KSL4	astE	-1,1	3,5
Q9KQW2	mfd	-1,1	3,5
Q9KM67	sbcC	-1,1	3,5
Q9KTE0	miaB	-1,1	3,4
P0C6P9	tpx	-1,1	3,1
Q9KVX5	dnaN	-1,1	3,4
Q9KKS9	VC_A1021	-1,1	3,5
Q9KST6	trpB	-1,1	3,2
Q9KRD3	VC_1709	-1,1	3,5
Q9KS97	VC_1362	-1,1	3,1
Q9KNA9	VC_A0056	-1,1	3,3
Q9KN63	VC_A0102	-1,1	3,1
Q9KLW3	VC_A0628	-1,1	3,3
Q9KV85	VC_0271	-1,1	2,2
Q9KQS8	lon	-1,1	2,9
Q9KVR3	VC_0076	-1,1	2,8
Q9KU60	lysS	-1,1	3,6
Q9KUD7	VC_0585	-1,1	3,6
Q9KUG4	luxS	-1,1	3,4
Q9KQF9	VC_2039	-1,1	3,0
Q9KKP3	VC_A1059	-1,1	3,5
Q9KU27	VC_0702	-1,1	3,5
Q9KV04	VC_0354	-1,1	3,6

Q9KUH1	VC_0550	-1,1	3,5
Q9KQH6	VC_2022	-1,1	3,5
Q9KSL0	VC_1246	-1,1	3,4
Q9KUW2	ssb	-1,1	3,3
Q9KPJ9	VC_2368	-1,1	3,6
Q9KQB4	VC_2086	-1,1	3,1
Q9KPD9	parE	-1,1	3,5
Q9KQ57	VC_2147	-1,1	3,5
Q9KVH9	rep	-1,1	3,5
Q9KUV7	VC_0405	-1,1	3,5
Q9KQ01	VC_2206	-1,1	2,6
Q9KUR4	VC_0451	-1,1	2,9
Q9KUJ8	VC_0522	-1,1	2,8
Q9KLH0	VC_A0774	-1,1	3,5
Q9KN99	VC_A0066	-1,1	2,2
Q9KLD8	VC_A0808	-1,1	3,3
Q9KMX7	VC_A0189	-1,1	3,5
Q9KSK4	VC_1252	-1,1	3,4
Q9KRQ9	VC_1577	-1,1	3,6
Q9KSE5	bsrV	-1,1	3,4
Q9KQ66	VC_2137	-1,1	3,4
Q9KPN7	dapD	-1,1	3,6
Q9KMG2	VC_A0396	-1,1	3,3
Q9KUE7	VC_0574	-1,1	3,4
Q9KTE6	leuS	-1,1	3,6
Q9KSS5	VC_1181	-1,1	2,9
Q9KNJ4	VC_2744	-1,1	3,5
Q9KT79	moaC	-1,1	3,4
Q9KNQ0	VC_2681	-1,1	3,6
Q9KM18	VC_A0571	-1,1	3,4
Q9KQH0	rluC	-1,1	3,5
Q9KVB5	VC_0231	-1,1	3,3
Q9KV89	VC_0267	-1,1	2,2
Q9KUG0	rpsP	-1,1	3,4
Q9KLA9	VC_A0837	-1,1	3,5
Q9KMN1	VC_A0300	-1,1	3,6
P22359	htpG	-1,1	3,0
Q9KRL1	VC_1629	-1,1	2,9
Q9KMP9	VC_A0271	-1,1	2,2
Q9KV10	hflX	-1,1	3,6
Q9KP91	VC_2482	-1,1	3,1

Q9KVY4	yidC	-1,1	3,2
Q9KUB8	VC_0604	-1,1	3,6
P0C6E1	pepA	-1,1	2,8
Q9KUG5	gshA	-1,1	3,4
Q9KQE3	ccmA	-1,1	3,0
Q9KRV1	VC_1533	-1,1	3,1
Q9KQ10	VC_2197	-1,1	3,2
Q9KP34	ppa	-1,1	3,6
Q9KPH7	dapB	-1,1	3,2
Q9KP37	mpl	-1,1	2,2
Q9KR86	VC_1756	-1,1	3,5
Q9KTF1	rlmH	-1,1	3,4
Q9KPS2	nqrB	-1,1	3,6
Q9KUR9	lptD	-1,1	3,6
Q9KRK5	rsuA	-1,1	3,2
Q9KKM8	VC_A1075	-1,1	3,1
Q9KNF4	VC_A0010	-1,1	2,5
Q9KRZ8	VC_1485	-1,1	3,3
Q9KSX8	mnmA	-1,1	3,5
Q9KPG4	mraY	-1,1	2,4
Q9KLX4	VC_A0617	-1,1	3,5
Q9KTF2	mrdA	-1,1	2,9
Q9KNU2	VC_2638	-1,1	2,4
Q9KU18	clpB	-1,1	3,3
Q9KSP3	VC_1213	-1,1	3,5
Q9KSF9	asnS	-1,1	3,6
Q9KSU6	VC_1160	-1,1	3,4
Q9X4Q6	nqrD	-1,1	3,0
Q9KUF3	zapE	-1,1	3,6
Q9KQ29	kdsA	-1,1	3,5
Q9KPF9	rsmH	-1,1	3,7
Q9KPU2	VC_2270	-1,1	3,5
Q9KP03	rplO	-1,1	3,6
Q9KSY0	VC_1126	-1,1	3,6
Q9KLT2	VC_A0659	-1,1	2,8
Q9KP75	VC_2500	-1,1	2,6
Q9KVS3	VC_0066	-1,1	3,1
Q9KUX7	VC_0381	-1,1	3,0
Q9KVG7	dncV	-1,1	3,4
Q9KMU2	VC_A0227	-1,1	3,5
Q9KRI6	VC_1655	-1,1	1,9

Q9KSE7	VC_1310	-1,1	3,2
Q9KTD3	cysZ	-1,1	3,6
Q9KPH0	ftsA	-1,1	3,2
Q9KP44	VC_2535	-1,1	3,6
Q9KRC6	mukF	-1,1	3,5
Q9F855	rluE	-1,1	3,4
Q9KP31	VC_2548	-1,1	3,4
Q9KP93	rpiA	-1,1	3,5
Q9KVC7	rpmG	-1,1	3,2
Q9KV62	VC_0295	-1,1	3,6
Q9KUC0	mrcB	-1,1	3,4
Q9KMQ5	VC_A0265	-1,1	2,9
H9L4R2	VC_1548	-1,1	2,8
Q9KQN9	minC	-1,1	3,2
Q9KRG4	VC_1678	-1,1	3,5
Q9KR88	VC_1754	-1,1	3,4
Q9KU33	VC_0696	-1,1	3,2
Q9KQV2	VC_1896	-1,1	1,8
Q9KNL0	cysQ	-1,1	2,8
Q9KS29	ttcA	-1,1	3,6
Q9KQX8	VC_1869	-1,1	3,3
Q9KU89	greA	-1,1	3,5
Q9KQ92	pdxB	-1,1	3,6
Q9KQN2	VC_1966	-1,1	3,1
Q9KN26	VC_A0140	-1,1	3,1
Q9KTA6	glnS	-1,1	3,6
Q9KNQ7	hslU	-1,1	3,4
Q9KQL1	VC_1987	-1,1	3,6
Q9KUI4	VC_0537	-1,1	3,6
Q9KV53	gppA	-1,1	3,6
Q9KRZ1	VC_1492	-1,1	3,6
Q9KVQ2	VC_0089	-1,1	3,5
Q9KVQ5	VC_0084	-1,1	2,9
Q9KM65	cqsA	-1,1	3,5
Q9KP17	VC_2562	-1,2	3,4
Q9KL53	VC_A0895	-1,2	2,4
Q9KPV6	uppS	-1,2	3,0
Q9KQE1	VC_2059	-1,2	3,6
Q9KT84	luxO	-1,2	3,3
Q9KPB0	lepA	-1,2	3,5
Q9KT81	moaA	-1,2	3,5

Q9KU70	VC_0653	-1,2	3,4
Q9KQD8	cheB1	-1,2	3,5
Q9KVS4	thiG	-1,2	3,5
Q9KVW8	glyS	-1,2	3,5
Q9KLK9	VC_A0734	-1,2	2,3
Q9KNM2	spoT	-1,2	3,0
Q9KRY4	VC_1499	-1,2	2,5
Q9KQ45	VC_2159	-1,2	3,5
Q9KVN0	engB	-1,2	3,3
Q9KS54	VC_1406	-1,2	3,0
Q9KP88	VC_2485	-1,2	2,7
Q9KVD1	coaBC	-1,2	3,4
Q9KTT1	VC_0807	-1,2	2,8
Q9KST7	trpA	-1,2	3,6
Q9KPA1	VC_2472	-1,2	3,5
Q9KMY7	VC_A0179	-1,2	3,4
Q9KUP2	tkt1	-1,2	3,7
Q9KS35	VC_1426	-1,2	3,6
Q9KVD8	VC_0208	-1,2	3,0
Q9KPK8	ung	-1,2	3,2
Q9KUW5	uvrA	-1,2	3,7
Q9KU79	rbfA	-1,2	3,1
Q9KUU7	VC_0418	-1,2	3,2
Q9KSY9	htpX	-1,2	3,5
Q9KTN0	VC_0872	-1,2	1,8
Q9KPC9	VC_2443	-1,2	3,6
Q9KT10	VC_1095	-1,2	3,4
Q9KV60	aroQ	-1,2	3,5
Q9KQA3	VC_2097	-1,2	3,3
Q9KU74	VC_0649	-1,2	3,3
Q9KUU1	rraB	-1,2	3,5
Q9KRS5	VC_1561	-1,2	2,8
Q9KU39	murQ2	-1,2	3,5
Q9KPY7	upp	-1,2	3,5
Q9KP73	valS	-1,2	3,7
Q9KM05	VC_A0584	-1,2	3,6
Q9KQK3	VC_1995	-1,2	2,7
Q9KV19	psd	-1,2	3,3
Q9KVL6	dapF	-1,2	2,8
Q9KKN0	VC_A1072	-1,2	2,6
Q9KTA3	accD	-1,2	3,6

Q9KPL8	deoA	-1,2	3,0
Q9KM27	VC_A0562	-1,2	3,7
Q9KUF5	VC_0566	-1,2	3,5
Q9KV93	VC_0263	-1,2	3,0
Q9KQ25	prfA	-1,2	3,6
Q9KNL4	bioH	-1,2	3,3
Q9KRU5	VC_1539	-1,2	3,4
Q9KUZ7	fusA1	-1,2	3,7
Q9KVW7	glyQ	-1,2	3,6
Q9KMZ1	VC_A0175	-1,2	3,2
P66367	rpsK	-1,2	3,6
Q06951	rfbB	-1,2	3,6
Q9KRY0	VC_1503	-1,2	3,4
Q9KSS2	VC_1184	-1,2	2,9
Q9KVM1	hemC	-1,2	3,5
Q9KV22	gpmI	-1,2	3,4
Q9KRU1	VC_1543	-1,2	3,6
Q9KT13	VC_1092	-1,2	2,1
Q9KRL4	nspC	-1,2	3,5
Q9KKP7	VC_A1055	-1,2	3,1
Q9KVV3	VC_0036	-1,2	3,5
Q9KR67	VC_1776	-1,2	2,1
Q9KSN0	tpm	-1,2	2,9
Q9KM69	VC_A0519	-1,2	3,2
Q9KUK1	rpoD	-1,2	3,7
Q9KTS5	VC_0813	-1,2	2,5
Q9KKM4	pdxH	-1,2	2,8
Q9KNP3	VC_2688	-1,2	3,4
Q9KLW8	tal	-1,2	3,7
O30862	grpE	-1,2	3,4
Q9KNT8	argG	-1,2	3,7
Q9KUM5	VC_0490	-1,2	3,0
Q9KP56	VC_2520	-1,2	3,6
Q9KTK3	VC_0899	-1,2	2,4
Q9KRB0	aroA	-1,2	3,1
Q9KUU9	VC_0416	-1,2	3,0
Q9KT90	rnfG	-1,2	3,5
Q9KNB0	VC_A0055	-1,2	2,9
Q9KTK4	xni	-1,2	3,5
Q9KRG7	VC_1675	-1,2	3,6
Q9KVY1	rpmH	-1,2	2,8

Q9KNH2	atpH	-1,2	3,6
Q9KTX1	ispG	-1,2	3,7
Q9KQR9	dctP	-1,2	3,5
Q9KSS7	rluB	-1,2	3,6
Q9KTY6	secF	-1,2	3,3
Q9KUS1	pdxA	-1,2	2,4
P0C6Q2	viuB	-1,2	3,5
Q9KTX0	hisS	-1,2	3,7
Q9KQM5	menB	-1,2	1,9
Q9KU15	VC_0715	-1,2	2,9
Q9KTX5	pepB	-1,2	3,5
Q9KLQ2	VC_A0690	-1,2	3,3
Q9KVN6	VC_0105	-1,2	3,7
Q9KUV4	VC_0408	-1,2	3,1
Q9KQ90	VC_2111	-1,2	3,3
Q9KNS1	efp	-1,2	3,7
Q9KLS9	VC_A0662	-1,2	3,4
Q9KM40	VC_A0549	-1,2	3,3
Q9KRS1	VC_1565	-1,2	3,6
Q9KNT0	gpsA	-1,2	3,7
Q9KT41	VC_1064	-1,2	3,6
Q9KTA4	truA	-1,2	1,6
Q9KSB7	VC_1341	-1,2	2,8
Q9KTD4	VC_0968	-1,2	3,5
Q9KTD1	ligA	-1,2	3,6
Q9KQH5	fabH1	-1,2	3,7
Q9KL24	pyrC	-1,2	3,5
Q9KV59	acsA	-1,2	3,5
Q9KV64	prmA	-1,2	3,3
Q9KNG5	rsmG	-1,2	3,7
Q9KVQ4	ubiB	-1,3	3,6
Q9KQ89	VC_2112	-1,3	3,2
Q9KNB7	VC_A0048	-1,3	3,2
Q9KSU2	cmoA	-1,3	3,1
Q9KQ05	VC_2202	-1,3	3,5
Q9KPI8	mtnN	-1,3	3,3
Q9KL32	VC_A0917	-1,3	3,3
Q9KQE2	VC_2058	-1,3	3,5
Q9KTS4	VC_0814	-1,3	3,3
Q9KPW3	fabZ	-1,3	1,6
Q9KQV3	VC_1895	-1,3	2,7

Q9KU41	VC_0688	-1,3	3,3
Q9KVB3	VC_0233	-1,3	2,0
Q9KQT8	VC_1910	-1,3	3,6
Q9KT66	VC_1039	-1,3	3,5
O34242	dnaJ	-1,3	2,8
Q9KSF0	VC_1306	-1,3	2,4
Q9KKR6	VC_A1035	-1,3	3,6
Q9KR11	tolB	-1,3	3,6
Q9KTK2	VC_0900	-1,3	3,6
Q9KT68	VC_1037	-1,3	3,7
P0C6Q1	gltX	-1,3	3,6
Q9KV87	VC_0269	-1,3	3,6
Q9KRW4	VC_1520	-1,3	3,6
Q9KLL1	VC_A0732	-1,3	2,9
P0C6Q9	bamE	-1,3	2,1
Q9KMK9	VC_A0330	-1,3	3,2
Q9KLT5	VC_A0656	-1,3	3,0
Q9KUP7	gshB	-1,3	3,2
Q9KLL4	VC_A0729	-1,3	3,1
Q9KMQ2	VC_A0268	-1,3	2,6
Q9KRY9	VC_1494	-1,3	3,6
Q9KMZ8	VC_A0168	-1,3	3,0
Q9KMP4	glyA2	-1,3	2,7
Q9KQ38	VC_2166	-1,3	3,5
Q9KT52	apt	-1,3	3,5
Q9KVB4	VC_0232	-1,3	2,9
Q9KLX9	mscL	-1,3	3,3
Q9KRW1	VC_1523	-1,3	3,0
Q9KP82	leuB	-1,3	3,0
Q9KND9	VC_A0026	-1,3	3,6
Q9KUW7	VC_0392	-1,3	3,5
Q9KTX2	VC_0758	-1,3	3,5
Q9KU34	VC_0695	-1,3	3,6
Q9KQD1	flhA	-1,3	2,9
Q9KPT4	VC_2278	-1,3	3,7
Q9KU11	VC_0719	-1,3	2,3
Q9KUJ7	plsY	-1,3	3,1
Q9KM14	VC_A0575	-1,3	3,7
Q9KTM0	VC_0882	-1,3	3,6
Q9KT37	VC_1068	-1,3	3,3
Q9KUV8	VC_0404	-1,3	2,8

Q9KNK3	VC_2735	-1,3	3,6
Q9KSU8	VC_1158	-1,3	3,4
Q9KU12	rdgC	-1,3	3,7
Q9KQU9	VC_1899	-1,3	3,4
Q9KRJ1	VC_1649	-1,3	3,2
Q9KNW0	rimO	-1,3	3,7
Q9KLE4	VC_A0802	-1,3	3,4
Q9KP47	VC_2532	-1,3	2,5
Q9KTY0	VC_0750	-1,3	3,5
Q9KP24	VC_2555	-1,3	2,8
Q9KQ40	VC_2164	-1,3	3,7
Q9KL55	VC_A0893	-1,3	3,4
Q9KPJ0	VC_2377	-1,3	2,5
Q9KSN9	VC_1217	-1,3	2,9
Q9KSF6	VC_1300	-1,3	3,6
Q9KT56	VC_1049	-1,3	2,8
Q9KMZ9	VC_A0167	-1,3	3,6
Q9KUN5	VC_0480	-1,3	3,5
Q9KNC8	VC_A0037	-1,3	3,6
Q9KS84	VC_1375	-1,3	3,7
Q9K344	VC_0256	-1,3	2,6
Q9KSJ9	ubiG	-1,3	3,2
Q9KRH2	clsA	-1,3	3,0
Q9KUQ6	VC_0459	-1,3	3,5
P57070	lolB	-1,3	2,8
Q9KQK0	msrB	-1,3	3,5
Q9KU44	ispH	-1,3	3,7
Q9KNU3	VC_2637	-1,3	3,5
Q9KTL3	dxs	-1,3	3,6
Q9KTF4	rlpA	-1,3	2,8
Q9KR13	cpoB	-1,3	3,6
Q9KSK7	VC_1249	-1,3	3,6
Q9KPX5	VC_2237	-1,3	3,5
Q9KLW7	tkt2	-1,3	3,7
P32557	dsbA	-1,3	3,7
H9L4P6	VC_2136	-1,3	1,9
Q9KUK2	VC_0516	-1,3	2,6
Q9KPM6	VC_2341	-1,3	3,7
Q9KU99	VC_0624	-1,3	3,1
Q9KV28	VC_0330	-1,3	3,5
Q9KNR6	groS1	-1,3	3,0

Q9KRG5	VC_1677	-1,3	2,8
Q9KRB6	glgA	-1,3	3,5
Q9KL57	VC_A0891	-1,3	2,4
Q9KN86	VC_A0079	-1,4	2,7
Q9KU59	VC_0665	-1,4	3,4
Q9KVX8	VC_0010	-1,4	2,5
Q9KP49	VC_2529	-1,4	2,6
H9L4Q9	VC_2451	-1,4	3,7
Q9KLQ1	VC_A0691	-1,4	3,1
Q9KL87	VC_A0859	-1,4	3,4
Q9KSW1	VC_1145	-1,4	3,7
Q9KVD4	lpxM	-1,4	2,4
Q9KMM3	VC_A0308	-1,4	3,5
P57069	lolA	-1,4	3,6
Q9KS71	lplA	-1,4	3,7
Q9KKJ4	VC_A1113	-1,4	3,5
Q9KM22	VC_A0567	-1,4	2,4
Q9KVG0	VC_0186	-1,4	3,5
Q9KQP1	VC_1957	-1,4	1,7
Q9KNN7	VC_2694	-1,4	3,6
Q9KQP2	VC_1956	-1,4	3,6
Q9KSS6	VC_1180	-1,4	3,1
Q9KQB1	VC_2089	-1,4	3,7
Q9KTF7	VC_0945	-1,4	3,6
Q9KLF8	VC_A0786	-1,4	3,6
Q9KPZ0	VC_2222	-1,4	3,7
Q9KRQ8	VC_1578	-1,4	3,2
Q9KUB5	VC_0608	-1,4	3,6
Q9KPF3	VC_2415	-1,4	3,5
Q9KTI9	VC_0913	-1,4	2,3
Q9KNG9	atpB	-1,4	3,6
Q9KNN1	dsbD	-1,4	3,4
Q9KU93	VC_0630	-1,4	3,3
Q9KQZ8	VC_1849	-1,4	3,4
Q9KQ59	VC_2145	-1,4	3,2
Q9KU48	VC_0681	-1,4	3,0
Q9KUS8	cgtA	-1,4	3,8
Q9KU78	truB	-1,4	3,6
Q9KL70	VC_A0877	-1,4	3,4
Q9KPF1	VC_2417	-1,4	2,4
Q9KVA0	VC_0250	-1,4	3,7

O66108	thyA	-1,4	3,7
Q9KP71	rluA	-1,4	3,6
Q9KNC5	VC_A0040	-1,4	3,5
Q9KV18	rsgA	-1,4	3,4
Q9KTC5	VC_0977	-1,4	3,1
Q9KTL2	VC_0890	-1,4	2,7
Q9KPH2	lpxC	-1,4	3,0
H9L4Q3	VC_1415	-1,4	1,8
Q9KRS3	VC_1563	-1,4	3,1
Q9KMZ4	VC_A0172	-1,4	3,6
Q9KPX2	padC	-1,4	3,5
P0C6D3	vibB	-1,4	2,8
Q9KTW6	VC_0764	-1,4	3,7
Q9KNV5	VC_2625	-1,4	3,6
Q9KVR6	rsmJ	-1,4	3,7
Q9KM03	VC_A0586	-1,4	2,7
Q9KUG7	VC_0554	-1,4	3,6
P58093	parD	-1,4	2,8
Q9KN66	VC_A0099	-1,4	3,7
Q9KM94	VC_A0487	-1,4	3,0
Q9KTS3	VC_0815	-1,4	3,3
Q9KVH5	VC_0171	-1,4	3,7
Q9KSP2	uvrC	-1,4	3,5
Q9KVT4	hemF	-1,4	3,5
Q9KVD6	rph	-1,4	3,7
Q9KR02	ruvB	-1,4	3,0
Q9KR64	siaP	-1,4	3,4
Q9KTJ5	metN	-1,4	2,3
Q9KT18	VC_1087	-1,4	1,4
P0C6Q4	recN	-1,4	2,9
Q9KUM3	VC_0492	-1,4	3,7
Q9KS50	VC_1410	-1,4	3,2
Q9KQF0	VC_2048	-1,4	2,5
Q9KTX8	hscA	-1,4	3,7
Q9KQX4	VC_1873	-1,5	3,7
Q9KR07	VC_1840	-1,5	3,2
Q9KQD4	fliA	-1,5	3,5
Q9KKN5	VC_A1067	-1,5	3,5
Q9KTA2	VC_1001	-1,5	2,4
Q9KNP7	VC_2684	-1,5	3,7
Q9KRN0	VC_1606	-1,5	2,8

Q9KQW4	VC_1884	-1,5	2,8
Q9KNC2	VC_A0043	-1,5	3,6
Q9KP02	rpmD	-1,5	3,7
Q9KL20	rlmC	-1,5	2,5
Q9KP81	leuC	-1,5	3,4
Q9KSZ4	bioB	-1,5	3,7
H9L4R1	VC_0412	-1,5	2,8
Q9KKZ9	VC_A0951	-1,5	3,7
Q9KTI8	VC_0914	-1,5	3,5
Q9KPS9	VC_2283	-1,5	1,8
Q9KL26	VC_A0923	-1,5	3,7
Q9KPS7	VC_2285	-1,5	2,7
Q9KM50	VC_A0539	-1,5	3,5
Q9KNS6	epmA	-1,5	3,5
H9L4Q7	VC_2528	-1,5	3,7
Q9KR87	VC_1755	-1,5	2,3
Q9KVM6	VC_0115	-1,5	3,6
Q9KV72	VC_0285	-1,5	3,5
Q9KQW9	msbA	-1,5	3,6
Q9KTM9	VC_0873	-1,5	3,1
Q9KRK2	VC_1638	-1,5	3,7
Q9KLC6	groL2	-1,5	2,5
Q9KMP3	VC_A0279	-1,5	3,4
Q9KM21	VC_A0568	-1,5	2,7
Q9KRL5	VC_1622	-1,5	3,6
Q9KLI5	VC_A0759	-1,5	2,3
Q9KQ74	VC_2127	-1,5	3,7
Q9KNT2	VC_2649	-1,5	3,2
Q9KKR8	VC_A1033	-1,5	3,8
Q9KPQ7	csd	-1,5	3,4
Q9KTM4	rpmE2	-1,5	3,2
Q9KLU7	VC_A0644	-1,5	3,4
Q9KRS6	katG	-1,5	3,8
Q9KRB5	glgC1	-1,5	3,5
Q9KS51	VC_1409	-1,5	3,2
Q9KNA7	VC_A0058	-1,5	3,5
Q9KLH1	VC_A0773	-1,5	3,3
Q9KU08	ppx	-1,5	3,6
Q9KTE9	VC_0953	-1,5	2,6
Q9KQX5	VC_1872	-1,5	3,7
Q9KU00	cutC	-1,5	2,9

Q9KLX3	VC_A0618	-1,5	3,3
Q9KP98	VC_2475	-1,5	2,9
Q9KPQ5	VC_2311	-1,6	3,2
Q9KUN2	VC_0483	-1,6	3,8
Q9KSG8	opgG	-1,6	3,7
Q9KTX4	ndk	-1,6	3,7
Q9KRI3	VC_1658	-1,6	2,6
Q9KRJ7	VC_1643	-1,6	3,7
P45777	epsC	-1,6	3,0
Q9KQ71	VC_2130	-1,6	3,0
Q9KKR9	VC_A1032	-1,6	3,7
Q9KQI5	VC_2013	-1,6	3,8
Q9KM74	VC_A0514	-1,6	3,6
Q9KVK4	VC_0139	-1,6	3,6
Q9KVJ0	trmA	-1,6	3,7
Q9KUC6	dksA	-1,6	3,9
Q9KNV1	aroK	-1,6	3,8
Q9KUW8	VC_0391	-1,6	3,6
Q9KSG9	opgH	-1,6	3,7
Q9KPD5	VC_2435	-1,6	3,7
Q9KQC2	VC_2078	-1,6	3,4
Q9KUQ2	VC_0463	-1,6	3,6
Q9KP61	VC_2515	-1,6	3,7
Q9KU52	VC_0672	-1,6	2,3
Q9KSD0	VC_1328	-1,6	3,4
Q9KPN3	VC_2333	-1,6	1,9
Q9KQF4	VC_2044	-1,6	3,4
Q9KS38	VC_1423	-1,6	3,8
Q9KNK2	hslO	-1,6	3,5
Q9KKZ4	vdcA	-1,6	1,6
Q9KN27	VC_A0139	-1,6	3,6
Q9KUD5	VC_0587	-1,6	2,9
Q9F854	hisD	-1,6	3,3
Q9KTC1	VC_0982	-1,6	3,6
Q9KL64	VC_A0883	-1,6	3,3
Q9KUU5	VC_0420	-1,6	3,4
Q9KTJ6	metI	-1,6	3,2
Q9KUM2	VC_0493	-1,6	3,1
Q9KQ76	VC_2125	-1,6	3,7
Q9KQX3	VC_1874	-1,6	3,8
Q9KLM6	VC_A0717	-1,6	3,0

Q9KTH8	VC_0924	-1,6	3,6
Q9KRL3	VC_1624	-1,6	3,6
Q9KMT9	VC_A0230	-1,7	3,6
Q9KV12	miaA	-1,7	3,4
Q9KVG8	capV	-1,7	3,6
Q9KSQ4	hutH	-1,7	3,0
Q9KL42	VC_A0906	-1,7	3,4
Q9KUX0	VC_0389	-1,7	3,7
Q9KTN8	VC_0863	-1,7	2,3
Q9KVL4	xerC	-1,7	3,9
Q9KKJ5	tmcA	-1,7	3,8
Q9KQZ0	VC_1857	-1,7	3,3
Q9KQE7	ccmE	-1,7	3,3
O07898	vibC	-1,7	3,7
Q9KTM1	VC_0881	-1,7	3,7
Q9KMC1	VC_A0443	-1,7	3,4
Q9KQ83	prmB	-1,7	3,6
Q9KPF0	VC_2418	-1,7	3,8
Q9KPZ9	VC_2208	-1,7	3,1
Q9KVU4	fmt	-1,7	3,7
Q9KKR7	VC_A1034	-1,7	3,8
Q9KUR6	VC_0449	-1,7	3,5
Q9KLC9	VC_A0817	-1,7	3,9
Q9KLB6	VC_A0830	-1,7	2,6
Q9KV17	orn	-1,7	3,7
Q9KVY6	mioC	-1,7	2,4
Q9KQZ4	VC_1853	-1,7	3,8
Q9KM95	VC_A0486	-1,7	3,8
Q9KS95	VC_1364	-1,7	2,2
Q9KVC4	coaD	-1,7	3,1
Q9KQE0	VC_2060	-1,7	2,9
Q9KUI0	cysA	-1,7	3,5
Q9KKL2	VC_A1092	-1,7	2,9
Q9KU32	VC_0697	-1,7	3,3
Q9KLD5	gbpA	-1,7	2,9
Q9KT58	fadJ	-1,7	3,7
Q9KRE5	VC_1697	-1,7	3,6
Q9KR77	VC_1766	-1,7	3,7
Q9KPV0	glnD	-1,7	3,9
Q9KM19	VC_A0570	-1,7	3,8
Q9KL02	VC_A0948	-1,7	3,5

Q9KVC6	VC_0220	-1,7	2,9
P37093	epsE	-1,8	3,7
Q9KU37	nagZ	-1,8	3,6
Q9KSJ3	ribA	-1,8	3,8
Q9KUB1	VC_0612	-1,8	3,5
Q9KRX5	VC_1508	-1,8	3,8
Q9KL48	VC_A0900	-1,8	3,8
Q9KNM1	recG	-1,8	3,7
Q9KQT6	lapB	-1,8	2,8
Q9KR91	VC_1751	-1,8	3,1
Q9KLX1	VC_A0620	-1,8	3,6
Q9KP57	VC_2519	-1,8	3,6
Q9KPG8	murC	-1,8	3,7
Q9KTD9	VC_0963	-1,8	2,9
Q9KPW4	lpxA	-1,8	3,8
Q9KTE5	VC_0957	-1,8	3,4
Q9KVC5	mutM	-1,8	3,4
O34238	murJ	-1,8	3,4
Q9KUV0	VC_0414	-1,8	2,5
Q9KPZ5	VC_2217	-1,8	3,0
Q9KR75	VC_1768	-1,8	3,9
Q9KV74	VC_0282	-1,8	2,5
Q9KMZ2	VC_A0174	-1,8	3,2
Q9KSZ3	bioF	-1,9	3,8
Q9KUS4	apaH	-1,9	3,7
Q9KTT5	VC_0803	-1,9	3,3
Q9KRA2	VC_1740	-1,9	2,8
Q9KLQ7	VC_A0685	-1,9	2,7
Q9KQ24	hemA	-1,9	3,8
Q9KMZ7	VC_A0169	-1,9	3,3
H9L4S8	VC_1344	-1,9	3,4
Q9KSI4	VC_1272	-1,9	2,6
Q9KP76	VC_2499	-1,9	2,4
Q9KUB9	VC_0603	-1,9	3,0
Q9KMW8	VC_A0198	-1,9	2,8
Q9KSG0	VC_1296	-1,9	2,9
Q9KPX6	gloB	-1,9	2,5
Q9KNM9	nhaP2	-1,9	3,3
Q9KPU8	VC_2264	-1,9	3,5
Q9KPU0	nrdR	-1,9	4,0
Q9KNL1	VC_2721	-1,9	3,2

Q9KLC7	groS2	-1,9	3,2
Q9KMA5	higA-2	-1,9	3,8
Q9KR14	nadA	-1,9	3,7
Q9KQW3	VC_1885	-1,9	2,8
Q9KLY4	VC_A0607	-1,9	3,3
Q9KQC5	VC_2075	-2,0	3,5
Q9KLZ8	VC_A0591	-2,0	3,7
Q9KPR4	VC_2302	-2,0	2,8
Q9KMW4	rhlE	-2,0	3,0
Q9KKY8	VC_A0962	-2,0	2,9
Q9KMZ5	VC_A0171	-2,0	3,7
P0C6P8	VC_0850	-2,0	3,4
Q9KPA8	VC_2465	-2,0	3,8
Q9KUR2	trmB	-2,0	3,2
Q9KLP4	glgC2	-2,0	3,5
Q9KL03	speG	-2,0	3,1
Q9KUW9	metH	-2,0	4,0
H9L4P1	VC_0259	-2,0	3,7
Q9KT61	VC_1044	-2,0	3,5
Q9KQH2	VC_2026	-2,0	3,4
Q9KTL0	VC_0892	-2,0	3,5
Q9KLS8	VC_A0663	-2,0	3,5
Q9KND6	VC_A0029	-2,0	3,8
Q9KRR3	fumC	-2,0	3,9
Q9KQM3	menD	-2,0	3,7
Q9KQ44	VC_2160	-2,0	2,7
Q9KRT2	VC_1554	-2,1	2,6
Q9KRS9	VC_1557	-2,1	3,6
Q9KPP2	VC_2324	-2,1	2,6
Q9KPP4	recC	-2,1	3,8
Q9KN45	VC_A0120	-2,1	3,0
Q9KMP5	gcvH	-2,1	3,3
P45782	epsL	-2,1	3,2
Q9KS82	VC_1377	-2,1	3,6
P0C6Q5	tcpF	-2,1	3,7
Q9KPV1	map	-2,1	2,4
Q9KPH6	VC_2392	-2,1	3,7
Q9KQZ6	VC_1851	-2,1	3,9
Q9KUA3	VC_0620	-2,1	3,8
Q9KUD3	VC_0589	-2,1	3,5
Q9KU03	VC_0727	-2,1	3,6

Q9KVQ7	rmuC	-2,1	3,9
Q9KUL9	VC_0496	-2,1	2,9
Q9KUD9	rsmI	-2,1	4,0
Q9KT95	VC_1008	-2,1	3,2
Q9KS57	VC_1403	-2,1	3,6
Q9KSC4	VC_1334	-2,1	3,6
Q9KT59	fadI	-2,1	3,7
Q9KN35	VC_A0130	-2,2	3,1
P0C6C2	epd	-2,2	3,0
Q9KN88	VC_A0077	-2,2	3,8
Q9KUW0	VC_0399	-2,2	2,3
Q9KMD8	VC_A0423	-2,2	3,5
Q9KSG7	VC_1289	-2,2	3,8
Q9KLL7	VC_A0726	-2,2	3,4
Q9KPM3	VC_2344	-2,2	3,9
Q9KU24	VC_0705	-2,2	3,6
Q9KTY4	trmJ	-2,2	3,6
Q9KTK1	VC_0901	-2,2	3,7
Q9KUC7	gluQ	-2,2	4,0
Q9KTZ4	VC_0736	-2,2	3,9
Q9KVA1	VC_0248	-2,2	3,7
Q9KPJ1	VC_2376	-2,2	2,6
Q9KPI9	VC_2378	-2,2	3,7
Q9KPS0	bolA	-2,2	3,0
Q9KVC1	VC_0225	-2,2	2,4
Q9KV14	VC_0344	-2,3	3,3
Q9KMZ6	VC_A0170	-2,3	3,4
Q9KVH2	VC_0174	-2,3	3,5
Q9KTV6	VC_0781	-2,3	3,5
Q9KNI1	fadB	-2,3	2,5
Q9KSS8	VC_1178	-2,3	3,6
Q9KVW1	ilvA	-2,3	3,6
Q9KTW4	xseA	-2,3	3,9
Q9KKM1	VC_A1082	-2,3	3,4
Q9KUQ3	VC_0462	-2,3	2,6
Q9KKT9	VC_A1011	-2,3	3,5
Q9KKX2	VC_A0978	-2,3	3,6
Q9KTK9	VC_0893	-2,3	3,9
Q9KT93	gloA	-2,3	4,0
Q9KPD2	VC_2440	-2,3	3,5
Q9KPC7	VC_2445	-2,3	3,5

Q9ZHV9	exbD2	-2,3	3,6
Q9KMM4	VC_A0307	-2,3	3,4
Q9KQL4	rnd	-2,4	2,5
Q9KKS5	nagB	-2,4	4,1
Q9KQU8	fadR	-2,4	4,2
Q9KKL8	VC_A1085	-2,4	3,0
Q9KMU4	VC_A0225	-2,4	4,0
Q9KQC3	VC_2077	-2,4	3,8
Q9KT44	VC_1061	-2,4	3,6
Q9KRY2	VC_1501	-2,4	2,5
Q9KQC7	VC_2073	-2,4	3,0
Q9KQM2	menF	-2,4	2,2
Q9KVY0	VC_0008	-2,4	3,9
Q9KLV4	VC_A0637	-2,4	3,9
Q9KVW9	VC_0019	-2,4	4,1
Q9KPW5	lpxB	-2,4	3,1
Q9KQC8	VC_2072	-2,4	3,7
Q9KT65	VC_1040	-2,5	3,5
Q9KRY1	rsmF	-2,5	2,8
Q9KKW9	VC_A0981	-2,5	3,4
Q9KPC0	VC_2453	-2,5	2,4
Q9X6N4	murE	-2,5	3,6
Q9KPS6	VC_2286	-2,5	3,3
Q9KQD0	VC_2070	-2,5	4,0
Q9KQ23	ispE	-2,5	3,8
Q9KMY4	VC_A0182	-2,5	4,1
Q9KS17	VC_1444	-2,6	3,9
Q9KSR0	VC_1196	-2,6	4,0
Q9KSQ8	VC_1198	-2,6	3,9
Q9KNL8	VC_2714	-2,7	3,4
Q9KLT7	VC_A0654	-2,7	4,0
Q9KSV4	VC_1152	-2,8	2,6
Q9KUU2	arcA	-2,8	3,7
Q9KTL6	VC_0886	-2,8	3,9
Q9KQX9	VC_1868	-2,8	4,1
Q9KKQ5	VC_A1047	-2,8	3,2
Q9KLK2	VC_A0741	-2,8	2,6
Q9KTW8	bamB	-2,9	3,1
Q9KMW5	VC_A0201	-2,9	3,8
Q9KPK4	thrB	-2,9	3,0
Q9KVU3	def1	-3,0	2,8

Q9KS12	rtxA	-3,0	4,0
Q9KP22	VC_2557	-3,1	2,6
Q9KMA6	higB-2	-3,1	3,5
Q9KSB3	VC_1346	-3,1	3,7
Q9KTB3	rfaH	-3,1	3,8
Q9KSU7	serC	-3,2	3,1
Q9KRK3	VC_1637	-3,5	3,5
Q56632	vibA	-3,5	4,5
Q9KM66	cqsS	-3,6	3,8
Q9KS88	VC_1371	-3,6	3,7
Q9KLT3	VC_A0658	-3,6	2,3
Q9KQ11	VC_2196	-3,8	4,4
Q9KS11	VC_1453	-4,0	4,5
Q9KQF5	topB	-4,2	1,9
Q9KM24	VC_A0565	-4,3	4,0
Q9KVJ2	VC_0152	-4,9	4,1
Q9KRD9	VC_1703	-5,1	2,9
O85187	nhaA	absent in dusB	
P0C6D8	tcpI	absent in dusB	
P45781	epsK	absent in dusB	
Q9K2P2	VC_A0292	absent in dusB	
Q9KKL6	VC_A1087	absent in dusB	
Q9KKR5	VC_A1037	absent in dusB	
Q9KL01	VC_A0949	absent in dusB	
Q9KLA0	VC_A0846	absent in dusB	
Q9KLB3	VC_A0833	absent in dusB	
Q9KLB7	VC_A0829	absent in dusB	
Q9KLK5	VC_A0738	absent in dusB	
Q9KLM3	VC_A0720	absent in dusB	
Q9KLN4	VC_A0709	absent in dusB	
Q9KLQ3	VC_A0689	absent in dusB	
Q9KM04	VC_A0585	absent in dusB	
Q9KM07	VC_A0582	absent in dusB	
Q9KM08	VC_A0581	absent in dusB	
Q9KM28	VC_A0561	absent in dusB	
Q9KMA2	VC_A0475	absent in dusB	
Q9KMD9	VC_A0422	absent in dusB	
Q9KMQ7	VC_A0263	absent in dusB	
Q9KN07	VC_A0159	absent in dusB	
Q9KN40	VC_A0125	absent in dusB	
Q9KN50	VC_A0115	absent in dusB	

Q9KN51	VC_A0114	absent in dusB	
Q9KNI0	fadA	absent in dusB	
Q9KNW1	VC_2619	absent in dusB	
Q9KNW3	VC_2617	absent in dusB	
Q9KPC6	cca	absent in dusB	
Q9KPN1	VC_2335	absent in dusB	
Q9KPY0	VC_2232	absent in dusB	
Q9KPY1	VC_2231	absent in dusB	
Q9KQ09	VC_2198	absent in dusB	
Q9KQ17	VC_2190	absent in dusB	
Q9KQ28	VC_2176	absent in dusB	
Q9KQ63	fliD	absent in dusB	
Q9KQK5	VC_1993	absent in dusB	
Q9KQQ1	VC_1947	absent in dusB	
Q9KQR3	VC_1935	absent in dusB	
Q9KR15	VC_1832	absent in dusB	
Q9KR62	nanE	absent in dusB	
Q9KRD8	metE	absent in dusB	
Q9KRG3	VC_1679	absent in dusB	
Q9KRG8	VC_1674	absent in dusB	
Q9KRI8	VC_1653	absent in dusB	
Q9KRJ8	VC_1642	absent in dusB	
Q9KRX9	VC_1504	absent in dusB	
Q9KS18	VC_1443	absent in dusB	
Q9KSE2	VC_1315	absent in dusB	
Q9KSL1	btuD	absent in dusB	
Q9KSS9	VC_1177	absent in dusB	
Q9KSX5	VC_1131	absent in dusB	
Q9KT09	VC_1096	absent in dusB	
Q9KT78	VC_1027	absent in dusB	
Q9KT87	rnfB	absent in dusB	
Q9KT97	rnt	absent in dusB	
Q9KTR8	VC_0822	absent in dusB	
Q9KTZ6	VC_0734	absent in dusB	
Q9KU36	VC_0693	absent in dusB	
Q9KU45	VC_0684	absent in dusB	
Q9KU53	rppH	absent in dusB	
Q9KU62	VC_0661	absent in dusB	
Q9KU66	VC_0657	absent in dusB	
Q9KUG2	VC_0559	absent in dusB	
Q9KUH9	VC_0542	absent in dusB	

Q9KUJ4	uppP	absent in dusB	
Q9KUK3	VC_0515	absent in dusB	
Q9KUN3	argP	absent in dusB	
Q9KUX2	cysH	absent in dusB	
Q9KV05	VC_0353	absent in dusB	
Q9KV66	dusB	absent in dusB	
Q9KV75	VC_0281	absent in dusB	
Q9KV79	VC_0277	absent in dusB	
Q9KVF2	VC_0194	absent in dusB	
Q9KVG9	vspR	absent in dusB	
Q9KVI8	VC_0157	absent in dusB	
Q9KVL3	VC_0129	absent in dusB	
Q9KVL9	VC_0122	absent in dusB	
Q9KVS1	VC_0068	absent in dusB	
Q9KVU7	VC_0042	absent in dusB	
Q9KVV4	srkA	absent in dusB	
Q9KVX4	recF	absent in dusB	
ΔdusB pm6 vs pDusB+ H2O2 (195up, 124 down)			
Uniprot accession	Gene name	m6/wt	neglogpadj
Q9KTR8	VC_0822	5,3	5,1
Q9KSB0	VC_1349	5,2	4,2
Q9KVV8	VC_0030	4,9	4,8
Q9KUM9	VC_0486	4,8	4,0
O32445	nagA	4,0	3,0
Q9KM04	VC_A0585	4,0	2,8
O85187	nhaA	4,0	2,7
Q9KM28	VC_A0561	4,0	2,6
Q9K2P2	VC_A0292	4,0	2,5
Q9KMY4	VC_A0182	4,0	2,5
Q9KKR5	VC_A1037	4,0	2,4
Q9KN07	VC_A0159	4,0	2,3
Q9KKV2	VC_A0998	4,0	2,3
Q9KN26	VC_A0140	4,0	2,3
Q9KL01	VC_A0949	4,0	2,2
Q9KPC6	cca	4,0	2,2
Q9KLB3	VC_A0833	4,0	2,2
Q9KQ28	VC_2176	4,0	2,1
Q9KLH7	VC_A0767	4,0	2,1
Q9KSD0	VC_1328	4,0	2,1

Q9KLK5	VC_A0738	4,0	2,0
Q9KSS9	VC_1177	4,0	2,0
Q9KLY1	VC_A0610	4,0	2,0
Q9KT87	rnfB	4,0	2,0
Q9KTA0	VC_1003	4,0	1,9
Q9KTS9	VC_0809	4,0	1,9
Q9KU45	VC_0684	4,0	1,9
Q9KUI0	cysA	4,0	1,8
Q9KUI9	surE	4,0	1,8
Q9KV75	VC_0281	4,0	1,8
Q9KVG9	vspR	4,0	1,8
Q9KVS1	VC_0068	4,0	1,7
Q9KQV6	VC_1892	3,9	3,0
Q9KSY4	VC_1122	3,8	3,6
Q9KMX2	VC_A0194	3,8	3,7
Q9KSZ2	bioC	3,8	1,7
Q9KPZ5	VC_2217	3,4	2,2
Q9KP97	VC_2476	3,3	3,1
Q9KQ02	VC_2205	3,0	1,9
Q9KQT6	lapB	2,9	3,2
Q9KQT4	ihfB	2,8	2,5
Q9KSK9	VC_1247	2,7	1,9
Q9KQL5	VC_1983	2,7	2,8
Q9KQF0	VC_2048	2,7	2,6
Q9KNA0	VC_A0065	2,6	2,4
Q9KV15	VC_0343	2,6	3,1
Q9KST9	tdk	2,6	3,2
Q9KQY2	VC_1865	2,5	1,7
Q9KPK9	VC_2358	2,5	2,6
Q9KRY2	VC_1501	2,4	1,8
B1B1N2	VC_A0594	2,4	2,9
Q9KKM5	VC_A1078	2,3	3,1
Q9KRV7	VC_1527	2,3	2,3
Q9KVL2	cdpA	2,2	3,2
Q9KSL8	cobS	2,2	3,6
Q9KRX9	VC_1504	2,2	2,4
Q9KQG7	VC_2031	2,2	2,1
Q9KRK7	VC_1633	2,2	2,3
Q9KR64	siaP	2,2	2,9
Q9KPK7	nfo	2,2	3,6
Q9KSE9	VC_1308	2,2	2,4

Q9KLA1	VC_A0845	2,1	2,6
Q9KN79	VC_A0086	2,1	2,3
Q9KTY8	yajC	2,1	2,9
Q9KSK6	VC_1250	2,1	3,2
Q9KNN5	VC_2696	2,1	2,4
Q9KM44	VC_A0545	2,1	2,7
Q9KLY3	VC_A0608	2,0	1,3
Q9KPA9	VC_2464	2,0	2,4
Q9KS75	VC_1384	2,0	1,3
Q9KPD0	VC_2442	2,0	2,1
Q9KMQ2	VC_A0268	2,0	2,6
Q9KUB7	VC_0605	1,9	1,8
Q9KVR3	VC_0076	1,9	2,1
Q9KSS0	VC_1186	1,9	2,6
Q9KQ73	VC_2128	1,9	2,4
Q9KRL1	VC_1629	1,9	2,5
Q9KPC0	VC_2453	1,9	2,3
Q9KRJ8	VC_1642	1,9	1,8
Q9KKR3	VC_A1039	1,9	1,9
Q9KPJ4	VC_2373	1,8	1,8
Q9KN58	VC_A0107	1,8	1,4
P52695	hlyU	1,8	1,7
Q9KRM5	metAS	1,8	1,5
Q9KPY3	VC_2229	1,8	2,2
Q9KQX6	VC_1871	1,8	2,1
Q9KVM5	VC_0116	1,8	1,8
Q9KVB0	VC_0236	1,7	2,3
Q9KSF0	VC_1306	1,7	2,8
Q9KNA2	VC_A0063	1,7	3,3
Q9KQX1	VC_1876	1,7	3,2
Q9KUB6	VC_0606	1,7	2,1
Q9KLN2	mgsA	1,7	1,7
Q9KRN4	VC_1602	1,6	1,8
Q9KQE9	dsbE	1,6	1,5
Q9KM35	VC_A0554	1,6	2,3
Q9KL63	VC_A0884	1,6	1,5
Q9KSZ8	VC_1108	1,6	1,9
Q9KT45	VC_1060	1,6	1,7
Q9KM37	VC_A0552	1,6	2,4
Q9KNQ9	rraA	1,6	1,6
Q9KLS6	dcuC	1,6	1,8

Q9KSE0	VC_1317	1,6	1,6
Q9KR62	nanE	1,6	1,5
Q9KTV3	VC_0784	1,6	1,4
Q9KPJ3	VC_2374	1,5	1,8
Q9KRX2	VC_1511	1,5	2,4
Q9KVE6	VC_0200	1,5	2,8
Q9KUU0	VC_0425	1,5	1,9
Q9KMQ0	VC_A0270	1,5	2,0
Q9KNM4	gmk	1,5	1,9
Q9KRD1	VC_1711	1,5	1,9
Q9KNS5	VC_2656	1,5	2,2
Q9KMJ9	VC_A0344	1,4	2,1
Q9KS73	VC_1386	1,4	1,8
Q9KP69	VC_2507	1,4	2,1
Q9KM84	VC_A0498	1,4	2,5
Q9KPP2	VC_2324	1,4	1,4
Q9KKL2	VC_A1092	1,4	1,8
Q9KQJ2	VC_2006	1,4	1,8
Q9KVU8	VC_0041	1,4	1,8
Q9KRH1	VC_1671	1,4	1,6
Q9KU23	VC_0706	1,4	1,6
Q9KQG3	VC_2035	1,4	1,8
Q9KR02	ruvB	1,4	2,3
Q9KTN5	mltF	1,4	2,3
Q9KP99	VC_2474	1,4	1,6
Q9KTN0	VC_0872	1,4	1,3
Q9KPK6	grcA	1,3	2,1
Q9KT86	rnfA	1,3	2,3
Q9KS51	VC_1409	1,3	1,5
Q9KU07	ppk	1,3	2,7
Q9KM78	VC_A0510	1,3	1,5
Q9KPT6	VC_2276	1,3	1,6
Q9KU16	VC_0714	1,3	1,9
Q9KTK2	VC_0900	1,3	2,6
Q9KVS3	VC_0066	1,3	2,5
Q9KRC0	VC_1722	1,3	2,0
Q9KPX6	gloB	1,3	1,4
Q9KKU4	VC_A1006	1,3	1,3
Q9KSX1	hisB	1,3	2,0
Q9KRN6	VC_1600	1,3	1,5
Q9KN53	VC_A0112	1,3	1,4

Q9KVW0	ilvD	1,3	2,6
Q9KU42	VC_0687	1,3	2,0
Q9KQV5	VC_1893	1,3	2,1
Q9KTV6	VC_0781	1,3	2,0
Q9KQG5	VC_2033	1,2	2,0
Q9KPU6	thiL	1,2	1,5
Q9KQN5	VC_1963	1,2	1,8
Q9KV43	VC_0315	1,2	2,3
Q9KL13	VC_A0936	1,2	2,6
Q9KKP7	VC_A1055	1,2	2,2
Q9KS01	VC_1482	1,2	2,4
Q9KVK6	cdgJ	1,2	2,1
P0C6C2	epd	1,2	2,7
Q9KN51	VC_A0114	1,2	1,9
Q9KTJ5	metN	1,2	1,5
Q9KUF9	rimM	1,2	1,7
O07899	vibE	1,2	1,9
Q9KKW0	VC_A0990	1,2	2,0
Q9KQ26	prmC	1,2	1,3
Q9KNF1	VC_A0013	1,1	1,9
Q9KQ32	VC_2172	1,1	2,8
Q9KNX0	VC_2610	1,1	1,7
Q9KQ88	VC_2113	1,1	1,6
Q9KNC3	VC_A0042	1,1	2,4
Q9KRY4	VC_1499	1,1	1,7
Q9KRI6	VC_1655	1,1	1,4
Q9KU85	VC_0638	1,1	1,5
Q9KSI6	VC_1270	1,1	1,6
Q9KQ70	VC_2131	1,1	2,3
Q9KUR8	djlA	1,1	1,5
Q9KNM3	rpoZ	1,1	2,5
Q9KL86	VC_A0860	1,1	1,9
Q9KS86	VC_1373	1,1	2,2
Q9KR83	VC_1760	1,1	2,2
Q9KQL4	rnd	1,0	1,5
Q9KRF8	VC_1684	1,0	1,6
Q9KKZ6	VC_A0954	1,0	1,5
Q9KLT2	VC_A0659	1,0	1,7
Q9KT72	VC_1033	1,0	2,1
Q9KPD4	glnE	1,0	1,8
Q9KL62	tdh	1,0	1,4

Q9KRR8	VC_1568	1,0	1,5
Q9KN35	VC_A0130	1,0	1,9
Q9KPM1	VC_2346	1,0	1,6
Q9KSL1	btuD	1,0	2,0
H9L4P0	VC_0237	1,0	1,4
Q9KL42	VC_A0906	1,0	1,7
Q9KPG3	murF	1,0	1,9
Q9KT41	VC_1064	1,0	2,4
Q9KUQ7	VC_0458	1,0	2,4
Q9KV39	birA	1,0	1,6
Q9KPY5	purN	1,0	1,5
P52022	dnaE	-1,0	1,3
Q9KM24	VC_A0565	-1,0	1,6
Q9KT95	VC_1008	-1,0	2,4
Q9KKY8	VC_A0962	-1,0	1,3
Q9KSE1	VC_1316	-1,0	1,4
Q9KRS9	VC_1557	-1,0	2,4
Q9KKX2	VC_A0978	-1,0	1,4
Q9KKL7	VC_A1086	-1,1	1,7
Q9KPU1	VC_2271	-1,1	1,9
Q9KUU5	VC_0420	-1,1	1,5
Q9KR91	VC_1751	-1,1	1,3
Q9KL27	VC_A0922	-1,1	2,1
Q9KNK2	hslO	-1,1	1,4
Q9KPU0	nrdR	-1,1	1,8
Q9KMP1	VC_A0283	-1,2	1,5
Q9KQK5	VC_1993	-1,2	1,7
Q9KM01	VC_A0588	-1,2	2,1
Q9KTD9	VC_0963	-1,2	1,8
Q9KPZ0	VC_2222	-1,2	2,0
Q9KLB6	VC_A0830	-1,2	1,4
Q9KP24	VC_2555	-1,2	1,5
Q9KV93	VC_0263	-1,2	1,4
Q9KTV4	VC_0783	-1,2	2,4
Q9KVW9	VC_0019	-1,2	1,9
Q9KRQ1	VC_1585	-1,3	2,6
Q9KUC2	VC_0600	-1,3	1,9
Q9KMM4	VC_A0307	-1,3	2,7
Q9KT00	dusC	-1,3	1,7
Q9KMD8	VC_A0423	-1,3	2,3
P52692	nhaR	-1,3	1,9

Q9KQQ1	VC_1947	-1,3	1,7
Q9KQH2	VC_2026	-1,4	2,0
Q9KU03	VC_0727	-1,4	2,0
Q9KPA7	VC_2466	-1,5	1,3
Q9KLV4	VC_A0637	-1,5	2,3
Q9KSQ8	VC_1198	-1,7	3,2
Q9KVJ2	VC_0152	-1,7	2,7
Q9KSV4	VC_1152	-1,7	1,3
Q9KT97	rnt	-1,7	1,9
Q9KU52	VC_0672	-1,8	1,5
Q9KTL6	VC_0886	-1,8	2,9
Q9KTA2	VC_1001	-1,8	1,9
Q9KLK2	VC_A0741	-1,9	1,7
Q9KV01	tusC	-2,0	2,1
Q9KL03	speG	-2,0	2,4
Q9KSV1	VC_1155	-2,0	1,5
Q9KT83	luxU	-2,1	3,2
Q9KSG0	VC_1296	-2,1	2,3
Q9KLR7	VC_A0675	-2,2	2,8
Q9KQ11	VC_2196	-2,4	3,3
Q9KTC9	VC_0973	-2,5	3,2
Q9KMM2	VC_A0309	-2,8	3,1
Q9KV57	VC_0300	-2,9	1,6
Q9KRD9	VC_1703	-3,3	1,4
Q9ZHV9	exbD2	-3,4	3,3
P0C6D4	flaG	-3,6	3,2
P50511	rpoH	-4,0	3,0
Q9KLS5	VC_A0666	-4,0	2,8
Q60153	tcpA	-4,0	2,7
Q9KLT8	VC_A0653	-4,0	2,6
Q9K3B3	VC_A0311	-4,0	2,5
Q9KLZ4	VC_A0597	-4,0	2,5
Q9K3D3	VC_A0328	-4,0	2,4
Q9KM15	VC_A0574	-4,0	2,3
Q9KKZ8	VC_A0952	-4,0	2,3
Q9KM47	VC_A0542	-4,0	2,3
Q9KL07	malG	-4,0	2,2
Q9KM77	VC_A0511	-4,0	2,2
Q9KL34	hmuV	-4,0	2,2
Q9KM86	VC_A0496	-4,0	2,1
Q9KLG0	fabV2	-4,0	2,1

Q9KM93	VC_A0488	-4,0	2,1
Q9KLG2	VC_A0782	-4,0	2,0
Q9KMK0	VC_A0342	-4,0	2,0
Q9KLL6	cobQ	-4,0	2,0
Q9KMP6	VC_A0274	-4,0	2,0
Q9KMX6	VC_A0190	-4,0	1,9
Q9KN34	rbsK	-4,0	1,9
Q9KN41	VC_A0124	-4,0	1,9
Q9KN83	VC_A0082	-4,0	1,8
Q9KNA8	phrA	-4,0	1,8
Q9KNG0	VC_A0004	-4,0	1,8
Q9KQT5	lapA	-4,0	1,8
Q9KNL9	VC_2713	-4,0	1,8
Q9KQX0	lpxK	-4,0	1,7
Q9KNN9	VC_2692	-4,0	1,7
Q9KR24	VC_1823	-4,0	1,7
Q9KP20	cysN	-4,0	1,7
Q9KRC5	cmoM	-4,0	1,7
Q9KPD7	cpdA	-4,0	1,7
Q9KRF5	VC_1687	-4,0	1,7
Q9KPG6	ftsW	-4,0	1,7
Q9KSJ0	VC_1266	-4,0	1,7
Q9KRG2	VC_1680	-4,0	1,7
Q9KTX9	hscB	-4,0	1,7
Q9KQ58	VC_2146	-4,0	1,6
Q9KSQ6	VC_1200	-4,0	1,6
Q9KRL7	VC_1619	-4,0	1,6
Q9KU95	VC_0628	-4,0	1,6
Q9KQJ6	VC_2002	-4,0	1,6
Q9KSX0	hisH	-4,0	1,6
Q9KRN3	VC_1603	-4,0	1,6
Q9KV94	VC_0262	-4,0	1,6
Q9KQM0	VC_1978	-4,0	1,6
Q9KT16	VC_1089	-4,0	1,6
Q9KRR9	VC_1567	-4,0	1,6
Q9KVF0	VC_0196	-4,0	1,6
Q9KQN1	VC_1967	-4,0	1,6
Q9KT20	VC_1085	-4,0	1,6
Q9KRS0	VC_1566	-4,0	1,6
Q9KVN8	VC_0103	-4,0	1,6
Q9KTD0	VC_0972	-4,0	1,6

Q9KRW8	VC_1516	-4,0	1,6
Q9KTI4	VC_0918	-4,0	1,6
Q9KRX1	VC_1512	-4,0	1,6
Q9KTJ9	syd	-4,0	1,5
Q9KRX8	VC_1505	-4,0	1,5
Q9KTM6	VC_0876	-4,0	1,5
Q9KSD8	VC_1320	-4,0	1,5
Q9KTV1	dadA	-4,0	1,5
Q9KVA1	VC_0248	-4,2	3,2
Q9KM87	VC_A0495	-4,2	3,3
Q9KKK9	VC_A1095	-4,6	4,8
Q9KQ63	fliD	-4,8	3,7
ΔdusB pm1 vs pDusB+ H2O2 (62 up, 1239 down)			
Uniprot accession	Gene name	m1/wt	neglogpadj
Q9KQF5	topB	5,8	2,3
P09545	hlyA	4,0	3,0
Q9KN34	rbsK	4,0	2,8
P50511	rpoH	4,0	2,7
Q9KRX8	VC_1505	4,0	2,7
Q9KVH6	VC_0170	4,0	2,6
Q9KNC7	VC_A0038	4,0	2,6
Q60153	tcpA	4,0	2,5
Q9KS63	VC_1397	4,0	2,5
Q9KVN8	VC_0103	4,0	2,5
Q9KNN9	VC_2692	4,0	2,5
Q9KSJ0	VC_1266	4,0	2,4
Q9K3B3	VC_A0311	4,0	2,4
Q9KNT5	argE	4,0	2,3
Q9KL07	malG	4,0	2,3
Q9KSP4	VC_1212	4,0	2,3
Q9KPG6	ftsW	4,0	2,3
Q9KSX0	hisH	4,0	2,2
Q9KL34	hmuV	4,0	2,2
Q9KPH3	VC_2395	4,0	2,2
Q9KT82	VC_1023	4,0	2,2
Q9KLG2	VC_A0782	4,0	2,2
Q9KQX0	lpxK	4,0	2,1
Q9KLL6	cobQ	4,0	2,1
Q9KTJ9	syd	4,0	2,1

Q9KRF5	VC_1687	4,0	2,1
Q9KTN7	tadA	4,0	2,0
Q9KLM0	VC_A0723	4,0	2,0
Q9KRW8	VC_1516	4,0	2,0
Q9KVB9	kdkA	4,0	2,0
Q9KLZ4	VC_A0597	4,0	2,0
Q9KRX1	VC_1512	4,0	2,0
Q9KVF0	VC_0196	4,0	2,0
Q9KTR8	VC_0822	3,7	3,7
Q9KSB0	VC_1349	3,7	4,5
Q9KVV8	VC_0030	3,7	4,5
Q9KT37	VC_1068	3,5	3,9
Q9KUM9	VC_0486	2,8	3,8
Q9KQV6	VC_1892	2,8	3,6
Q9KNS0	VC_2661	2,3	3,4
Q9KV41	VC_0317	2,2	3,2
Q9KUI9	surE	2,2	3,7
Q9KV39	birA	2,1	3,5
Q9KP97	VC_2476	2,0	2,5
Q9KST8	VC_1168	1,9	3,5
Q9KST9	tdk	1,7	3,3
Q9KP69	VC_2507	1,6	2,7
Q9KSB6	VC_1342	1,6	2,6
Q9KLY3	VC_A0608	1,5	1,6
Q9KSK9	VC_1247	1,5	1,9
Q9KN53	VC_A0112	1,4	2,2
P52695	hlyU	1,4	2,8
Q9KRY2	VC_1501	1,4	1,8
Q9KQY2	VC_1865	1,3	2,3
Q9KRG8	VC_1674	1,3	2,9
P57063	tatB	1,2	2,3
Q9KSE9	VC_1308	1,2	2,3
Q9KVE7	VC_0199	1,1	1,9
Q9KLY1	VC_A0610	1,1	2,6
Q9KSL1	btuD	1,0	2,9
Q9KQL5	VC_1983	1,0	3,0
Q9KV57	VC_0300	1,0	1,5
Q9KPX5	VC_2237	-1,0	1,8
Q9KUT4	argR	-1,0	2,1
Q9KV32	rpIJ	-1,0	2,2
Q9KT28	VC_1077	-1,0	1,7

Q9KVA5	VC_0244	-1,0	1,3
Q9KV08	hflC	-1,0	1,7
Q9KU12	rdgC	-1,0	1,5
Q9KKN1	VC_A1071	-1,0	1,9
Q9KTW7	der	-1,0	2,5
Q9KQ47	dapA	-1,0	1,8
Q9KUH4	VC_0547	-1,0	2,1
Q9KSJ2	VC_1264	-1,0	2,2
Q9KTA1	VC_1002	-1,0	1,8
Q9KUU7	VC_0418	-1,0	1,4
Q9KLH7	VC_A0767	-1,0	2,3
Q9KTF5	VC_0947	-1,0	2,4
Q9KLL1	VC_A0732	-1,0	1,5
Q9KNM0	VC_2712	-1,0	1,7
Q9KN07	VC_A0159	-1,0	1,7
Q9KQ05	VC_2202	-1,0	2,6
Q9KLT9	VC_A0652	-1,0	2,4
Q9KQY1	VC_1866	-1,0	2,5
Q9KSR8	maeA	-1,0	3,3
Q9KNR1	tpiA	-1,0	2,5
Q9KUN0	VC_0485	-1,0	3,2
Q9KVY5	mnmE	-1,0	3,1
Q9KP08	rpoA	-1,0	3,2
Q9KSY9	htpX	-1,0	2,7
Q9KV89	VC_0267	-1,0	2,0
Q9KV35	nusG	-1,0	3,3
Q06963	hldD	-1,0	3,2
Q9KQS6	clpP	-1,0	2,8
Q9KTP8	nadK	-1,0	3,2
Q9KU77	rpsO	-1,0	3,3
Q9KS85	VC_1374	-1,0	2,8
Q9KPL1	VC_2356	-1,0	2,2
Q9KQB9	VC_2081	-1,0	2,0
Q9KKP7	VC_A1055	-1,0	2,4
Q9KNG4	mnmG	-1,0	3,4
Q9KSU4	VC_1162	-1,0	2,8
Q9KQL3	VC_1985	-1,0	3,0
Q9X4Q8	nqrF	-1,0	3,2
Q9KUC8	pcnB	-1,0	2,9
Q9KTS7	VC_0811	-1,0	3,1
Q9KU86	ftsH	-1,0	3,0

Q9KLH0	VC_A0774	-1,0	3,2
Q9KQT7	pyrF	-1,0	2,4
Q9KU83	VC_0640	-1,0	2,9
Q9KTZ1	queA	-1,0	3,1
Q9KLA3	VC_A0843	-1,0	3,3
Q9KU96	erpA	-1,0	2,5
Q9KM16	VC_A0573	-1,0	2,9
Q9KV50	rho	-1,0	3,2
Q9KVX5	dnaN	-1,0	3,0
Q9KT97	rnt	-1,0	2,1
Q9KV64	prmA	-1,0	2,9
Q9KLW0	VC_A0631	-1,0	3,4
Q9KNY0	VC_2600	-1,0	2,2
Q9KQ20	ychF	-1,0	2,9
Q9KQD6	VC_2064	-1,0	2,6
Q9KQA1	VC_2099	-1,0	2,9
Q9KVM4	VC_0117	-1,0	3,0
Q9KPY2	gmhA	-1,0	3,4
Q9KLT2	VC_A0659	-1,0	2,0
Q9KNZ4	rplN	-1,0	3,3
Q9KPS3	VC_2289	-1,0	3,1
Q9KQI4	VC_2014	-1,0	2,8
Q9KPG5	murD	-1,0	2,1
Q9KVS3	VC_0066	-1,0	3,1
Q9KSK0	VC_1256	-1,0	3,4
Q9KNC2	VC_A0043	-1,0	2,6
Q9KV30	rpoB	-1,0	3,4
Q9KT24	VC_1081	-1,0	2,5
Q9KU16	VC_0714	-1,0	1,6
Q84I33	ftsK	-1,0	3,3
Q9KS19	VC_1442	-1,0	3,2
Q9KSJ8	gyrA	-1,0	3,2
Q9KUF8	trmD	-1,0	3,3
Q9KU31	VC_0698	-1,0	3,1
P52116	smpB	-1,0	3,1
Q9KKM1	VC_A1082	-1,0	2,7
Q9KNS8	secB	-1,0	3,1
Q9KTD3	cysZ	-1,0	2,3
Q9KSY0	VC_1126	-1,0	3,2
Q9KPU1	VC_2271	-1,0	3,2
Q9KUF1	rplM	-1,0	3,2

Q9KSN6	pheT	-1,0	3,3
Q9KLU7	VC_A0644	-1,0	3,0
Q9KPH9	carB	-1,0	3,3
Q9KU63	srmB	-1,0	3,4
Q9KR62	nanE	-1,0	2,0
Q9KPU3	ribB	-1,0	1,9
Q9KT56	VC_1049	-1,0	2,5
Q9KUC5	sfsA	-1,0	3,2
Q9KLE4	VC_A0802	-1,0	3,0
Q9KSW5	VC_1141	-1,0	2,8
Q9KQS7	clpX	-1,0	3,3
P78283	secY	-1,0	3,4
Q9KNF0	VC_A0014	-1,0	3,1
Q9KTC9	VC_0973	-1,0	2,4
Q9KRJ1	VC_1649	-1,0	2,7
Q9KQ75	VC_2126	-1,0	2,7
Q9KUW4	VC_0395	-1,0	3,3
Q9KP90	VC_2483	-1,0	3,4
Q9KLX1	VC_A0620	-1,0	3,3
Q9KVK6	cdgJ	-1,0	3,2
Q9KL54	VC_A0894	-1,0	1,8
Q9KV04	VC_0354	-1,0	3,1
Q9KQH4	plsX	-1,0	1,9
Q9KMZ8	VC_A0168	-1,0	2,5
Q9KM60	VC_A0528	-1,0	3,3
Q9KRC4	VC_1718	-1,0	2,3
Q9KNU5	mrcA	-1,0	3,0
Q9KNS7	VC_2654	-1,0	3,3
Q9KSW9	hisA	-1,0	2,6
Q9KN27	VC_A0139	-1,0	3,3
Q9KLT5	VC_A0656	-1,0	3,1
Q9KR12	pal	-1,1	3,1
Q9KR76	VC_1767	-1,1	3,2
Q9KSU0	aspS	-1,1	3,1
Q9KNY6	rplW	-1,1	3,4
Q9KNY7	rplB	-1,1	3,4
Q9KQJ1	VC_2007	-1,1	1,7
Q9KQU3	VC_1905	-1,1	3,4
Q9KL41	hutZ	-1,1	3,1
Q9KL65	VC_A0882	-1,1	2,4
Q9KM23	VC_A0566	-1,1	2,8

Q9KRL4	nspC	-1,1	3,1
P0C6C3	flaA	-1,1	3,3
Q9KQI5	VC_2013	-1,1	2,8
Q9KL52	zwf	-1,1	3,3
Q9KTW3	guaB	-1,1	3,4
Q9KR07	VC_1840	-1,1	3,1
O34241	dnaK	-1,1	3,3
Q9KQH7	fabG	-1,1	3,1
Q9KNZ6	rplE	-1,1	3,3
Q9KP59	VC_2517	-1,1	2,5
Q9KKS1	VC_A1029	-1,1	2,5
Q9KSR1	VC_1195	-1,1	3,4
Q9KQ85	aroC	-1,1	3,2
Q9KKL8	VC_A1085	-1,1	1,8
Q9KPU2	VC_2270	-1,1	2,9
Q9KUY6	alr1	-1,1	3,3
Q9KS22	VC_1439	-1,1	3,3
Q9KKL5	VC_A1088	-1,1	2,3
Q9KPM5	fusA2	-1,1	3,0
Q9KTZ9	VC_0731	-1,1	3,2
H9L4S9	VC_0415	-1,1	3,2
Q9KN89	VC_A0076	-1,1	2,8
Q9KT07	ackA1	-1,1	2,8
Q9KTY6	secF	-1,1	2,8
Q9KVQ2	VC_0089	-1,1	2,8
Q9KNT8	argG	-1,1	3,1
Q9KMY7	VC_A0179	-1,1	3,2
Q9KQA3	VC_2097	-1,1	2,9
Q9KV03	VC_0355	-1,1	2,1
Q9KPY7	upp	-1,1	3,1
Q9KUW5	uvrA	-1,1	3,3
Q9KVA2	VC_0247	-1,1	3,3
Q9KPV3	tsf	-1,1	3,3
Q9KVL7	lysA	-1,1	3,0
Q9KQZ9	cysS	-1,1	3,1
Q9KSV1	VC_1155	-1,1	3,3
Q9KUD1	panC	-1,1	3,1
Q9KVX6	dnaA	-1,1	3,4
Q9KMZ6	VC_A0170	-1,1	1,6
Q9KVS7	thiE	-1,1	3,4
Q9KUA1	VC_0622	-1,1	2,8

Q9KUJ6	VC_0524	-1,1	2,9
Q9KNR6	groS1	-1,1	3,3
Q9KPE0	parC	-1,1	3,2
Q9KQJ0	VC_2008	-1,1	3,1
Q9KP00	rplR	-1,1	3,3
Q9KNI7	add	-1,1	2,2
Q9KM72	VC_A0516	-1,1	3,1
P0C6Q3	pgk	-1,1	2,9
Q9KS57	VC_1403	-1,1	2,6
Q9KT74	VC_1031	-1,1	3,4
Q9KU74	VC_0649	-1,1	3,2
Q9KTJ4	gmhB	-1,1	1,9
Q9KUG0	rpsP	-1,1	3,3
Q9KP43	VC_2536	-1,1	3,1
Q9KV25	VC_0333	-1,1	2,5
Q9KTF1	rlmH	-1,1	3,1
Q9KPW2	lpxD	-1,1	3,4
Q9KR78	VC_1765	-1,1	3,1
Q9KTK1	VC_0901	-1,1	2,3
Q9KQP8	VC_1950	-1,1	3,3
Q9KKR0	VC_A1042	-1,1	3,4
Q9KUY7	VC_0371	-1,1	3,4
Q9KU84	glmM	-1,1	3,3
Q9KV46	VC_0312	-1,1	2,0
Q9KPN3	VC_2333	-1,1	2,0
Q9KL05	VC_A0945	-1,1	2,8
Q9KUG1	ffh	-1,1	3,1
Q9KQ52	dapE	-1,1	2,6
Q9KPC1	rlmD	-1,1	2,8
Q9KQB4	VC_2086	-1,1	3,1
Q9KMU2	VC_A0227	-1,1	3,0
Q9KSK4	VC_1252	-1,1	3,4
Q9K2Y1	tolC	-1,1	3,1
Q9KTS4	VC_0814	-1,1	1,9
O68844	infC	-1,1	3,2
Q9KPK1	VC_2366	-1,1	3,2
Q9KT51	dnaX	-1,1	3,3
Q9KVF8	VC_0188	-1,1	2,8
Q9KVB0	VC_0236	-1,1	2,4
Q9KUR6	VC_0449	-1,1	2,5
Q9KVW8	glyS	-1,1	3,4

Q9KTG1	glyA1	-1,1	2,8
Q9KRW4	VC_1520	-1,1	2,8
Q9KM70	VC_A0518	-1,1	3,4
Q9KNH2	atpH	-1,1	3,1
Q9KUG4	luxS	-1,1	3,2
Q9KNP5	zapB	-1,1	3,3
Q9KR97	VC_1745	-1,1	3,0
Q9KQA5	VC_2095	-1,1	3,1
P45383	recA	-1,1	3,3
Q9KNW6	VC_2614	-1,1	3,3
Q9KVN0	engB	-1,1	3,1
Q9KNV2	aroB	-1,1	3,3
Q9KQ83	prmB	-1,1	2,3
Q9KNN7	VC_2694	-1,1	3,2
P23247	asd2	-1,1	3,4
Q9KVC4	coaD	-1,1	2,8
Q9KPF3	VC_2415	-1,1	3,4
Q9KRS8	VC_1558	-1,1	3,1
Q9KU64	prfC	-1,1	2,4
Q9KQB3	VC_2087	-1,1	3,2
Q9KM57	VC_A0531	-1,1	3,3
Q9KNH0	atpE	-1,1	3,2
Q9KU07	ppk	-1,1	2,0
H9L4T2	VC_0401	-1,1	3,2
Q9KRG5	VC_1677	-1,1	2,6
Q9KTD2	zipA	-1,1	3,2
Q9KP76	VC_2499	-1,1	3,4
Q9KQ06	cheR1	-1,1	3,4
Q9KT63	VC_1042	-1,1	2,5
P0C6D4	flaG	-1,1	2,1
H9L4R0	VC_0260	-1,1	3,4
Q9KL61	kbl	-1,1	3,4
Q9KUF5	VC_0566	-1,1	3,3
Q9KVY1	rpmH	-1,1	2,9
Q9KTT1	VC_0807	-1,1	3,1
Q9KP44	VC_2535	-1,1	3,0
Q9KUN5	VC_0480	-1,1	3,2
Q9KU75	VC_0648	-1,1	2,7
Q9KT90	rnfG	-1,1	3,1
Q9KPT2	VC_2280	-1,1	2,7
Q9KTG0	VC_0942	-1,1	2,5

Q9KM35	VC_A0554	-1,1	2,8
Q9KNQ0	VC_2681	-1,1	3,5
Q9KL70	VC_A0877	-1,1	2,9
Q9KV26	hemE	-1,1	2,6
Q9KSE4	VC_1313	-1,1	3,0
Q9KKZ9	VC_A0951	-1,1	3,1
O30862	grpE	-1,1	3,4
Q9KMG2	VC_A0396	-1,1	3,2
Q9KSA0	VC_1359	-1,1	2,2
Q9KUR9	lptD	-1,1	3,2
Q56648	alaS	-1,1	3,5
Q9KMT5	ackA2	-1,1	3,2
Q9KSU3	cmoB	-1,1	3,4
Q9KNY1	rnr	-1,1	3,4
Q9KP91	VC_2482	-1,1	3,2
Q9KVJ2	VC_0152	-1,1	3,3
Q9KN77	VC_A0088	-1,1	3,2
Q9KUU1	rraB	-1,1	3,3
Q9KNJ4	VC_2744	-1,1	3,4
Q9KR86	VC_1756	-1,1	3,3
Q9KSM2	VC_1234	-1,1	3,1
Q9KL51	pgl	-1,1	3,2
Q9KMN7	thrS	-1,1	3,5
Q9KMW8	VC_A0198	-1,1	1,8
Q9KV09	hflK	-1,1	3,2
Q9KQD2	VC_2068	-1,1	3,3
Q9KS77	VC_1382	-1,1	3,4
Q9KVS8	thiC	-1,1	3,5
Q9KRH9	VC_1663	-1,1	3,4
Q9KVC2	VC_0224	-1,1	3,0
Q9KVW7	glyQ	-1,1	3,2
Q9KMT9	VC_A0230	-1,2	2,3
Q9KUQ5	proC	-1,2	2,9
Q9KQZ3	VC_1854	-1,2	3,4
Q9KR03	VC_1844	-1,2	3,4
P0C6E1	pepA	-1,2	3,0
P0C6C6	flaD	-1,2	3,4
Q9KTB0	VC_0993	-1,2	3,3
Q9KUS0	surA	-1,2	3,4
Q9KVX0	VC_0018	-1,2	2,9
Q9KTF9	lipA	-1,2	3,3

Q9KP35	fbp	-1,2	3,4
Q9KRA3	fabV	-1,2	3,1
Q9KNM9	nhaP2	-1,2	3,0
Q9KSF2	VC_1304	-1,2	3,4
Q9KUD7	VC_0585	-1,2	2,8
Q9KNQ7	hslU	-1,2	3,2
Q9KQE7	ccmE	-1,2	2,9
Q9KVA0	VC_0250	-1,2	3,4
Q9KNZ9	rplF	-1,2	3,4
Q9KPJ8	VC_2369	-1,2	2,7
Q9KSF5	VC_1301	-1,2	3,1
Q9KN63	VC_A0102	-1,2	3,3
Q9KV74	VC_0282	-1,2	2,5
Q9KNF4	VC_A0010	-1,2	2,8
Q9KNW2	argD	-1,2	2,6
Q9KQG2	asd1	-1,2	3,3
Q9KM17	ddl	-1,2	3,0
Q9KUW2	ssb	-1,2	3,4
P22359	htpG	-1,2	3,2
Q9KQV3	VC_1895	-1,2	2,9
Q9KTQ6	VC_0841	-1,2	3,0
Q9KT14	VC_1091	-1,2	3,2
Q9KKS9	VC_A1021	-1,2	3,3
Q9KR14	nadA	-1,2	2,7
Q9KS02	matP	-1,2	2,9
Q9KUK0	VC_0519	-1,2	2,3
Q9KSQ8	VC_1198	-1,2	3,4
Q9KNN3	VC_2698	-1,2	3,4
Q9KLA6	VC_A0840	-1,2	3,3
Q9KS54	VC_1406	-1,2	3,2
Q9KVH1	VC_0175	-1,2	3,3
H9L4R2	VC_1548	-1,2	2,5
Q9KTC5	VC_0977	-1,2	3,3
Q9KRC8	mukB	-1,2	3,2
Q9KNH4	atpG	-1,2	3,4
Q9KSC4	VC_1334	-1,2	2,8
Q9KS46	VC_1414	-1,2	3,4
Q9KPC2	VC_2450	-1,2	3,4
Q9KTK0	queF	-1,2	2,3
Q9KPY6	purM	-1,2	2,6
Q9KL53	VC_A0895	-1,2	2,4

Q9KPW0	bamA	-1,2	3,4
Q9KR08	tolQ	-1,2	2,7
Q9KSQ4	hutH	-1,2	2,6
Q9KU57	VC_0667	-1,2	3,2
Q9KLZ7	VC_A0592	-1,2	3,3
Q9KR88	VC_1754	-1,2	3,2
Q9KTJ1	VC_0911	-1,2	2,8
Q9KQB5	sucC	-1,2	3,4
Q9KU33	VC_0696	-1,2	2,9
Q9KNX8	purA	-1,2	3,2
Q9KNN2	VC_2699	-1,2	3,3
Q9KP30	msrA	-1,2	1,7
Q9KQ89	VC_2112	-1,2	2,3
Q9KRZ2	pyrD	-1,2	3,2
Q9KTD4	VC_0968	-1,2	3,3
Q9KLK9	VC_A0734	-1,2	2,6
Q9KVD0	VC_0216	-1,2	3,5
Q9KQ90	VC_2111	-1,2	2,6
Q9KT80	VC_1025	-1,2	2,5
Q9KUM8	glmS	-1,2	3,4
Q9KLK2	VC_A0741	-1,2	2,1
Q9KMW6	VC_A0200	-1,2	2,7
Q9KU89	greA	-1,2	3,3
Q9KTY1	VC_0749	-1,2	3,3
Q9KNQ5	VC_2676	-1,2	3,4
Q9KT26	VC_1079	-1,2	2,7
Q9KVM3	VC_0118	-1,2	3,1
Q9KLP4	glgC2	-1,2	2,2
Q9KU91	VC_0632	-1,2	2,8
Q9KNP2	pfkA	-1,2	3,4
Q9KP46	npr	-1,2	2,5
Q9KVX3	gyrB	-1,2	3,4
Q9KMN1	VC_A0300	-1,2	3,5
Q9KR11	tolB	-1,2	3,2
Q9KNK0	pckA	-1,2	3,2
Q9KRK2	VC_1638	-1,2	3,2
Q9KPJ9	VC_2368	-1,2	3,5
Q9KQ14	flgI	-1,2	3,3
Q9KKR7	VC_A1034	-1,2	3,1
Q9KP80	leuD	-1,2	2,9
Q9KSE7	VC_1310	-1,2	3,2

Q9KUP3	metK	-1,2	3,2
Q9KTN5	mltF	-1,2	2,3
Q9KTA3	accD	-1,2	3,2
Q9KQ57	VC_2147	-1,2	3,3
Q9KU47	ileS	-1,2	3,4
Q9KVR1	VC_0078	-1,2	3,4
Q9KP89	VC_2484	-1,2	3,4
Q9KPX9	dnaQ	-1,2	3,3
Q9KQU4	VC_1904	-1,2	3,1
Q9KSZ6	serS	-1,2	3,2
Q9KN50	VC_A0115	-1,2	2,4
Q9K344	VC_0256	-1,2	3,2
Q9KS33	potA	-1,2	3,4
Q9KNZ2	rpmC	-1,2	3,1
Q9KQ48	bamC	-1,2	3,2
Q9KU44	ispH	-1,2	3,5
Q9KNL8	VC_2714	-1,2	2,1
Q9KP58	VC_2518	-1,2	2,9
Q9KQ92	pdxB	-1,2	3,4
Q9KNU3	VC_2637	-1,2	3,1
Q9KTX7	VC_0753	-1,2	2,8
Q9KNJ8	VC_2740	-1,2	2,7
Q9KNN6	trmL	-1,2	3,2
Q9KKQ5	VC_A1047	-1,2	2,4
Q9KVP2	VC_0099	-1,2	2,9
Q9KTB7	adk	-1,2	2,8
Q9KKJ3	VC_A1114	-1,2	3,0
Q9KSU2	cmoA	-1,2	3,4
Q9KMA0	VC_A0478	-1,2	2,9
Q9KMW7	VC_A0199	-1,2	3,2
Q9KQS8	lon	-1,2	3,1
Q9KQD9	VC_2061	-1,2	2,6
Q9KS20	VC_1441	-1,2	3,3
Q9KSX8	mnmA	-1,2	3,4
Q9KVD7	VC_0209	-1,2	3,5
Q9KLK3	VC_A0740	-1,2	3,3
Q9KU92	tyrS2	-1,2	3,5
Q9KUT5	VC_0430	-1,2	3,4
Q9KNP3	VC_2688	-1,2	3,3
Q9KUD6	VC_0586	-1,2	3,4
Q9KST7	trpA	-1,2	3,0

Q9KTV6	VC_0781	-1,2	3,4
Q9KU70	VC_0653	-1,2	3,3
Q9KTD6	ptsH	-1,2	3,4
Q9KQU9	VC_1899	-1,2	2,7
Q9KPH1	ftsZ	-1,2	3,5
Q9KN62	VC_A0103	-1,2	3,0
Q9KV62	VC_0295	-1,2	3,4
Q9KQD7	VC_2063	-1,2	3,3
Q9KNV7	trpS	-1,2	3,3
Q9KUG7	VC_0554	-1,2	3,3
Q9KM75	VC_A0513	-1,2	3,3
Q9KTQ7	VC_0840	-1,2	2,2
Q9KVD8	VC_0208	-1,2	2,6
Q9KRD3	VC_1709	-1,2	3,5
Q9KLI1	VC_A0763	-1,2	3,2
Q9KVY4	yidC	-1,2	3,2
Q9KSU8	VC_1158	-1,2	3,2
Q9KRB6	glgA	-1,2	2,8
Q9KQ30	nutA	-1,2	3,3
Q9KUB8	VC_0604	-1,2	3,4
Q9KNB0	VC_A0055	-1,2	3,0
P57066	lolD	-1,2	3,2
Q9KLG5	VC_A0779	-1,2	2,3
Q9KTE0	miaB	-1,2	3,3
Q9KP40	VC_2539	-1,2	3,2
Q9KSN7	pheS	-1,3	3,5
Q9KTK4	xni	-1,3	3,5
Q9KSX6	VC_1130	-1,3	3,5
Q9KQC6	argS	-1,3	3,4
Q9KMY2	VC_A0184	-1,3	3,2
Q9KQB6	sucD	-1,3	3,5
P66367	rpsK	-1,3	3,4
Q9KVM1	hemC	-1,3	3,3
Q9KP70	rapA	-1,3	3,1
Q9KL50	VC_A0898	-1,3	3,4
Q9KQG1	VC_2037	-1,3	3,3
Q9KNK2	hslO	-1,3	3,5
Q9KPM0	deoD1	-1,3	3,3
Q9KU80	infB	-1,3	3,5
Q9KKZ4	vdcA	-1,3	1,5
Q9KPN7	dapD	-1,3	3,3

Q9KVM7	yihI	-1,3	3,4
Q9KUQ7	VC_0458	-1,3	3,1
Q9KSF8	VC_1298	-1,3	3,0
Q9KNH6	atpC	-1,3	2,9
Q9KNX7	VC_2603	-1,3	3,5
Q9KUQ6	VC_0459	-1,3	3,3
Q9KTM1	VC_0881	-1,3	2,9
Q9KNS1	efp	-1,3	3,3
Q9KVH8	VC_0168	-1,3	2,8
Q9KUX7	VC_0381	-1,3	3,3
Q9KQX8	VC_1869	-1,3	2,9
Q9KNT0	gpsA	-1,3	3,5
Q9KTA8	VC_0995	-1,3	3,5
Q9KNF8	VC_A0006	-1,3	3,3
Q9KS84	VC_1375	-1,3	2,9
Q9KVD1	coaBC	-1,3	3,3
Q9KNV6	gph	-1,3	2,8
Q9KMM3	VC_A0308	-1,3	3,4
Q9KV48	ubiD	-1,3	3,5
Q9KL42	VC_A0906	-1,3	2,7
P57051	tatA	-1,3	2,8
Q9KQY7	VC_1860	-1,3	3,3
Q9KP62	murA	-1,3	3,0
Q9KLK6	luxP	-1,3	3,5
Q9KPF5	VC_2413	-1,3	3,5
Q9KS24	VC_1437	-1,3	3,2
Q9KNQ6	hslV	-1,3	3,4
Q9KPM4	radA	-1,3	3,2
H9L4R8	VC_1447	-1,3	2,5
Q9KRZ1	VC_1492	-1,3	3,4
Q9KM74	VC_A0514	-1,3	3,1
Q9KUM3	VC_0492	-1,3	3,4
Q9KUE5	VC_0576	-1,3	3,4
Q9KUE7	VC_0574	-1,3	3,2
Q9KP31	VC_2548	-1,3	2,6
Q9KPD9	parE	-1,3	3,5
Q9KTK3	VC_0899	-1,3	2,4
Q9KUT3	mdh	-1,3	2,9
Q9KQ40	VC_2164	-1,3	3,4
Q9KVJ6	ftsY	-1,3	2,8
Q9KUK2	VC_0516	-1,3	2,5

Q9KTD8	VC_0964	-1,3	3,4
Q9KQX2	kdsB	-1,3	3,1
Q9KNR9	VC_2662	-1,3	2,2
Q9KVB5	VC_0231	-1,3	3,4
Q9KR87	VC_1755	-1,3	2,3
Q9KU18	clpB	-1,3	3,4
P0C6D3	vibB	-1,3	3,5
Q9KRY7	VC_1496	-1,3	3,3
Q9KVX4	recF	-1,3	1,9
Q9KPV5	frr	-1,3	3,0
Q9KNL2	nfuA	-1,3	2,6
Q9KTK2	VC_0900	-1,3	3,3
Q9KRY0	VC_1503	-1,3	3,3
Q9KPY9	VC_2223	-1,3	3,3
Q9KPT4	VC_2278	-1,3	3,4
Q9KRN4	VC_1602	-1,3	2,3
Q9KUN7	VC_0478	-1,3	2,9
Q9KVI4	ilvC	-1,3	3,4
Q9KP93	rpiA	-1,3	3,4
Q9KLL7	VC_A0726	-1,3	2,6
Q9KSJ9	ubiG	-1,3	3,2
Q9KQN3	VC_1965	-1,3	3,4
Q9KSZ4	bioB	-1,3	3,5
Q9KTJ8	VC_0904	-1,3	3,3
Q9KV28	VC_0330	-1,3	3,4
Q9KT46	VC_1059	-1,3	3,4
Q9KTL3	dxs	-1,3	3,4
P15795	toxR	-1,3	2,9
O34242	dnaJ	-1,3	3,0
Q9KN88	VC_A0077	-1,3	2,6
Q06951	rfbB	-1,3	3,5
Q9KQR9	dctP	-1,3	3,0
Q9KTA6	glnS	-1,3	3,5
Q9KNX2	VC_2608	-1,3	3,4
P0C6C4	flaB	-1,3	3,0
Q9KVD2	slmA	-1,3	3,2
Q9KQ29	kdsA	-1,3	3,4
Q9KUY4	pgi	-1,3	3,5
Q9KP17	VC_2562	-1,3	3,3
Q9KMW1	nadE	-1,3	3,0
Q9KVK8	VC_0134	-1,3	2,2

Q9KSB4	VC_1345	-1,3	2,1
Q9KQH0	rluC	-1,3	3,5
Q9KUD3	VC_0589	-1,3	2,4
Q9KT69	metG	-1,3	3,5
Q9KLJ2	VC_A0752	-1,3	2,6
Q9KPE6	VC_2422	-1,3	3,2
Q9KPT9	proA	-1,3	3,6
Q9KSJ7	VC_1259	-1,3	3,4
H9L4Q7	VC_2528	-1,3	3,4
Q9KUC0	mrcB	-1,3	3,2
Q9KVG7	dncV	-1,3	3,4
Q9KQT9	VC_1909	-1,3	3,4
Q9X4Q6	nqrD	-1,3	3,5
Q9KV53	gppA	-1,3	3,5
Q9KRL5	VC_1622	-1,3	3,3
Q9KUM5	VC_0490	-1,3	3,4
Q9KM21	VC_A0568	-1,3	3,2
Q9KP34	ppa	-1,4	3,5
Q9KVL4	xerC	-1,4	2,1
Q9KUW8	VC_0391	-1,4	3,0
Q9KPL7	deoC	-1,4	3,0
Q9KPH7	dapB	-1,4	3,2
Q9KQT8	VC_1910	-1,4	3,5
Q9KP78	VC_2497	-1,4	2,6
Q9KS18	VC_1443	-1,4	3,2
Q9KTY0	VC_0750	-1,4	3,4
Q9KT71	VC_1034	-1,4	3,5
Q9KR67	VC_1776	-1,4	2,2
Q9KKR1	VC_A1041	-1,4	2,9
Q9KVK4	VC_0139	-1,4	3,5
Q9KSW4	cspD	-1,4	2,9
Q9KVI8	VC_0157	-1,4	2,7
Q9KV79	VC_0277	-1,4	3,5
Q9KT68	VC_1037	-1,4	3,5
Q9KVJ0	trmA	-1,4	3,4
Q9KMA6	higB-2	-1,4	3,2
Q9KUR2	trmB	-1,4	3,4
Q9KQH6	VC_2022	-1,4	3,5
Q9KP73	valS	-1,4	3,5
Q9KU27	VC_0702	-1,4	3,3
Q9KRH1	VC_1671	-1,4	2,9

Q9KRY9	VC_1494	-1,4	3,4
Q9KPS2	nqrB	-1,4	3,2
Q9KTZ4	VC_0736	-1,4	3,4
Q9KUK1	rpoD	-1,4	3,3
Q9KM27	VC_A0562	-1,4	3,5
Q9KUS2	rsmA	-1,4	3,5
Q9KPT3	VC_2279	-1,4	3,5
Q9KLF1	VC_A0795	-1,4	3,2
Q9KPQ7	csd	-1,4	2,8
Q9KKP2	ribB	-1,4	2,5
Q9KS97	VC_1362	-1,4	3,1
Q9KKV3	VC_A0997	-1,4	3,2
Q9KV60	aroQ	-1,4	3,4
Q9KR84	VC_1758	-1,4	2,3
Q9KLQ2	VC_A0690	-1,4	3,1
Q9KV93	VC_0263	-1,4	3,2
Q9KMZ0	VC_A0176	-1,4	3,4
Q9KVN6	VC_0105	-1,4	3,4
Q9KUK4	VC_0514	-1,4	3,1
Q9KSW1	VC_1145	-1,4	3,5
Q9KUI3	VC_0538	-1,4	3,2
Q9KRN0	VC_1606	-1,4	2,2
Q9KPU8	VC_2264	-1,4	2,9
Q9KUP2	tkt1	-1,4	3,5
Q9KS95	VC_1364	-1,4	2,0
Q9KT67	udk	-1,4	3,6
Q9KRC6	mukF	-1,4	3,5
Q9KS35	VC_1426	-1,4	3,5
Q9KM67	sbcC	-1,4	3,2
Q9KUZ7	fusA1	-1,4	3,4
Q9KQ25	prfA	-1,4	3,4
Q9KSG0	VC_1296	-1,4	3,2
Q9KLD5	gbpA	-1,4	3,5
Q9KNV1	aroK	-1,4	3,4
Q9KV17	orn	-1,4	2,9
Q9KSP7	VC_1209	-1,4	3,1
Q9KRP5	VC_1591	-1,4	2,7
Q9KVU5	rsmB	-1,4	3,1
Q9KUB5	VC_0608	-1,4	3,5
Q9KSW0	grx	-1,4	2,4
Q9KTX0	hisS	-1,4	3,5

Q9KU99	VC_0624	-1,4	3,2
Q9KSS7	rluB	-1,4	3,5
Q9KTE6	leuS	-1,4	3,6
Q9KL13	VC_A0936	-1,4	3,0
Q9KSP2	uvrC	-1,4	3,3
Q9KPK5	VC_2362	-1,4	3,3
Q9KLS9	VC_A0662	-1,4	3,3
Q9KVD4	lpxM	-1,4	2,6
Q9KQL1	VC_1987	-1,4	2,8
Q9KL03	speG	-1,4	2,6
Q9KRS6	katG	-1,4	3,5
Q9KM50	VC_A0539	-1,4	3,6
Q9KS36	VC_1425	-1,4	2,9
Q9KUH3	csrA	-1,4	3,5
Q9KNT2	VC_2649	-1,4	3,5
Q9KSF9	asnS	-1,4	3,5
Q9KTF2	mrdA	-1,4	2,9
P32557	dsbA	-1,4	3,3
Q9KPF0	VC_2418	-1,4	3,4
Q9KQM5	menB	-1,4	3,2
Q9KU79	rbfA	-1,4	3,4
Q9KVH2	VC_0174	-1,4	2,8
Q9KSL0	VC_1246	-1,4	3,1
Q9KR91	VC_1751	-1,4	2,4
Q9KU60	lysS	-1,4	3,5
Q9KVQ4	ubiB	-1,4	3,3
Q9KSQ9	VC_1197	-1,4	3,2
Q9KTD7	VC_0965	-1,4	3,6
Q9KU59	VC_0665	-1,4	3,4
Q9KU21	bamD	-1,4	3,3
Q9KP88	VC_2485	-1,4	2,8
Q9KM95	VC_A0486	-1,4	3,4
Q9KQE0	VC_2060	-1,4	2,9
Q9KRT2	VC_1554	-1,4	3,5
Q9KSP1	VC_1215	-1,4	3,4
Q9KQA8	VC_2092	-1,4	3,6
Q9KT50	VC_1055	-1,4	3,1
Q9KVR6	rsmJ	-1,4	2,9
Q9KM56	VC_A0532	-1,4	2,9
Q9KNC5	VC_A0040	-1,4	3,0
Q9KVI2	VC_0164	-1,4	3,0

Q9KPR4	VC_2302	-1,4	3,4
Q9KNY2	rlmB	-1,4	3,6
Q9KSE2	VC_1315	-1,4	3,1
Q9KP02	rpmD	-1,4	3,2
Q9KPF2	VC_2416	-1,4	3,4
Q9KUS3	apaG	-1,4	3,5
Q9KUG9	VC_0552	-1,4	3,4
Q9KVY6	mioC	-1,4	2,8
Q9KPC4	pyrG	-1,4	3,5
Q9KTX8	hscA	-1,4	3,4
Q9KQD8	cheB1	-1,4	3,5
Q9KQ24	hemA	-1,5	3,0
P29485	tcpP	-1,5	3,4
Q9KNB7	VC_A0048	-1,5	2,8
Q9KRU5	VC_1539	-1,5	3,2
Q9KUW7	VC_0392	-1,5	3,4
Q9KUD9	rsmI	-1,5	3,0
Q9KMZ2	VC_A0174	-1,5	3,2
Q9KNC8	VC_A0037	-1,5	3,3
Q9KQ59	VC_2145	-1,5	3,6
Q9KUJ7	plsY	-1,5	3,2
Q9KSB3	VC_1346	-1,5	2,4
Q9KST5	trpCF	-1,5	2,0
P57069	lolA	-1,5	3,6
P0C6Q1	gltX	-1,5	3,6
Q9KLX9	mscL	-1,5	3,4
Q9KMZ9	VC_A0167	-1,5	3,3
Q9KSS2	VC_1184	-1,5	3,1
Q9KTT2	VC_0806	-1,5	3,5
Q9KUR4	VC_0451	-1,5	2,8
Q9KPS7	VC_2285	-1,5	2,7
Q9KUG5	gshA	-1,5	3,3
Q9KVB2	VC_0234	-1,5	2,9
Q9KTX2	VC_0758	-1,5	3,1
Q9KLJ9	glpK	-1,5	3,6
Q9KPK3	VC_2364	-1,5	3,5
Q9KLB0	VC_A0836	-1,5	3,2
Q9KVS6	VC_0063	-1,5	3,2
Q9KU41	VC_0688	-1,5	3,3
Q9KV87	VC_0269	-1,5	3,4
Q9KTX1	ispG	-1,5	3,6

Q9KQP2	VC_1956	-1,5	3,5
Q9KR13	cpoB	-1,5	3,3
Q9KNW0	rimO	-1,5	3,6
Q9X4Q9	fliG	-1,5	3,5
Q9KL66	VC_A0881	-1,5	3,0
Q9KQH2	VC_2026	-1,5	2,6
Q9KUJ8	VC_0522	-1,5	3,0
Q9KPJ0	VC_2377	-1,5	2,8
Q9KN26	VC_A0140	-1,5	2,4
Q9KUU0	VC_0425	-1,5	2,4
Q9KS21	VC_1440	-1,5	3,0
Q9KKP3	VC_A1059	-1,5	3,4
Q9KQH5	fabH1	-1,5	3,5
Q9KL26	VC_A0923	-1,5	3,1
Q9KPN4	VC_2332	-1,5	3,5
Q9KUD5	VC_0587	-1,5	3,3
Q9KT59	fadI	-1,5	2,8
Q9KQF8	VC_2040	-1,5	2,7
Q9KUE8	VC_0573	-1,5	3,2
Q9KSG8	opgG	-1,5	3,5
Q9KQD4	fliA	-1,5	3,4
Q9KLF8	VC_A0786	-1,5	3,2
Q9KSF6	VC_1300	-1,5	3,6
Q9KTD9	VC_0963	-1,5	3,3
Q9KPG8	murC	-1,5	3,5
Q9KPI8	mtnN	-1,5	3,3
Q9KKY8	VC_A0962	-1,5	2,6
Q9KPX2	padC	-1,5	3,6
Q9KT79	moaC	-1,5	3,3
Q9KUR3	VC_0452	-1,5	3,0
Q9KLW7	tkt2	-1,5	3,4
Q9KMQ5	VC_A0265	-1,5	3,2
Q9KP03	rplO	-1,5	3,4
Q9KKL9	VC_A1084	-1,5	3,4
Q9KTE4	lnt	-1,5	2,9
Q9KVS4	thiG	-1,5	3,5
Q9KNS6	epmA	-1,5	3,3
P0C6Q5	tcpF	-1,5	3,5
Q9KTF7	VC_0945	-1,5	3,5
Q9KP29	VC_2550	-1,5	2,7
Q9KKQ9	VC_A1043	-1,5	3,5

Q9KU13	VC_0717	-1,5	3,6
Q9KUF6	VC_0565	-1,5	3,0
Q9KNA2	VC_A0063	-1,5	3,4
O34419	rstR1	-1,5	3,7
Q9KU00	cutC	-1,5	2,8
Q9KTX4	ndk	-1,5	3,4
Q9KRM9	VC_1607	-1,5	3,4
Q9KQV0	VC_1898	-1,5	3,4
Q9KU42	VC_0687	-1,5	3,0
Q9KP64	VC_2512	-1,5	2,7
Q9KPK4	thrB	-1,5	3,0
Q9KR01	ruvA	-1,5	3,1
Q9KTZ3	VC_0737	-1,5	3,2
Q9KNK3	VC_2735	-1,5	3,2
Q9KLX3	VC_A0618	-1,6	3,6
Q9KSG9	opgH	-1,6	3,2
Q9KQT2	cmk	-1,6	3,1
Q9KL24	pyrC	-1,6	3,4
Q9KQF4	VC_2044	-1,6	3,2
Q9KMW3	VC_A0205	-1,6	3,3
Q9KUN2	VC_0483	-1,6	3,5
Q9KVV3	VC_0036	-1,6	3,3
Q9KTS3	VC_0815	-1,6	3,3
Q9KP61	VC_2515	-1,6	3,4
Q9KPW1	skp	-1,6	3,5
Q9KPA4	nadB	-1,6	2,5
Q9K3D4	VC_A0349	-1,6	3,3
Q9KSJ3	ribA	-1,6	3,5
Q9KQZ8	VC_1849	-1,6	3,6
Q9KRI3	VC_1658	-1,6	3,3
Q9KU15	VC_0715	-1,6	3,0
Q9KS50	VC_1410	-1,6	3,3
Q9KPF9	rsmH	-1,6	3,2
Q9KUC6	dksA	-1,6	3,5
Q9KL40	hutX	-1,6	2,6
Q9KLQ1	VC_A0691	-1,6	3,4
Q9KKW6	lldD	-1,6	3,4
P0C6Q4	recN	-1,6	3,6
Q9KU32	VC_0697	-1,6	3,6
Q9KQP7	VC_1951	-1,6	2,8
Q9KVT4	hemF	-1,6	3,5

Q9KRL3	VC_1624	-1,6	3,4
Q9KV05	VC_0353	-1,6	3,6
Q9KKM6	VC_A1077	-1,6	3,6
P65128	infA	-1,6	3,6
Q9KRX5	VC_1508	-1,6	3,7
Q9KLW8	tal	-1,6	3,6
Q9KKM8	VC_A1075	-1,6	3,4
Q9KP50	lptA	-1,6	3,2
Q9KQL4	rnd	-1,6	3,3
Q9KL57	VC_A0891	-1,6	3,3
Q9KQ09	VC_2198	-1,6	2,8
Q9KNA7	VC_A0058	-1,6	3,3
Q9KRG4	VC_1678	-1,6	3,4
Q9KQ66	VC_2137	-1,6	3,5
Q9KRK0	rplY	-1,6	3,5
Q9KPL8	deoA	-1,6	3,0
Q9KLT4	VC_A0657	-1,6	3,4
Q9KPT0	VC_2282	-1,6	3,0
Q9KPR6	VC_2300	-1,6	3,2
Q9KRE5	VC_1697	-1,6	3,5
Q9KVG0	VC_0186	-1,6	3,6
Q9KN66	VC_A0099	-1,6	3,6
Q9KS30	VC_1431	-1,6	2,8
Q9KUQ2	VC_0463	-1,6	3,3
Q9KS29	ttcA	-1,6	3,3
Q9KKR9	VC_A1032	-1,6	3,7
Q9KT84	luxO	-1,6	3,5
Q9KN99	VC_A0066	-1,6	2,3
Q9KMX4	VC_A0192	-1,6	3,4
Q9KRC7	mukE	-1,6	3,6
Q9KKL6	VC_A1087	-1,6	3,4
Q9KQD3	VC_2067	-1,6	3,3
Q9KPC9	VC_2443	-1,6	3,4
Q9KVD3	lpxL	-1,6	3,0
O52043	exbB1	-1,6	3,1
Q9KRS3	VC_1563	-1,6	3,3
Q9KPX6	gloB	-1,6	1,9
Q9KPZ9	VC_2208	-1,6	3,5
Q9KSD0	VC_1328	-1,6	2,4
Q9KNI0	fadA	-1,6	2,7
Q9KRX4	cobB	-1,6	2,2

Q9KMW9	guaC	-1,6	3,3
P52022	dnaE	-1,6	3,2
Q9KQD5	VC_2065	-1,6	2,6
Q9KU78	truB	-1,6	3,5
Q9KL75	VC_A0872	-1,7	2,8
Q9KU62	VC_0661	-1,7	2,2
Q9KLH1	VC_A0773	-1,7	3,2
Q9KQ74	VC_2127	-1,7	3,4
Q9KNF3	malT	-1,7	3,6
Q9KPS9	VC_2283	-1,7	1,9
Q9KNP7	VC_2684	-1,7	3,5
Q9KRK5	rsuA	-1,7	3,1
Q9KVG6	VC_0180	-1,7	3,0
Q9KVL6	dapF	-1,7	3,4
Q9KS71	lplA	-1,7	3,7
Q9KTT5	VC_0803	-1,7	3,2
Q9KRU4	norM	-1,7	3,1
Q9KQP1	VC_1957	-1,7	3,1
Q9KR77	VC_1766	-1,7	3,4
Q9KTE9	VC_0953	-1,7	3,3
Q9KRW1	VC_1523	-1,7	3,2
Q9KNG5	rsmG	-1,7	3,4
Q9KQB1	VC_2089	-1,7	3,3
Q9KRQ8	VC_1578	-1,7	2,8
Q9KN98	VC_A0067	-1,7	2,8
Q9KR75	VC_1768	-1,7	3,5
Q9KPP4	recC	-1,7	3,4
H9L4Q9	VC_2451	-1,7	3,6
Q9KQ03	VC_2204	-1,7	3,4
Q9KSV6	VC_1150	-1,7	3,3
Q9KN35	VC_A0130	-1,7	3,3
Q9KT10	VC_1095	-1,7	2,5
Q9KV22	gpmI	-1,7	3,8
Q9KMW4	rhlE	-1,7	3,0
Q9KUP9	VC_0466	-1,7	3,4
Q9KM40	VC_A0549	-1,7	3,2
Q9KUX4	cysJ	-1,7	2,4
Q9KNB2	deoD2	-1,7	2,4
Q9KPQ5	VC_2311	-1,7	3,0
Q9KRU1	VC_1543	-1,7	3,1
Q9KQC5	VC_2075	-1,7	3,1

Q9KSM5	cdd	-1,7	3,1
Q9KPV0	glnD	-1,7	3,4
Q9KT04	VC_1101	-1,7	2,7
Q9KQZ4	VC_1853	-1,7	3,6
Q9KKN0	VC_A1072	-1,7	3,4
Q9KUQ9	VC_0456	-1,7	3,2
Q9KMP5	gcvH	-1,7	3,6
Q9F855	rluE	-1,7	3,5
Q9KQU7	nhaB	-1,7	3,3
Q9KS93	queC	-1,7	2,6
Q9KP83	leuA	-1,7	3,6
Q9KPB2	rnc	-1,7	3,4
Q9KRB5	glgC1	-1,7	3,3
Q9KU93	VC_0630	-1,7	3,0
Q9KRY5	VC_1498	-1,7	3,0
Q9KNJ0	VC_2748	-1,7	3,6
Q9KQW4	VC_1884	-1,7	3,0
Q9KNI1	fadB	-1,7	2,0
Q9KLB3	VC_A0833	-1,7	2,7
Q9KV12	miaA	-1,7	2,9
Q9KPA8	VC_2465	-1,7	3,6
Q9KSI4	VC_1272	-1,8	2,9
Q9KUK6	VC_0512	-1,8	3,5
Q9KVG8	capV	-1,8	2,8
Q9KVH9	rep	-1,8	3,5
Q9KUS5	VC_0440	-1,8	2,8
Q9KQW9	msbA	-1,8	3,4
Q9KNQ2	rpmE	-1,8	3,2
Q9KM14	VC_A0575	-1,8	3,6
Q9KKN5	VC_A1067	-1,8	3,4
Q9KLC6	groL2	-1,8	3,0
Q9KKJ5	tmcA	-1,8	3,0
Q9KSK7	VC_1249	-1,8	3,7
Q9KQK1	VC_1997	-1,8	2,8
Q9KMA2	VC_A0475	-1,8	3,7
Q9KRH8	VC_1664	-1,8	3,0
Q9KS94	queE	-1,8	3,4
Q9KT58	fadJ	-1,8	3,5
Q9KPF1	VC_2417	-1,8	3,2
Q9KLQ3	VC_A0689	-1,8	3,4
Q9KSQ3	hutU	-1,8	2,4

Q9KMZ5	VC_A0171	-1,8	3,6
Q9KNE0	VC_A0025	-1,8	3,7
Q9KLV4	VC_A0637	-1,8	3,3
O66108	thyA	-1,8	3,3
Q9KPP0	VC_2326	-1,8	3,3
Q9KU02	VC_0728	-1,8	2,9
Q9KQ76	VC_2125	-1,8	3,6
Q9KRV1	VC_1533	-1,8	3,7
Q9KTE5	VC_0957	-1,8	3,2
Q9KPD2	VC_2440	-1,8	2,9
Q9KVF2	VC_0194	-1,8	2,9
Q9KQC2	VC_2078	-1,8	3,5
Q9KLY4	VC_A0607	-1,8	3,5
Q9KNL4	bioH	-1,8	3,5
Q9KTD1	ligA	-1,8	3,9
Q9KTF8	lipB	-1,8	3,3
Q9KU37	nagZ	-1,8	3,4
Q9KKR6	VC_A1035	-1,8	3,8
Q9KQ10	VC_2197	-1,8	3,7
Q9KSG7	VC_1289	-1,8	3,7
Q9KQ21	pth	-1,8	3,6
Q9KQ38	VC_2166	-1,8	3,4
Q9KRG9	VC_1673	-1,8	3,6
Q9KVP8	plsB	-1,9	3,2
Q9KQE2	VC_2058	-1,9	3,5
Q9KPD5	VC_2435	-1,9	3,6
Q9KP82	leuB	-1,9	3,4
Q9KML4	VC_A0323	-1,9	3,6
Q9KNP9	metJ	-1,9	3,2
Q9KTH8	VC_0924	-1,9	3,1
Q9KMZ1	VC_A0175	-1,9	3,5
Q9KSL4	astE	-1,9	3,4
Q9KVD6	rph	-1,9	3,7
Q9KL02	VC_A0948	-1,9	3,4
Q9KN45	VC_A0120	-1,9	3,5
Q9KLJ3	fabH2	-1,9	3,7
Q9KRQ9	VC_1577	-1,9	3,5
Q9KUU5	VC_0420	-1,9	3,0
Q9KTN8	VC_0863	-1,9	2,6
Q9KU08	ppx	-1,9	3,6
Q9KQM1	VC_1977	-1,9	3,8

Q9KUB1	VC_0612	-1,9	3,5
Q9KKY0	ppnP	-1,9	3,2
Q9KU34	VC_0695	-1,9	3,5
Q9KT78	VC_1027	-1,9	3,5
P0C6P8	VC_0850	-1,9	2,9
Q9KSB7	VC_1341	-1,9	3,6
Q9KSN9	VC_1217	-1,9	2,9
Q9KSD3	VC_1325	-1,9	3,4
Q9KQ44	VC_2160	-1,9	2,3
Q9KQY8	VC_1859	-1,9	3,3
Q9KL55	VC_A0893	-1,9	3,4
Q9KNV3	VC_2627	-1,9	3,6
Q9KM18	VC_A0571	-1,9	3,6
Q9KVI3	VC_0163	-1,9	3,7
Q9KNV5	VC_2625	-1,9	3,7
Q9KUS4	apaH	-1,9	3,3
Q9KVF6	VC_0190	-1,9	3,1
Q9KLL4	VC_A0729	-1,9	3,6
Q9KU39	murQ2	-2,0	3,7
Q9KUS1	pdxA	-2,0	3,3
Q9KMG5	higB-1	-2,0	2,9
Q9KU38	anmK	-2,0	3,7
Q9KPW4	lpxA	-2,0	3,9
Q9KL87	VC_A0859	-2,0	3,3
Q9KT95	VC_1008	-2,0	3,7
Q9KVV5	VC_0033	-2,0	2,6
H9L4S8	VC_1344	-2,0	3,5
Q9KL10	VC_A0940	-2,0	3,5
Q9KP32	VC_2547	-2,0	2,9
Q9KSZ3	bioF	-2,0	3,8
Q9KP37	mpl	-2,0	3,1
Q9KVL9	VC_0122	-2,0	3,8
Q9KQN2	VC_1966	-2,0	3,2
Q9KTW6	VC_0764	-2,0	3,9
Q9KSS6	VC_1180	-2,0	3,1
Q9KVU4	fmt	-2,0	3,6
Q9KKM4	pdxH	-2,0	3,6
Q9KNW3	VC_2617	-2,0	3,4
Q9KUL9	VC_0496	-2,0	3,3
P0C6D8	tcpI	-2,0	3,5
Q9KLC7	groS2	-2,0	3,6

Q9KPI6	btuF	-2,0	3,2
Q9KMP4	glyA2	-2,0	3,3
Q9KQX5	VC_1872	-2,0	3,9
O34238	murJ	-2,0	2,5
Q9KR64	siaP	-2,0	3,4
Q9KRR3	fumC	-2,1	3,9
Q9KUY0	fur	-2,1	3,5
Q9KND6	VC_A0029	-2,1	2,9
Q9KNW4	astD	-2,1	3,6
Q9KVG9	vspR	-2,1	3,7
Q9KRH2	clsA	-2,1	2,8
O52044	exbD1	-2,1	3,9
Q9KQZ0	VC_1857	-2,1	3,4
Q9KNI9	ntrC	-2,1	3,6
Q9KUA3	VC_0620	-2,1	3,5
Q9KM19	VC_A0570	-2,1	3,9
Q9KPZ5	VC_2217	-2,1	3,1
Q9KNL1	VC_2721	-2,1	3,5
Q9KVH5	VC_0171	-2,1	3,5
Q9KRP8	VC_1588	-2,1	2,8
Q9KUC7	gluQ	-2,1	3,7
Q9KKW9	VC_A0981	-2,1	3,0
Q9KM22	VC_A0567	-2,1	3,6
Q9KPD8	VC_2432	-2,1	3,2
P0C6Q9	bamE	-2,1	3,1
Q9KSP3	VC_1213	-2,1	3,5
Q9KKT9	VC_A1011	-2,2	2,9
P52692	nhaR	-2,2	3,2
Q9KMX7	VC_A0189	-2,2	3,9
Q9KQZ6	VC_1851	-2,2	3,9
Q9KNN0	VC_2702	-2,2	3,5
Q9KPV1	map	-2,2	2,4
Q9KT92	nth	-2,2	3,9
Q9KP63	VC_2513	-2,2	2,6
O85187	nhaA	-2,2	3,3
Q9KUB2	VC_0611	-2,2	3,7
Q9KPA1	VC_2472	-2,2	3,8
Q9KLS8	VC_A0663	-2,2	3,4
Q9KLX4	VC_A0617	-2,2	3,7
Q9KPK8	ung	-2,2	3,4
Q9KLR8	VC_A0673	-2,2	3,9

Q9KTI8	VC_0914	-2,2	3,4
Q9KVY0	VC_0008	-2,2	3,9
Q9KSS8	VC_1178	-2,3	3,5
Q9KPI9	VC_2378	-2,3	2,3
Q9KMA5	higA-2	-2,3	3,4
Q9KT61	VC_1044	-2,3	3,3
Q9KRJ7	VC_1643	-2,3	3,6
Q9KS38	VC_1423	-2,3	2,9
Q9KST6	trpB	-2,3	3,6
Q9KQK0	msrB	-2,3	3,7
Q9KM66	cqsS	-2,3	3,6
Q9KS12	rtxA	-2,3	3,3
Q9KND3	VC_A0032	-2,3	3,5
Q9KTL0	VC_0892	-2,3	3,4
Q9KSE1	VC_1316	-2,3	2,8
Q9KMD8	VC_A0423	-2,3	3,4
Q9KSV4	VC_1152	-2,3	2,3
H9L4P1	VC_0259	-2,3	3,0
Q9KSR0	VC_1196	-2,3	3,5
Q9KPZ0	VC_2222	-2,3	3,9
P24003	toxS	-2,3	3,5
Q9KM05	VC_A0584	-2,3	4,1
Q9KPG4	mraY	-2,3	3,5
Q9KPH6	VC_2392	-2,3	3,4
P0C6C2	epd	-2,3	3,4
Q9KQK2	VC_1996	-2,3	3,5
Q9KRL6	VC_1621	-2,3	3,8
Q9KLB6	VC_A0830	-2,4	3,3
Q9KP49	VC_2529	-2,4	3,4
Q9KQC3	VC_2077	-2,4	3,9
Q9KLZ8	VC_A0591	-2,4	3,8
Q9KRP1	galK	-2,4	3,4
Q9KVC1	VC_0225	-2,4	2,4
Q9KRK3	VC_1637	-2,4	3,4
Q9KV72	VC_0285	-2,4	3,7
Q9KV18	rsgA	-2,4	3,5
Q9KNM1	recG	-2,4	3,6
Q9KT65	VC_1040	-2,4	3,4
Q9KQW3	VC_1885	-2,5	3,5
Q9KL32	VC_A0917	-2,5	3,6
Q9KU85	VC_0638	-2,5	3,3

Q9KPD6	VC_2434	-2,5	3,6
Q9KKX2	VC_A0978	-2,5	3,4
Q9KQZ2	dinG	-2,5	2,2
Q9KTB3	rfaH	-2,5	3,4
P45782	epsL	-2,5	4,0
Q9KPD1	VC_2441	-2,5	3,9
Q9KRQ1	VC_1585	-2,5	3,6
Q9KTL6	VC_0886	-2,5	3,5
Q9KKS5	nagB	-2,5	4,0
Q9KVC5	mutM	-2,5	3,7
Q9KNS4	VC_2657	-2,5	4,0
Q9KPN1	VC_2335	-2,5	4,1
Q9KPS6	VC_2286	-2,5	3,7
Q9KTI9	VC_0913	-2,5	3,4
Q9KQU8	fadR	-2,5	3,9
Q9KL16	cspV	-2,5	2,5
Q9KR15	VC_1832	-2,6	3,2
Q9KL77	VC_A0870	-2,6	3,1
Q9KP22	VC_2557	-2,6	2,6
Q9KMW5	VC_A0201	-2,6	3,5
Q9KPU0	nrdR	-2,6	3,6
Q9KPM6	VC_2341	-2,6	3,7
Q9KT93	gloA	-2,6	3,9
Q9KVW9	VC_0019	-2,6	3,0
Q9KM65	cqsA	-2,6	4,1
Q9KPS0	bolA	-2,6	3,5
Q9KVQ7	rmuC	-2,6	4,0
Q56632	vibA	-2,6	3,4
Q9KLT7	VC_A0654	-2,6	3,2
Q9KQD0	VC_2070	-2,6	3,9
Q9KVW2	VC_0026	-2,7	3,7
P58093	parD	-2,7	3,2
Q9KUB9	VC_0603	-2,7	3,4
Q9KTW4	xseA	-2,7	3,9
Q9KUW0	VC_0399	-2,7	2,7
Q9KS17	VC_1444	-2,7	4,1
Q9KUX0	VC_0389	-2,7	3,3
Q9KP81	leuC	-2,7	2,8
Q9KQX9	VC_1868	-2,7	3,5
Q9X6N4	murE	-2,8	3,8
Q9KTW8	bamB	-2,8	2,9

Q9KNG9	atpB	-2,8	2,4
Q9KRG7	VC_1675	-2,9	3,5
Q9KLA9	VC_A0837	-2,9	4,0
O07898	vibC	-2,9	3,8
Q9KS82	VC_1377	-2,9	3,3
Q9KQD1	flhA	-3,0	3,4
Q9KQX3	VC_1874	-3,0	4,0
Q9KV66	dusB	-3,0	3,2
Q9KPC7	VC_2445	-3,0	3,5
Q9KUQ3	VC_0462	-3,0	3,5
Q9KVU3	def1	-3,0	3,5
Q9KTK9	VC_0893	-3,0	4,0
Q9KLE6	VC_A0800	-3,0	2,4
Q9KU52	VC_0672	-3,1	3,0
Q9KS11	VC_1453	-3,1	4,1
Q9KNW5	VC_2615	-3,1	3,3
Q9KVV9	ilvE	-3,2	2,8
Q9KQ23	ispE	-3,2	3,7
Q9KLD8	VC_A0808	-3,3	3,7
Q9KSF3	VC_1303	-3,3	3,4
Q9KPR9	rlmG	-3,4	2,9
Q9KL64	VC_A0883	-3,4	3,8
Q9KPW5	lpxB	-3,5	3,7
Q9KL48	VC_A0900	-3,5	4,1
Q9KRG3	VC_1679	-3,5	4,2
Q9KQM2	menF	-3,6	2,7
Q9KPW3	fabZ	-3,7	2,3
Q9KLT3	VC_A0658	-3,7	2,2
Q9KPJ1	VC_2376	-3,7	3,0
Q9KVW1	ilvA	-3,7	4,0
O32445	nagA	-4,0	3,0
Q9KLC8	VC_A0818	-4,0	2,8
O52042	tonB	-4,0	2,7
Q9KM87	VC_A0495	-4,0	2,7
Q9KPC6	cca	-4,0	2,6
Q9KLK5	VC_A0738	-4,0	2,6
O68845	rpmI	-4,0	2,5
Q9KQ71	VC_2130	-4,0	2,5
Q9KM94	VC_A0487	-4,0	2,5
Q9KRD8	metE	-4,0	2,5
Q9KPK9	VC_2358	-4,0	2,5

Q9KLM3	VC_A0720	-4,0	2,5
Q9KSE0	VC_1317	-4,0	2,4
Q9KQ73	VC_2128	-4,0	2,4
Q9KMD9	VC_A0422	-4,0	2,4
Q9KMC1	VC_A0443	-4,0	2,4
Q9KTS5	VC_0813	-4,0	2,3
Q9KRH4	VC_1668	-4,0	2,3
Q9KPY0	VC_2232	-4,0	2,3
Q9KLN4	VC_A0709	-4,0	2,3
Q9KUX2	cysH	-4,0	2,3
Q9KSI8	VC_1268	-4,0	2,3
Q9KQA6	VC_2094	-4,0	2,3
Q9K2P2	VC_A0292	-4,0	2,3
Q9KMI2	VC_A0368	-4,0	2,3
Q9KLR5	napD	-4,0	2,3
Q9KTS9	VC_0809	-4,0	2,3
Q9KRI8	VC_1653	-4,0	2,3
Q9KPY1	VC_2231	-4,0	2,3
Q9KV69	edd	-4,0	2,2
Q9KSN0	tpm	-4,0	2,2
Q9KQC7	VC_2073	-4,0	2,2
Q9KMK9	VC_A0330	-4,0	2,2
Q9KKR5	VC_A1037	-4,0	2,2
Q9KRM5	metAS	-4,0	2,2
Q9KU45	VC_0684	-4,0	2,2
Q9KQ04	VC_2203	-4,0	2,2
Q9KM03	VC_A0586	-4,0	2,2
Q9KQI2	tmk	-4,0	2,2
Q9KVL3	VC_0129	-4,0	2,2
Q9KSS9	VC_1177	-4,0	2,2
Q9KMM4	VC_A0307	-4,0	2,2
Q9KKZ7	VC_A0953	-4,0	2,2
Q9KU53	rppH	-4,0	2,1
Q9KRS9	VC_1557	-4,0	2,1
Q9KQ11	VC_2196	-4,0	2,1
Q9KM04	VC_A0585	-4,0	2,1
Q9KSZ2	bioC	-4,0	2,1
Q9KVR3	VC_0076	-4,0	2,1
Q9KQJ2	VC_2006	-4,0	2,1
Q9KMN0	VC_A0301	-4,0	2,1
Q9KL01	VC_A0949	-4,0	2,1

Q9KU66	VC_0657	-4,0	2,1
Q9KRT1	VC_1555	-4,0	2,1
Q9KQ16	VC_2191	-4,0	2,1
Q9KM07	VC_A0582	-4,0	2,1
Q9KQK5	VC_1993	-4,0	2,0
Q9KMQ7	VC_A0263	-4,0	2,0
Q9KL45	VC_A0903	-4,0	2,0
Q9KVS1	VC_0068	-4,0	2,0
Q9KT09	VC_1096	-4,0	2,0
Q9KUH9	VC_0542	-4,0	2,0
Q9KRX9	VC_1504	-4,0	2,0
Q9KQ28	VC_2176	-4,0	2,0
Q9KM08	VC_A0581	-4,0	2,0
Q9KT81	moaA	-4,0	2,0
Q9KQL9	VC_1979	-4,0	2,0
Q9KNA9	VC_A0056	-4,0	2,0
Q9KLB7	VC_A0829	-4,0	2,0
Q9KVV4	srkA	-4,0	2,0
Q9KUI0	cysA	-4,0	2,0
Q9KS73	VC_1386	-4,0	2,0
Q9KQ45	VC_2159	-4,0	2,0
Q9KM28	VC_A0561	-4,0	2,0
Q9KP71	rluA	-4,0	2,0
Q9KT86	rnfA	-4,0	2,0
Q9KVV7	VC_0031	-4,0	2,0
Q9KQR3	VC_1935	-4,0	2,0
Q9KUN3	argP	-4,0	1,9
Q9KQ63	fliD	-4,0	1,9
Q9KS75	VC_1384	-4,0	1,9
Q9ZHV9	exbD2	-4,0	1,9
Q9KTC1	VC_0982	-4,0	1,9
Q9KQX1	VC_1876	-4,0	1,9
Q9KUP8	VC_0467	-4,0	1,9
Q9KS88	VC_1371	-4,0	1,9
Q9KTM4	rpmE2	-4,0	1,9
Q9KUV0	VC_0414	-4,0	1,9
Q9KMY4	VC_A0182	-4,2	4,6
Q9KR04	VC_1843	-4,2	4,0
Q9KPL9	deoB	-4,3	4,1
Q9KSU7	serC	-4,3	4,4
Q9KM24	VC_A0565	-4,4	3,9

Q9KQA7	VC_2093	-4,6	4,6
Q9KRJ9	VC_1641	-4,9	4,6
Q9KV75	VC_0281	-5,0	4,2
Q9KUV8	VC_0404	-5,0	2,5
Q9KRD9	VC_1703	-5,6	3,2

Table S4: TN-seq

T0 *ΔdusB* vs WT (425 genes)			
Orf	Name	log2FC	Adj, p-value (log10)
VC_0927	vpsK	-4,9	1,5
VC_A0260	-	-4,9	1,4
VC_A0184	capB	-4,2	1,3
VC_1801	-	-4,1	1,5
VC_1546	exbB2	-3,9	1,5
VC_1619	-	-3,9	1,7
VC_A0992	-	-3,8	1,9
VC_0057	-	-3,7	1,4
VC_A0139	-	-3,7	1,6
VC_2560	cysD	-3,6	3,7
VC_2160	bcp	-3,6	1,6
VC_1116	-	-3,4	1,6
VC_2361	grcA	-3,4	1,9
VC_1421	-	-3,4	2,3
VC_1676	-	-3,4	2,6
VC_0384	cysJ	-3,1	3,2
VC_0385	cysI	-3,1	2,9
VC_A0247	ulaR	-2,9	3,3
VC_1236	msrB	-2,9	1,4
VC_0974	cueR	-2,8	2,3
VC_A0334	-	-2,8	1,5
VC_0493	-	-2,8	3,4
VC_1270	-	-2,8	1,4
VC_A0627	-	-2,8	1,6
VC_A0825	ectA	-2,8	1,3
VC_2704	-	-2,7	2,2
VC_1894	lpoB	-2,7	2,6
VC_A0460	-	-2,6	2,9
VC_1139	hisI	-2,6	2,1
VC_0451	-	-2,5	1,4
VC_2385	ret	-2,5	1,9
VC_1245	-	-2,4	1,6
VC_1504	-	-2,4	2,2
VC_2264	-	-2,3	1,3
VC_2561	sumT	-2,3	1,8
VC_0386	cysH	-2,2	1,4
VC_A0408	-	-2,2	1,8
VC_0819	aldA	-2,2	1,7

VC_A0501	-	-2,2	1,5
VC_0055	hemF	-2,1	1,8
VC_1169	trpA	-2,1	2,0
VC_0447	djlA	-2,1	1,4
VC_2546	-	-2,1	1,5
VC_A0708	pykA	-2,1	2,5
VC_2553	zrgC	-2,0	3,0
VC_1034	udp	-2,0	1,8
VC_A0464	-	-2,0	1,6
VC_A0618	moeB	-1,9	1,6
VC_A0854	-	-1,7	2,1
VC_0586	can	-1,7	2,2
VC_A0193	-	-1,6	1,7
VC_0790	citB	-1,6	1,4
VC_1182	trxB	-1,5	1,3
VC_A0590	-	-1,4	1,7
VC_0662	braB	-1,4	1,8
VC_0425	-	-1,3	1,4
VC_0779	fepC	-1,3	1,6
VC_A0200	-	-1,3	1,6
VC_2394	secA	0,5	1,3
VC_2373	gltA	0,7	1,3
VC_1451	rtxA	0,7	2,4
VC_A0849	-	0,8	3,0
VC_2077	feoB	0,8	1,4
VC_A0371	-	0,8	1,7
VC_2621	xds	0,8	1,4
VC_1478	-	0,8	1,6
VC_A0065	-	0,9	1,4
VC_2364	thrA	0,9	1,4
VC_0225	waaC	0,9	1,4
VC_1789	-	0,9	1,9
VC_0398	-	0,9	1,3
VC_1650	prt	0,9	1,3
VC_1321	-	1,0	1,6
VC_0156	btuB	1,0	2,4
VC_0393	-	1,0	1,6
VC_0137	cdgJ	1,0	1,5
VC_2080	tfos	1,0	2,0
VC_2451	relA	1,0	1,3
VC_1766	-	1,1	1,8

VC_1211	-	1,1	1,5
VC_0298	acs	1,1	1,6
VC_A0865	hap	1,1	1,5
VC_A1085	-	1,1	1,4
VC_1757	vexD	1,1	1,8
VC_0394	uvrA	1,1	1,9
VC_2242	tilS	1,1	2,0
VC_2738	pck	1,1	1,6
VC_0048	smf	1,1	1,5
VC_0914	vexH	1,1	2,0
VC_A0510	-	1,1	1,7
VC_0992	-	1,1	1,4
VC_A1029	-	1,1	1,5
VC_0188	prlC	1,2	1,6
VC_0390	metH	1,2	3,3
VC_1469	cri	1,2	2,2
VC_1709	-	1,2	1,7
VC_2600	-	1,2	1,6
VC_0921	vpsE	1,2	1,6
VC_1475	cri	1,2	2,0
VC_0270	mak	1,2	1,4
VC_A0091	-	1,2	1,3
VC_1770	-	1,2	1,7
VC_2135	fleR	1,2	1,6
VC_2338	lacZ	1,2	#NUM!
VC_2338	lacZ	1,2	3,5
VC_2371	-	1,2	1,4
VC_2646	ppc	1,2	2,3
VC_2438	glnE	1,2	2,2
VC_A1069	-	1,2	1,6
VC_0069	-	1,2	2,3
VC_A0522	cqsS	1,2	1,7
VC_2235	-	1,2	1,9
VC_2285	cdgL	1,2	1,5
VC_A0044	-	1,2	1,5
VC_1767	-	1,2	1,5
VC_2356	-	1,2	1,7
VC_2547	-	1,2	2,5
VC_A0177	-	1,2	1,6
VC_0629	vexF	1,3	1,9
VC_A0120	-	1,3	3,2

VC_A0697	cdgD	1,3	1,4
VC_0184	-	1,3	1,3
VC_2162	perM	1,3	1,4
VC_2638	-	1,3	1,4
VC_2705	-	1,3	2,2
VC_A0863	cef	1,3	2,3
VC_A0939	-	1,3	1,3
VC_0302	-	1,3	1,4
VC_1446	rtxE	1,3	1,4
VC_2161	mcpX	1,3	1,8
VC_1501	-	1,3	1,8
VC_2683	metB	1,3	1,4
VC_0170	gsiA	1,3	2,1
VC_A0008	-	1,3	1,9
VC_0839	tcpJ	1,3	2,4
VC_1620	-	1,3	#NUM!
VC_1620	-	1,3	#NUM!
VC_2490	leuA	1,3	1,7
VC_A0151	-	1,3	1,5
VC_1047	fadJ	1,3	1,4
VC_2750	-	1,3	3,0
VC_0717	-	1,3	1,3
VC_1898	-	1,3	1,3
VC_2068	-	1,3	1,8
VC_A0141	-	1,3	1,5
VC_0972	chiP	1,4	1,7
VC_1571	cioA	1,4	1,4
VC_A0867	ompW	1,4	1,4
VC_0930	rbmC	1,4	2,5
VC_2133	-	1,4	1,7
VC_A0772	tyrP	1,4	1,4
VC_0535	mutS	1,4	1,9
VC_0019	avtA	1,4	1,3
VC_A0728	-	1,4	1,9
VC_A0350	blc	1,4	1,4
VC_0602	mrcB	1,4	2,0
VC_2538	-	1,4	1,6
VC_A0020	vasX	1,4	2,8
VC_2506	rapA	1,4	2,4
VC_2759	fadA	1,4	1,4
VC_0040	hlyIII	1,4	1,8

VC_A0077	-	1,4	1,4
VC_0251	-	1,4	2,5
VC_2187	flaD	1,4	1,4
VC_0090	vcmD	1,5	1,6
VC_1979	-	1,5	1,4
VC_2380	-	1,5	1,4
VC_0080	-	1,5	1,9
VC_0551	oadB-1	1,5	1,5
VC_2599	rnr	1,5	2,4
VC_A0068	-	1,5	1,6
VC_0350	hflC	1,5	1,5
VC_1931	-	1,5	1,5
VC_A0018	-	1,5	1,5
VC_0558	-	1,5	1,4
VC_0793	oadA	1,5	1,5
VC_1147	-	1,5	1,5
VC_A0705	-	1,5	2,1
VC_0630	shyC	1,5	1,7
VC_0130	-	1,5	2,8
VC_0199	vcaM	1,5	2,0
VC_0231	wavG	1,5	4,0
VC_0312	fre	1,5	#NUM!
VC_0547	lysC	1,5	1,3
VC_0924	vpsH	1,5	1,4
VC_A0083	-	1,5	1,5
VC_0250	-	1,5	1,3
VC_2197	flgE	1,5	1,9
VC_2727	epsJ	1,5	1,8
VC_0044	rsmB	1,6	1,4
VC_0286	-	1,6	1,6
VC_1866	pflB	1,6	2,1
VC_A0768	rhlE	1,6	1,4
VC_A1112	-	1,6	1,6
VC_0154	trmA	1,6	1,7
VC_1260	-	1,6	1,5
VC_A0687	-	1,6	1,5
VC_1565	-	1,6	1,3
VC_0168	cycB	1,6	2,2
VC_1665	-	1,6	1,4
VC_2564	dbpA	1,6	1,4
VC_A0160	mtr	1,6	1,7

VC_A0744	glpK	1,6	1,9
VC_A1015	nhaD	1,6	2,4
VC_1265	-	1,6	1,4
VC_A0066	-	1,6	1,4
VC_0492	-	1,6	1,7
VC_A1102	-	1,6	2,0
VC_0616	-	1,6	2,0
VC_1754	pqiB	1,6	1,8
VC_0058	-	1,6	1,7
VC_0937	vpsO	1,6	3,2
VC_1432	ttcA	1,6	1,7
VC_A0074	cdgA	1,6	1,3
VC_A1018	-	1,6	1,5
VC_0590	-	1,7	1,4
VC_1798	-	1,7	1,6
VC_2487	-	1,7	1,4
VC_0488	-	1,7	2,2
VC_0617	-	1,7	1,4
VC_2060	-	1,7	1,4
VC_A0317	blc	1,7	1,7
VC_0732	oxyR	1,7	1,4
VC_0479	-	1,7	1,8
VC_0821	-	1,7	2,1
VC_0736	aceA	1,7	1,7
VC_2262	glnD	1,7	1,3
VC_A0063	ptrB	1,7	2,4
VC_1743	-	1,7	1,3
VC_2061	-	1,7	1,8
VC_0910	treB	1,7	1,9
VC_1562	-	1,7	2,5
VC_A0246	ulaA	1,7	2,4
VC_A0443	blc	1,7	1,8
VC_1085	-	1,8	1,6
VC_A0754	-	1,8	1,3
VC_2688	glpX	1,8	1,5
VC_A0685	-	1,8	1,5
VC_A0699	glgC	1,8	1,9
VC_A1095	-	1,8	1,4
VC_0278	-	1,8	2,8
VC_A0706	-	1,8	1,5
VC_0769	-	1,8	2,6

VC_2694	sodA	1,8	1,6
VC_A0993	nemA	1,8	1,8
VC_A1088	-	1,8	1,3
VC_0205	-	1,8	1,4
VC_2717	-	1,8	1,5
VC_1995	ansA	1,8	1,6
VC_A0829	acsA	1,8	1,5
VC_1591	alsO	1,8	1,4
VC_A0936	-	1,8	1,6
VC_A0963	-	1,8	1,4
VC_0039	-	1,8	1,3
VC_A0658	-	1,8	1,9
VC_A0848	-	1,8	1,6
VC_0402	mshD	1,8	3,2
VC_A0067	-	1,8	1,7
VC_A0270	dacC	1,9	2,5
VC_A0960	-	1,9	2,4
VC_A0351	-	1,9	1,5
VC_0421	-	1,9	1,9
VC_1039	-	1,9	1,3
VC_1782	nanK	1,9	1,8
VC_1373	-	1,9	3,4
VC_1442	ctaD	1,9	2,4
VC_2636	oxyR	1,9	1,3
VC_1855	dinG	1,9	1,9
VC_1941	-	1,9	2,4
VC_A0459	-	1,9	1,6
VC_0331	nudC	1,9	1,4
VC_1389	-	1,9	1,5
VC_0265	-	2,0	1,8
VC_A0013	malP	2,0	2,4
VC_A0596	-	2,0	1,5
VC_0054	rimN	2,0	1,7
VC_0401	mshK	2,0	1,4
VC_A0650	-	2,0	1,9
VC_0173	-	2,0	1,7
VC_A0636	-	2,0	1,9
VC_A0818	-	2,0	1,6
VC_2624	gph	2,0	1,9
VC_1210	-	2,0	1,3
VC_2059	cheW	2,0	2,0

VC_1282	chbC	2,1	1,9
VC_1379	-	2,1	1,5
VC_A0983	-	2,1	2,6
VC_1046	fadI	2,1	2,8
VC_0242	rfbB	2,1	2,5
VC_A0763	ravA	2,1	#NUM!
VC_A0860	-	2,1	2,6
VC_0132	-	2,1	3,5
VC_0600	-	2,1	1,6
VC_0791	-	2,1	1,5
VC_2030	rne	2,1	1,8
VC_A1041	-	2,1	2,4
VC_1286	chbR	2,1	3,5
VC_1232	-	2,1	2,1
VC_2346	-	2,1	1,3
VC_A0136	glpQ	2,1	2,3
VC_1342	-	2,1	1,6
VC_A0019	-	2,1	1,5
VC_1877	lpxK	2,1	1,8
VC_0029	ilvE	2,1	2,6
VC_1910	miaE	2,1	2,5
VC_A0779	-	2,1	1,8
VC_0153	-	2,2	1,7
VC_0245	rfbG	2,2	1,4
VC_0968	cysK	2,2	1,6
VC_2534	-	2,2	2,8
VC_2712	xanP	2,2	1,6
VC_1427	potB	2,2	1,4
VC_2121	-	2,2	2,7
VC_A0608	yjjG	2,2	1,6
VC_A0676	-	2,2	1,3
VC_0633	ompU	2,2	2,7
VC_A0751	fabHA	2,2	1,5
VC_0099	glpG	2,2	2,0
VC_0086	tatA	2,2	3,0
VC_A0762	-	2,2	#NUM!
VC_1018	uvrB	2,2	3,7
VC_1018	uvrB	2,2	3,5
VC_0691	anmK	2,3	1,7
VC_2270	ribE	2,3	1,6
VC_A0972	-	2,3	2,2

VC_1778	siaQ	2,3	1,5
VC_0136	rhtB	2,3	2,1
VC_0685	ispH	2,3	1,4
VC_1439	ccoP	2,3	3,7
VC_2006	-	2,3	2,4
VC_1199	-	2,3	1,6
VC_2100	-	2,3	1,3
VC_0537	cysM	2,4	1,9
VC_1179	rluB	2,4	1,7
VC_1984	rnd	2,4	1,5
VC_1744	-	2,4	1,9
VC_A0088	-	2,4	2,5
VC_A0397	-	2,4	1,5
VC_0091	-	2,4	1,6
VC_A0785	-	2,4	3,0
VC_A1066	-	2,4	1,5
VC_1695	focA	2,4	3,0
VC_2668	-	2,4	1,6
VC_0866	mltF	2,4	2,2
VC_2112	-	2,4	2,3
VC_2336	ygjH	2,5	2,1
VC_0534	rpoS	2,5	2,7
VC_1865	-	2,5	2,2
VC_1861	hisM	2,5	2,9
VC_1903	ftsK	2,5	1,8
VC_1440	ccoQ	2,5	3,2
VC_A1065	-	2,6	1,5
VC_2240	padC	2,6	1,8
VC_2326	yebG	2,6	1,5
VC_A0780	udg	2,6	1,4
VC_1720	-	2,6	1,3
VC_1940	-	2,6	1,4
VC_2382	-	2,6	1,8
VC_A0542	-	2,6	2,0
VC_2054	-	2,6	3,2
VC_2195	flgG	2,6	2,3
VC_2098	-	2,7	2,7
VC_1376	-	2,8	2,7
VC_1436	-	2,8	4,0
VC_1876	-	2,8	2,1
VC_A0338	-	2,8	1,6

VC_0999	truA	2,8	1,7
VC_A0691	phaB	2,8	2,3
VC_1667	-	2,8	1,4
VC_A0179	nupX	2,9	2,7
VC_1631	-	2,9	2,0
VC_0531	surE	2,9	1,8
VC_A0890	-	2,9	1,6
VC_2035	-	3,0	2,2
VC_A0690	phbA	3,0	2,6
VC_1842	-	3,1	1,9
VC_2530	-	3,1	1,5
VC_0246	rfbH	3,2	1,7
VC_2141	flaG	3,2	1,5
VC_1318	ompV	3,2	2,3
VC_A0577	-	3,2	1,6
VC_1305	-	3,3	2,3
VC_0800	-	3,4	1,9
VC_2517	-	3,4	1,5
VC_1089	-	3,4	1,9
VC_1484	rmf	3,4	2,1
VC_A0592	-	3,4	2,7
VC_1315	-	3,4	2,6
VC_0355	-	3,4	2,1
VC_A0504	relB4	3,4	2,6
VC_A0349	relB2	3,5	2,6
VC_1556	-	3,6	1,6
VC_2293	nqrC	3,6	#NUM!
VC_A0927	-	3,6	1,4
VC_A0948	-	3,6	1,7
VC_1607	-	3,7	1,4
VC_2265	pgpA	3,7	1,5
VC_1350	PRXIID	3,7	1,7
VC_A0999	-	3,7	1,9
VC_2292	nqrD	3,8	#NUM!
VC_1633	-	3,8	4,0
VC_2294	nqrB	3,9	#NUM!
VC_0964	crr	3,9	#NUM!
VC_1637	-	4,0	1,5
VC_0966	ptsH	4,0	2,9
VC_A0612	mscL	4,1	1,6
VC_1449	rtxH	4,2	3,7

VC_1755	-	4,2	3,2
VC_1702	-	4,3	1,7
VC_0933	rbmEF	4,4	#NUM!
VC_1079	-	4,6	1,4
VC_1887	csiV	5,0	1,5
VC_1849	ppiB	5,1	1,5
VC_1809	-	5,1	#NUM!
VC_1052	-	5,2	1,4
VC_0583	hapR	5,3	2,1
VC_0583	hapR	5,3	2,1
VC_0848	smpB	5,4	2,5
VC_1130	hns	6,2	1,9
VC_0842	-	6,3	3,5
VC_0818	-	6,6	4,0
VC_0818	-	6,6	4,0
VC_A0445	relB3	6,6	1,7
VC_A0166	cspG	6,9	2,3
VC_1802	-	7,8	1,8
ΔdusB T16 vs T0 (316 genes)			
Orf	Name	log2FC	negative log10 pval
VC_A0184	capB	6,4	1,4
VC_2172	ybaK	4,7	1,4
VC_A1032	-	4,6	2,8
VC_A0247	ulaR	3,9	1,8
VC_A0094	-	3,7	1,9
VC_A0582	-	3,7	1,4
VC_2205	-	3,5	3,5
VC_2385	ret	3,3	2,6
VC_0491	-	3,2	1,7
VC_A0424	-	3,2	1,4
VC_2123	fliP	3,0	3,7
VC_2601	motX	3,0	4,0
VC_A0628	-	3,0	2,3
VC_A1052	-	3,0	1,5
VC_2275	crl	2,9	3,5
VC_2609	-	2,7	1,4
VC_0730	-	2,7	1,3
VC_0790	citB	2,6	1,6
VC_2190	flgL	2,6	4,0
VC_A0470	-	2,6	1,6
VC_1277	-	2,6	1,6

VC_1008	motY	2,3	4,0
VC_0980	-	2,2	1,3
VC_0458	-	2,2	1,4
VC_0534	rpoS	2,2	4,0
VC_2066	fliA	2,1	2,6
VC_A0017	hcp	2,1	1,5
VC_0428	-	2,1	1,9
VC_A0708	pykA	2,1	1,7
VC_0016	-	2,0	1,3
VC_2188	flaA	2,0	3,0
VC_2452	rumA	2,0	2,1
VC_A0387	-	1,9	2,0
VC_1671	metC	1,9	1,6
VC_1719	torR	1,9	1,3
VC_2620	rimO	1,8	1,7
VC_A0308	-	1,8	2,9
VC_2131	-	1,8	1,4
VC_2069	flhA	1,8	3,3
VC_A1090	-	1,8	1,7
VC_0696	tyrA	1,7	1,7
VC_0896	gcvA	1,7	1,6
VC_2137	flrA	1,7	3,7
VC_2062	cheB	1,7	3,0
VC_2355	-	1,6	1,7
VC_A0098	-	1,6	1,3
VC_2627	-	1,6	2,5
VC_1750	-	1,5	1,8
VC_2059	cheW	1,5	2,4
VC_2208	flgT	1,5	2,8
VC_1549	-	1,5	1,8
VC_2136	fleS	1,5	1,7
VC_2135	fleR	1,5	3,7
VC_A0495	-	1,5	1,3
VC_0648	nlpI	1,4	1,6
VC_2130	fliI	1,4	1,8
VC_2121	-	1,3	2,2
VC_0825	tcpI	1,3	1,3
VC_2760	-	1,3	1,4
VC_2120	flhB	1,3	2,0
VC_A0536	-	1,2	1,3
VC_1653	vieS	1,2	1,7

VC_2061	-	1,1	1,5
VC_0723	ppk	1,1	1,8
VC_2191	flgM	1,1	1,4
VC_2140	fliDP	1,0	1,4
VC_2295	nqrA	-1,0	2,8
VC_0156	btuB	-1,0	2,2
VC_2077	feoB	-1,0	1,7
VC_2338	lacZ	-1,0	2,5
VC_2338	lacZ	-1,0	2,5
VC_1491	pyrD	-1,0	1,4
VC_A0123	vgrG3	-1,0	1,3
VC_0747	iscR	-1,1	1,7
VC_0048	smf	-1,1	1,4
VC_2469	nadB	-1,1	1,5
VC_1018	uvrB	-1,1	1,4
VC_1018	uvrB	-1,1	1,4
VC_2088	sdhB	-1,1	1,6
VC_1373	-	-1,1	1,5
VC_0188	prlC	-1,1	1,5
VC_0312	fre	-1,2	2,8
VC_1904	lrp	-1,2	2,3
VC_0914	vexH	-1,2	2,0
VC_0199	vcaM	-1,2	1,4
VC_0602	mrcB	-1,2	1,5
VC_0042	trkH	-1,2	1,3
VC_0132	-	-1,2	1,9
VC_A0638	vexM	-1,2	1,9
VC_A0270	dacC	-1,3	1,5
VC_A0960	-	-1,3	1,4
VC_0535	mutS	-1,3	1,5
VC_0620	cbp	-1,3	1,3
VC_A0785	-	-1,3	1,5
VC_0305	rhlB	-1,3	1,4
VC_0793	oadA	-1,3	1,3
VC_1684	sapF	-1,4	1,7
VC_0795	citS	-1,4	1,4
VC_0717	-	-1,4	1,4
VC_2417	recJ	-1,5	1,7
VC_0958	lnt	-1,5	1,5
VC_0219	rpmG	-1,5	1,7
VC_A0697	cdgD	-1,5	1,5

VC_A0088	-	-1,5	1,3
VC_2377	gltD	-1,6	1,5
VC_2436	tolC	-1,6	1,7
VC_A0068	-	-1,6	1,6
VC_A0443	blc	-1,6	1,6
VC_A1013	-	-1,6	1,4
VC_1439	ccoP	-1,7	1,6
VC_A0039	-	-1,7	2,8
VC_A0818	-	-1,7	1,3
VC_1952	chiA	-1,7	1,6
VC_A1029	-	-1,7	2,5
VC_0278	-	-1,7	2,6
VC_A0350	blc	-1,7	1,8
VC_1562	-	-1,7	2,5
VC_0136	rhtB	-1,7	1,5
VC_0866	mltF	-1,8	1,5
VC_A0804	deaD	-1,8	1,9
VC_1379	-	-1,8	1,3
VC_0968	cysK	-1,8	1,3
VC_2738	pck	-1,8	3,0
VC_A0459	-	-1,8	1,3
VC_A0621	-	-1,8	1,4
VC_1389	-	-1,8	1,4
VC_A1060	ribB	-1,9	1,4
VC_A0531	-	-1,9	2,0
VC_0610	fbpC	-1,9	1,3
VC_0049	smg	-1,9	2,1
VC_A0025	-	-1,9	1,5
VC_2534	-	-1,9	2,4
VC_1866	pflB	-1,9	2,6
VC_0116	hemN	-1,9	3,5
VC_0910	treB	-1,9	1,9
VC_2628	aroB	-1,9	1,5
VC_2529	rpoN	-1,9	1,5
VC_0321	tufA	-1,9	2,0
VC_A0629	-	-1,9	1,8
VC_1516	-	-2,0	1,6
VC_A0905	cdaR	-2,0	1,4
VC_0409	fimA	-2,0	1,5
VC_0909	treR	-2,0	1,3
VC_0967	-	-2,0	1,4

VC_0056	aroE	-2,0	1,3
VC_0574	petB	-2,0	2,5
VC_0947	dacA	-2,0	1,8
VC_0362	tufA	-2,0	1,9
VC_0234	wavI	-2,0	1,5
VC_1442	ctaD	-2,1	4,0
VC_0659	prfC	-2,1	2,3
VC_0026	yhdH	-2,1	1,6
VC_0993	nagC	-2,1	1,5
VC_2092	gltA	-2,1	1,4
VC_1894	lpoB	-2,2	1,7
VC_0002	mioC	-2,2	2,6
VC_A0877	pva	-2,2	1,8
VC_A0255	-	-2,2	1,6
VC_A0983	-	-2,2	2,6
VC_1509	npdA	-2,2	1,7
VC_1843	cydB	-2,3	3,1
VC_1778	siaQ	-2,3	1,4
VC_2701	dsbD	-2,3	2,9
VC_A1040	-	-2,3	1,8
VC_1046	fadI	-2,4	3,7
VC_0633	ompU	-2,4	2,5
VC_A0908	hutX	-2,4	1,5
VC_0061	thiC	-2,4	4,0
VC_A0425	-	-2,4	1,5
VC_A0514	-	-2,4	1,5
VC_2294	nqrB	-2,4	2,9
VC_0966	ptsH	-2,4	1,5
VC_A0026	-	-2,5	1,4
VC_A0999	-	-2,5	1,3
VC_2052	ccmF	-2,5	1,5
VC_2185	engD	-2,5	2,1
VC_2749	glnG	-2,5	2,1
VC_1630	-	-2,5	1,9
VC_1847	ruvC	-2,5	3,5
VC_0344	amiB	-2,5	2,6
VC_2415	pdhR	-2,5	2,7
VC_0190	uvrD	-2,6	3,3
VC_2637	prxA	-2,6	1,7
VC_0337	-	-2,6	1,8
VC_0547	lysC	-2,6	2,2

VC_0066	thiH	-2,6	2,1
VC_0400	mshJ	-2,6	1,9
VC_2156	-	-2,6	2,1
VC_0951	rlmH	-2,7	1,5
VC_2629	aroK	-2,7	1,8
VC_0260	wbeV	-2,7	2,3
VC_0695	aroF	-2,7	1,3
VC_2369	arcB	-2,8	3,2
VC_1911	pyrF	-2,8	2,2
VC_2466	rseA	-2,8	1,8
VC_1179	rluB	-2,9	2,7
VC_0745	suhB	-2,9	1,6
VC_2655	poxA	-2,9	1,8
VC_0419	rng	-2,9	1,6
VC_1517	-	-2,9	2,1
VC_1857	-	-2,9	1,6
VC_1810	-	-2,9	2,1
VC_2530	-	-2,9	1,5
VC_0065	thiG	-2,9	2,2
VC_2418	dsbC	-3,0	1,5
VC_0070	tsrA	-3,0	2,5
VC_A0549	-	-3,0	1,3
VC_1556	-	-3,0	1,4
VC_1215	pgsA	-3,0	1,4
VC_2463	lepA	-3,0	4,0
VC_0062	-	-3,0	3,2
VC_1318	ompV	-3,1	2,1
VC_0556	gshA	-3,2	2,0
VC_0106	-	-3,2	1,7
VC_0964	crr	-3,2	2,1
VC_2292	nqrD	-3,2	2,9
VC_2262	glnD	-3,2	2,5
VC_2351	-	-3,2	1,7
VC_0965	ptsI	-3,3	4,0
VC_A0777	-	-3,3	2,6
VC_2112	-	-3,3	2,9
VC_1839	tolQ	-3,4	1,6
VC_0236	waaF	-3,4	1,3
VC_0289	gntR	-3,4	1,3
VC_0369	rplI	-3,5	1,5
VC_A1022	-	-3,5	1,9

VC_0911	treA	-3,5	1,8
VC_2291	nqrE	-3,5	1,6
VC_2390	carA	-3,5	1,9
VC_A0380	-	-3,6	2,1
VC_2083	znuB	-3,6	1,5
VC_A0240	-	-3,6	1,8
VC_A0016	glgB	-3,7	4,0
VC_2067	fleN	-3,7	4,0
VC_0811	-	-3,8	1,6
VC_2320	recB	-3,8	1,4
VC_0991	asnB	-3,8	2,0
VC_0374	pgi	-3,8	3,1
VC_0525	-	-3,8	3,0
VC_1998	msrB	-3,9	2,8
VC_1820	-	-3,9	1,4
VC_1680	sapA	-3,9	3,1
VC_0003	mnmE	-3,9	2,5
VC_0485	pykF	-3,9	3,5
VC_1880	-	-4,0	1,4
VC_0709	rluD	-4,0	3,0
VC_0210	rph	-4,0	1,9
VC_2044	grxD	-4,1	1,3
VC_A0597	-	-4,1	2,3
VC_0395	gtaB	-4,1	2,0
VC_0108	polA	-4,2	3,0
VC_1190	-	-4,2	3,0
VC_0437	-	-4,2	1,5
VC_1004	purF	-4,2	4,0
VC_A1003	-	-4,2	2,4
VC_0276	purH	-4,3	4,0
VC_2290	nqrF	-4,3	3,1
VC_0932	rbmEF	-4,3	1,5
VC_0122	cyaA	-4,4	4,0
VC_1903	ftsK	-4,4	3,5
VC_2635	mrcA	-4,4	3,0
VC_A1094	-	-4,4	1,6
VC_0848	smpB	-4,4	1,4
VC_A0925	pyrC	-4,5	2,5
VC_2625	rpe	-4,6	2,7
VC_0275	purD	-4,6	4,0
VC_1015	rnfC	-4,6	1,4

VC_A0013	malP	-4,7	4,0
VC_A0014	malQ	-4,7	4,0
VC_1097	pta	-4,7	4,0
VC_A0152	mrpG	-4,7	4,0
VC_0052	purE	-4,8	1,6
VC_2389	carB	-4,9	4,0
VC_2153	-	-4,9	1,4
VC_2311	-	-5,0	2,0
VC_0869	purL	-5,0	4,0
VC_2226	purM	-5,0	3,2
VC_2307	-	-5,1	1,7
VC_1575	-	-5,1	4,0
VC_2272	nrdR	-5,1	1,8
VC_0671	rppH	-5,2	1,6
VC_SEN_bncRNA624	-	-5,2	1,3
VIBCH11745	-	-5,2	2,0
VC_0724	pstC	-5,2	3,3
VC_2152	dapE	-5,2	4,0
VC_A0897	pgl	-5,3	2,5
VC_1836	tolB	-5,3	2,7
VC_1639	-	-5,3	2,1
VC_2510	pyrB	-5,3	4,0
VC_2271	ribD	-5,3	4,0
VC_0894	thiI	-5,4	4,0
VC_2419	xerD	-5,4	4,0
VC_1887	csiV	-5,5	2,0
VC_1921	clpX	-5,5	3,5
VC_2266	thiL	-5,5	3,1
VC_0051	purK	-5,5	4,0
VC_0543	recA	-5,6	3,5
VC_A0668	-	-5,7	1,3
VC_0768	guaA	-5,8	4,0
VC_1263	ribA	-5,8	3,3
VC_A0002	rctB	-5,8	1,6
VC_1907	cysB	-5,8	2,1
VC_2441	-	-5,8	4,0
VC_0727	phoU	-5,9	1,8
VC_0238	wavK	-5,9	2,4
VC_1844	cydA	-5,9	4,0
VC_0767	guaB	-5,9	4,0
VC_2442	-	-6,0	4,0

VC_0868	-	-6,1	2,0
VC_0851	smpA	-6,3	2,1
VC_0211	pyrE	-6,3	3,7
VC_2270	ribE	-6,4	4,0
VC_2775	gidA	-6,6	4,0
VC_A0827	phhB	-6,6	1,5
VC_1922	clpP	-6,7	2,1
VC_2265	pgpA	-7,3	1,9

Table S5: strains

Strain	Name	Construction & remarks
E. coli MG1655 wt	C349	Lab collection
E. coli MG1655 ΔdusB	J243	Babosan et al, 2022
E. coli K-12 BW25113	G206	Lab collection
E. coli K-12 BW25113	J075	Lab collection
E. coli B2163	F656	Lab collection<br>auxotrophe DAP with pSC189 specR
N16961 hapR+	F606	Gift from Melanie Blokesch
N16961 hapR+ ΔlacZ	K329	Babosan et al, 2022<br>Strain used to construct the wild-type (WT) transposon mutant library
ΔdusB::kan (VC_0291) ΔlacZ	L606	Babosan et al, 2022
ΔdusB (VC_0291) ΔlacZ	T302	Kanamycin resistance cassette was removed from L606 strain, using FLP/FRT system (Zhu et al, 1995)<br>Strain used to construct the *ΔdusB* transposon mutant library
ΔdusA::kan (VC0379) ΔlacZ	L607	Babosan et al, 2022
ΔdusC::kan (VC_1105) ΔlacZ	W153	PCR amplification of 500 bp up and down regions of VC_1105: primers dusC6+dusC8 & dusC7+dusC5. PCR of frt::kan::frt kanamycin resistance cassette (Val et al PLoS Genetics 2012, Negro et al, mBio 2019): MV268+MV269. PCR assembly of VC_1105::kan: dusC6/dusC5. Allelic exchange by natural transformation.
ΔdusAC::kan (VC0379/VC_1105)	V702	See above : dusC was deleted in parental strain dusA
ΔdusAC (VC0379/VC_1105)	V705	Kanamycin resistance cassette in V702 was removed using FLP/FRT system (Zhu et al, 1995)
ΔdusABC::kan (VC0379/VC_0291/VC_1105)	V948	allelic exchange in V705 by integration and excision of conjugative suicide plasmid pMP7 L416, replacing the gene with frt::kan::frt (see Babosan et al, 2022)
ΔtruB::kan (VC_0645)	M562	Babosan et al, 2022
ΔtruB (VC_0645)	X748	Kanamycin resistance cassette was removed using FLP/FRT system (Zhu et al, 1995)
ΔtrmA::kan (VC_0154)	M564	Babosan et al, 2022
ΔtrmA (VC_0154)	X749	Kanamycin resistance cassette was removed using FLP/FRT system (Zhu et al, 1995)
ΔdusBΔtruB::kan (VC_0291/VC_0645)	X752	allelic exchange in T302 by integration and excision of conjugative suicide plasmid pMP7 M347, replacing the gene with frt::kan::frt (see Babosan et al, 2022)
ΔdusBΔtrmA::kan (VC_0291/VC_0154)	X753	allelic exchange in T302 by integration and excision of conjugative suicide plasmid pMP7 M423, replacing the gene with frt::kan::frt (see Babosan et al, 2022)
ΔtruBΔtrmA::kan (VC_0645/VC_0154)	X847	allelic exchange in M562 by integration and excision of conjugative suicide plasmid pMP7 M423, replacing the gene with frt::kan::frt (see Babosan et al, 2022)

Table S6: Plasmids

**Plasmids stored in *E. coli***	**Name**	**Primers**	**Construction**	**Refered to as**	**Storage**
empty pSC101	O896	/		p0	DH5α

pSC101 *Vc* wt promoter - *Vc dusB*	T893	ZIP932 + ZIP886	Sma1 + T4 DNA ligase	p*dusB Vc*	DH5α
pSC101 *Vc* wt promoter - *Ec dusB*	U368	promoter amplified from *Vc* with ZIP932 + ZIP994; dusB amplified from Ec with ZIP995+ZIP996; assembly: ZIP932+ZIP996	Sma1 + T4 DNA ligase	p*dusB Ec*	DH5α
pSC101 *Vc* wt promoter - *Vc dusB* m1 (P16A/M17A)	U362	ZIP974 + ZIP975	Inverse PCR on pT893	p*dusB Vc* m1	DH5α
pSC101 *Vc* wt promoter - *Vc dusB* m2 (K139A)	U363	ZIP976 + ZIP977	Inverse PCR on pT893	p*dusB Vc* m2	DH5α
pSC101 *Vc* wt promoter - *Vc dusB* m3 (C100A)	U364	ZIP978 + ZIP979	Inverse PCR on pT893	p*dusB Vc* m3	DH5α
pSC101 *Vc* wt promoter - Vc *dusB* m4 (K107/8E)	U365	ZIP980 + ZIP981	Inverse PCR on pT893	p*dusB Vc* m4	DH5α
pSC101 *Vc* wt promoter - Vc *dusB* m5 (K103/4E)	U366	ZIP982 + ZIP983	Inverse PCR on pT893	p*dusB Vc* m5	DH5α
pSC101 *Vc* wt promoter - Vc *dusB* m6 (K103/4E + R297/8E)	U367	ZIP984 + ZIP985	Inverse PCR on pU366	p*dusB Vc* m6	DH5α
pSC101 *Vc* gyrA promoter - *Vc cysH*	U732	ZIP1000 + ZIP1001	Sma1 + T4 DNA ligase	*pcysH*	DH5α
pSC101 *Vc* wt promoter - *Vc dusB* VcNN 9 (S205N)	Y316	ZIP1185 + ZIP1186	Inverse PCR on pT893	p*dusB* VcNN 9	DH5α
pSC101 *Vc* wt promoter - *Vc dusB* VcNN 10 (E272A)	Y317	ZIP1187 + ZIP1188	Inverse PCR on pT893	p*dusB* VcNN 10	DH5α
pSC101 *Vc* wt promoter - *Vc dusB* VcNN 15 (Q308R)	Y322	ZIP1197 + ZIP1198	Inverse PCR on pT893	p*dusB* VcNN 15	DH5α
pSC101 *Vc* wt promoter - *Vc dusB* VcNN 16 (A302T)	Y323	ZIP1220 + ZIP1221	Inverse PCR on pT893	p*dusB* VcNN 16	DH5α
pSC101 *Vc* wt promoter - *Vc dusB* VcNN 17 (dna : CCG-790-AC frameshift)	Y324	ZIP1222 + ZIP1223	Inverse PCR on pT893	p*dusB* VcNN 17	DH5α
pSC101 *Vc* wt promoter - *Vc dusB* VcNN 18 (Q308K)	Y325	ZIP1224 + ZIP1225	Inverse PCR on pT893	p*dusB* VcNN 18	DH5α
pSC101 *Vc* wt promoter - *Vc dusB* VcNN 19 (M176I)	Y326	ZIP1226 + ZIP1227	Inverse PCR on pT893	p*dusB* VcNN 19	DH5α
pSC101 *Vc* wt promoter - *Vc dusB* VcNN 20 (E180K)	Y327	ZIP1228 + ZIP1229	Inverse PCR on pT893	p*dusB* VcNN 20	DH5α
pSC101 *Vc* wt promoter - *Vc dusB* VcNN 21 (E289K)	Y328	ZIP1230 + ZIP1231	Inverse PCR on pT893	p*dusB* VcNN 21	DH5α

Table S7: Primers and genes

Name	Primer sequence (5' to 3')	Usage
ZIP886	TTATGATGCAACGTTATCAAAATACCCTTCGAGCGC	See Table S6
ZIP932	tcatcaaaggactcctgaagc	See Table S6
ZIP974	GCAGCGgcgggtgtcaccgacagaccct	See Table S6
ZIP975	ggcgacgatcagattgttctt	See Table S6
ZIP976	GCAacccgaacgggttgggataca	See Table S6
ZIP977	gagtgtgactggcacattcact	See Table S6
ZIP978	GCCcccagccaaaaaagtaaataaa	See Table S6
ZIP979	gcccatgttgatatcgatgat	See Table S6
ZIP980	GAAGAGctcgcgggttctgcactgctg	See Table S6
ZIP981	atttactttttggctgggcag	See Table S6
ZIP982	GAAGAAgtaaataaaaagctcgcgggtt	See Table S6
ZIP983	ggctgggcagcccatgttga	See Table S6
ZIP984	GAGGAGaccttcaacgccattgagact	See Table S6
ZIP985	aaacgcactcgcttgttcatg	See Table S6
ZIP994	aacgtcttcacagcttcgtacca	See Table S6
ZIP995	tggtacgaagctgtgaagacgtt ATGgGCATCGGACAATATCAGCT	See Table S6
ZIP996	TTACGCAAAATTTTCGAAGTATGC	See Table S6
ZIP1000	cgactgacttggcgctcaatcttgtagtgagcttcgtttcagtaagaatttgggtataccgatcaaactatagagggataatggctct atgcctaatcgtaccgttcc	See Table S6
ZIP1001	ttattgatgtgtatcgttatgatcttcg	See Table S6
ZIP1185	TTATCGATATCACCGTTAGCGATAACCG	See Table S6
ZIP1186	CCCGGAGAAAGCACGCTACG	See Table S6
ZIP1187	GCTCCATAAAACTGATGGAGTGCGGTT	See Table S6
ZIP1188	GTACTTAGGCCCACGAATTGCGC	See Table S6
ZIP1197	CGGTCAGCAGTCTCAATGGCGTT	See Table S6
ZIP1198	GCAACTCGATGCGCTCGAAG	See Table S6
ZIP1199	AGGTCGACTCTAGAGGATCCCC gtaatgctccaatccgcgttctg	See Table S6
ZIP1200	ggcgcttgtcactattgatcact tgatagaaagaatggggggatggt	See Table S6
ZIP1201	accatccccccattctttctatcaagtgatcaatagtgacaagcgcc	See Table S6
ZIP1202	TGTCGGAATTCGAGCTCGGTACCC aatttattcgccgtacaccaccaa	See Table S6
ZIP1204	AGGTCGACTCTAGAGGATCCCC cttaccgctgcgcgtcagt	See Table S6
ZIP1205	TGTCGGAATTCGAGCTCGGTACCC cggttactttttgcgctctaacatc	See Table S6
ZIP1216	tgtggtaaggaaatcgtggcaa	See Table S6
ZIP1217	ttacacacctaactcttggtaagggatct	See Table S6
ZIP1220	Accattgagactgctgaccag	See Table S6
ZIP1221	gttgaaggtacggcgaaacgc	See Table S6
ZIP1222	ACcactccatcagttttatggagagtactt	See Table S6
ZIP1223	ttacatgaccaagcatgatgtctttcac	See Table S6
ZIP1224	Aagcaactcgatgcgctcgaa	See Table S6
ZIP1225	gtcagcagtctcaatggcgt	See Table S6

ZIP1226	Atacaaaggtgaggcggaatacgaca	See Table S6
ZIP1227	atgcaggcacgagttcgtcca	See Table S6
ZIP1228	Aaggcggaatacgacagcatt	See Table S6
ZIP1229	acctttgtacatgcaggcacg	See Table S6
ZIP1230	Aagcatgaacaagcgagtgcgtt	See Table S6
ZIP1231	ttgcaggtaccaactcacgtgt	See Table S6
dusC5	CTATTATTTAAACTCTTTCCGGTAAACAGCTCTGGTAGCC	See Table S6
dusC6	TACGTAGAATGTATCAGACTCAGATGCCGTGGCACGCT	See Table S6
dusC7	CTACACAATCGCTCAAGACGTGAGTGATGAATACCGGCTAGC	See Table S6
dusC8	CTAATTCCCATGTCAGCCGTGAGTAAAACAACGAATCAGTA	See Table S6
MV268	CACGTCTTGAGCGATTGTGTAG	See Table S6
MV269	ACGGCTGACATGGGAATTAG	See Table S6
BC0017	CAAGCAGAAGACGGCATACGAGATAACCATTCTCGTGACTGGAGTTCAGACGTGTGCTCTTCCGATCT	Tn-seq PCR2 to amplify and uniquely barcode V. cholerae WT (K329) T0 replicate 1
BC0018	CAAGCAGAAGACGGCATACGAGATGGTTGCCTCTGTGACTGGAGTTCAGACGTGTGCTCTTCCGATCT	Tn-seq PCR2 to amplify and uniquely barcode V. cholerae WT (K329) T0 replicate 2
BC0007	CAAGCAGAAGACGGCATACGAGATAGAGCACTAGGTGACTGGAGTTCAGACGTGTGCTCTTCCGATCT	Tn-seq PCR2 to amplify and uniquely barcode V. cholerae WT (K329) T0 replicate 3
BC0019	CAAGCAGAAGACGGCATACGAGATCTAATGATGGGTGACTGGAGTTCAGACGTGTGCTCTTCCGATCT	Tn-seq PCR2 to amplify and uniquely barcode V. cholerae WT (K329) T16 replicate 1
BC0020	CAAGCAGAAGACGGCATACGAGATTCGGCCTATCGTGACTGGAGTTCAGACGTGTGCTCTTCCGATCT	Tn-seq PCR2 to amplify and uniquely barcode V. cholerae WT (K329) T16 replicate 2
BC0008	CAAGCAGAAGACGGCATACGAGATTGCCTTGATCGTGACTGGAGTTCAGACGTGTGCTCTTCCGATCT	Tn-seq PCR2 to amplify and uniquely barcode V. cholerae WT (K329) T16 replicate 3
BC0052	CAAGCAGAAGACGGCATACGAGATTCTTACATCAGTGACTGGAGTTCAGACGTGTGCTCTTCCGATCT	Tn-seq PCR2 to amplify and uniquely

		barcode ΔdusB T0 replicate 1
BC0053	CAAGCAGAAGACGGCATACGAGATTTACAATTCCGTGACTGGAGTTCAGACGTGTGCTCTTCCGATCT	Tn-seq PCR2 to amplify and uniquely barcode ΔdusB T0 replicate 2
BC0054	CAAGCAGAAGACGGCATACGAGATAAGCTTATGCGTGACTGGAGTTCAGACGTGTGCTCTTCCGATCT	Tn-seq PCR2 to amplify and uniquely barcode ΔdusB T0 replicate 3
BC0055	CAAGCAGAAGACGGCATACGAGATTATTCCTCAGGTGACTGGAGTTCAGACGTGTGCTCTTCCGATCT	Tn-seq PCR2 to amplify and uniquely barcode ΔdusB MH T16 replicate 1
BC0056	CAAGCAGAAGACGGCATACGAGATCTCGTGCGTTGTGACTGGAGTTCAGACGTGTGCTCTTCCGATCT	Tn-seq PCR2 to amplify and uniquely barcode ΔdusB MH T16 replicate 2
BC0057	CAAGCAGAAGACGGCATACGAGATTTAGGATAGAGTGACTGGAGTTCAGACGTGTGCTCTTCCGATCT	Tn-seq PCR2 to amplify and uniquely barcode ΔdusB MH T16 replicate 3
pSC189_PCR1	CCGTCAAGCCGTCAATTGTCTGATTCG	Tn-seq PCR1 (binds transposon end)
pSC189_PCR2	AATGATACGGCGACCACCGAGATCTACACTCTTTATCCATCACACTGGCGGCCGCGAAGTTC	Tn-seq PCR2 (Illumina adapter-compatible)
olj376	GTGACTGGAGTTCAGACGTGTGCTCTTCCGATCTGGGGGGGGGGGGGGGG	Tn-seq PCR1 to anchor the poly-C tail
**Name**	**Probe sequence**	
tRNA Gly-GCC	5' - ACCTCAACCTTGGCAAGGTTGCGC - 3'DIG	Northern blot
tRNA Ala-TGC	5' - CTGCGTGCAAGGCAGGCGCTCTCC - 3'DIG	Northern blot
tRNA Ser-GCT	5' - GCCTTCAGCCTCTCGGCCACCTCA - 3'DIG	Northern blot
Name	Syntesized DNA	
Napstar	TTGACAATTAATCATCCGGCTCGTATAATGTGTGGAATTGTGAGCGGATAACAATTTCACAC<br>AGGAAACAGCGCCGCATG GTT GCA AGT AAG GTC CCT GAG GCC GCT ATT AGC CGT<br>CTG ATC ACT TAC CTG CGC ATT CTT GAA GAA CTG GAA GCG CAG GGT GTA CAT CGC<br>ACC GCC TCC GAA CAG TTA GGC GAA CTT GCC CAA GTG ACG GCT TTT CAG GTA GAC<br>AAG GAT CTG AGC TAC TTT GGT AGT TAT GGC ACT GAC GGC GTA GGA TAT ACT GTG<br>CCG GTT CTT AAG CGT GAA TTA CGC CAT ATC TTG GGC CTG AAC CGC AAA TGG GGC<br>CTT TGC ATT GTT GGA ATG GGC CGC CTG GGC TCC GCG CTG GCG GAC TGG CCG<br>GGT TTT GGC GAA AGC TTC GAA CTG CGT GGC TTT TTC TCA CGC AGT GCA CAG AAA<br>GTT GGT CGC CCG GTT CGC GGC GGC GTC ATT GAA CAT GTA GAC CTG CTT CCG CAA	Synthesized by Twist biosciences

CGT GTG CCG GGC CGT ATC GAG ATC GCT CTT CTG ACC GTG CCC CGC GAA GCA
GCG CAG AAA GCA GCA GAT CTG CTG GTC GCG GCT GGC ATT AAA GGC ATC TTA AAC
TTT GCG CCG GTC GTT CTG GAA GTC CCT AAG GAA GTG GCA GTC GAG AAT GTT GAT
ATT TTG GCT GGC TTA ACA CGC TTG AGC TTC GCC ATT CTG AAT CCC ACC TGG TCA
GCG GCC GGC GGG CAC GGC TTT ACC GCG CAC AAC GTT TAC ATT ATG GCC GAC
AAA CAG AAA AAC GGT ATT AAA GCC AAT TTT AAG ATC CGT CAT AAT ATT GAA GAT GGT
GGT GTG CAA CTG GCC GAC CAC TAT CAG CAG AAC ACC CCC ATT GGT GAT GGC
CCG GTG CTG TTA CCG GAC AAC CAT TAT CTG AGC ATC CAG TCA AAA TTA AGT AAA
GAC CCG AAC GAA AAG CGT GAT CAC ATG GTA TTG CTG GAG TTT GTG ACG GCA GCT
GGC ATT ACC CAT GGT ATG GAT GAA TTA TAT AAA GGT GGC ACT GGC GGG TCA ATG
GTG AGT AAA GGG GAA GAG CTG TTT ACT GGC GTT GTC CCA ATT TTG GTG GAA TTG
GAC GGT GAT GTG AAC GGT CAC AAG TTC TCT GTC TCC GGA GAG GGT GAG GGC GAT
GCC ACC TAC GGT AAA CTG ACC CTG AAG TTC ATC TGT ACA ACC GGC AAA CTG CCC
GTC CCG TGG CCC ACC TTA GTG ACG ACG TTC AGC TAC GGG GTG ATG GTC TTC
GCG CGC TAT CCG GAT CAT ATG AAG CAG CAC GAC TTC TTC AAA AGT GCA ATG CCG
GAA GGT TAT GTG CAG GAA CGC ACA ATT TTC TTC AAA GAT GAT GGC AAT TAC AAA
ACG CGC GCC GAA GTG AAA TTT GAA GGA GAT ACT CTG GTA AAC CGC ATT GAA TTA
AAG GGG ATT GAC TTT AAA GAA GAT GGT AAT ATT TTA GGA CAT AAA TTG GAA TAT AAC
ACA AAG GTG CCC GAG GCG GCG ATT AGT CGT CTG ATT ACA TAT TTG CGT ATT CTG
GAA GAA CTG GAG GCC CAG GGT GTT CAT CGC ACC GCG TCT GAA CAG TTA GGT GAA
CTG GCC CAA GTT ACC GCC TTT CAG GTC GAT GAA GAT TTA TCG TAC TTC GGA TCC
TAC GGT ACG GAT GGT GTG GGC TAC ACG GTG CCG GTC CTT AAA CGT GAG CTT CGC
CAT ATT CTG GGC CTT AAC CGC AAA TGG GGT CTG TGC ATC GTG GGT ATG GGC CGC
CTT GGG TCT GCT CTG GCA GAT TGG CCC GGT TTT GGT GAG TCG TTT GAA CTT CGT
GGC TTC TTC AGC CGC TCG GCA CAG AAG GTA GGC CGC CCC GTC CGC GGT GGG
GTC ATT GAA CAT GTT GAT CTG TTA CCG CAA CGC GTT CCG GGT CGT ATT GAA ATC
GCG CTT TTA ACC GTG CCG CGC GAA GCA GCG CAG AAG GCC GCG GAC CTT CTG
GTG GCA GCC GGA ATC AAG GGA ATT CTG AAT TTT GCG CCC GTC GTG CTG GAG GTG
CCG AAA GAA GTT GCA GTG GAG AAC GTG GAT TTC CTT GCC GGT CTG ACC CGC CTG
TCC TTC GCG ATC CTG AAC CCA AAA TGG CGC GAA GAA ATG ATG GGT AGC GGC ACC
GGC GGA AAC GCA TCA GAT GGC GGG GGT AGC GGT GGG ATG GTG AGC AAG GGA
GAA GAA GAT AAT ATG GCG ATC ATT AAA GAA TTC ATG CGC TTT AAA GTA CAT ATG
GAG GGC AGC GTC AAT GGC CAT GAA TTC GAG ATT GAG GGC GAG GGC GAG GGC
CGT CCA TAC GAA GGA ACG CAG ACC GCA AAA CTG AAA GTC ACC AAA GGC GGC
CCT TTA CCG TTC GCG TGG GAT ATC CTG AGT CCG CAG TTT ATG TAT GGC TCG AAA
GCG TAT GTG AAA CAT CCG GCG GAT ATC CCG GAC TAT CTG AAA CTG TCT TTT CCG
GAG GGC TTT AAG TGG GAG CGT GTC ATG AAT TTT GAA GAT GGT GGC GTC GTA ACT
GTC ACG CAG GAC TCA AGT CTG CAG GAT GGG GAA TTC ATT TAT AAA GTC AAA CTG
CGC GGT ACA AAC TTT CCA AGC GAT GGC CCT GTG ATG CAA AAA AAG ACG ATG GGC
TGG GAG GCA TCG TCA GAA CGT ATG TAT CCG GAG GAT GGC GCG CTG AAA GGC
GAA ATC AAA CAA CGC CTT AAA TTG AAA GAC GGC GGC CAT TAC GAT GCC GAA GTT
AAG ACC ACA TAC AAG GCA AAG AAA CCC GTG CAG TTG CCA GGC GCC TAT AAC GTC
AAT ATT AAG CTG GAT ATC ACC AGC CAC AAC GAA GAT TAT ACG ATC GTG GAG CAG
TAT GAA CGC GCG GAG GGA CGT CAC AGC ACA GGT GGG ATG GAC GAG TTG TAC
AAG TAA
